# Gene Regulation and Epigenetic Remodeling in Murine Embryonic Stem Cells by c-Myc

**DOI:** 10.1371/journal.pone.0007839

**Published:** 2009-11-13

**Authors:** Chin-Hsing Lin, ChenWei Lin, Hisashi Tanaka, Matthew L. Fero, Robert N. Eisenman

**Affiliations:** 1 Division of Basic Sciences, Fred Hutchinson Cancer Research Center, Seattle, Washington, United States of America; 2 Clinical Research Division, Fred Hutchinson Cancer Research Center, Seattle, Washington, United States of America; 3 Department of Molecular Genetics, Cleveland Clinic Foundation, Cleveland, Ohio, United States of America; Roswell Park Cancer Institute, United States of America

## Abstract

**Background:**

The Myc oncoprotein, a transcriptional regulator involved in the etiology of many different tumor types, has been demonstrated to play an important role in the functions of embryonic stem (ES) cells. Nonetheless, it is still unclear as to whether Myc has unique target and functions in ES cells.

**Methodology/Principal Findings:**

To elucidate the role of c-Myc in murine ES cells, we mapped its genomic binding sites by chromatin-immunoprecipitation combined with DNA microarrays (ChIP-chip). In addition to previously identified targets we identified genes involved in pluripotency, early development, and chromatin modification/structure that are bound and regulated by c-Myc in murine ES cells. Myc also binds and regulates loci previously identified as Polycomb (PcG) targets, including genes that contain bivalent chromatin domains. To determine whether c-Myc influences the epigenetic state of Myc-bound genes, we assessed the patterns of trimethylation of histone H3-K4 and H3-K27 in mES cells containing normal, increased, and reduced levels of c-Myc. Our analysis reveals widespread and surprisingly diverse changes in repressive and activating histone methylation marks both proximal and distal to Myc binding sites. Furthermore, analysis of bulk chromatin from phenotypically normal c-*myc* null E7 embryos demonstrates a 70–80% decrease in H3-K4me3, with little change in H3-K27me3, compared to wild-type embryos indicating that Myc is required to maintain normal levels of histone methylation.

**Conclusions/Significance:**

We show that Myc induces widespread and diverse changes in histone methylation in ES cells. We postulate that these changes are indirect effects of Myc mediated by its regulation of target genes involved in chromatin remodeling. We further show that a subset of PcG-bound genes with bivalent histone methylation patterns are bound and regulated in response to altered c-Myc levels. Our data indicate that in mES cells c-Myc binds, regulates, and influences the histone modification patterns of genes involved in chromatin remodeling, pluripotency, and differentiation.

## Introduction

ES cells must be capable of self-renewal while simultaneously retaining the capacity to commit to a wide range of differentiation lineages. The notion that the determination and maintenance of embryonic stem (ES) cell pluripotency and self-renewal is related to an epigenetic state characterized by an open chromatin conformation has received considerable support over the last several years [1]–[6]. Open chromatin is thought to contribute to pluripotency by permitting relatively broad accessibility to transcriptional regulation and is itself likely to be the result of diverse activities including nucleosome assembly, positioning, and remodeling, incorporation of histone variants, binding of chromatin modifying factors, epigenetic modifications, sub-nuclear compartmentalization, and other dynamic processes that maintain active chromatin (for reviews see [2], [7], [8]).

Much recent work on ES cell pluripotency has focused on two aspects of transcriptional regulation: the actions of the Sox2-Oct4-Nanog transcription factor network and the nature of epigenetic changes associated with pluripotency [9]. The Sox2-Oct4-Nanog transcription factors have been known for about a decade to be required for early embryonic development and for ES cell self-renewal [10]–[13]. Genome-wide binding analyses have indicated that in both human and murine ES cells the Sox2, Oct4, and Nanog factors occupy hundreds of gene promoters [14], [15]. Importantly, these gene targets include many developmental regulators, a subset of which, encoding transcription factors and chromatin modifying activities, are associated with RNA polymerase II and are expressed in ES cells. A second subset of Sox2-Oct4-Nanog bound genes are involved in lineage-specific differentiation – these genes are associated with Polycomb complex components (including Suz12, Eed, EZH2) and are repressed in ES cells [16]–[18]. Therefore the Sox2-Oct4-Nanog factors are arguably functioning as selectors of genes whose activation or repression in ES cells are critical for pluripotency and self-renewal.

It is likely that one reflection of the open chromatin conformation proposed for ES cells is the relative paucity of epigenetic marks associated with gene repression. This includes, in comparison to non-pluripotent cells, decreased DNA methylation and histone H3 lysine 27 trimethylation (H3-K27me3) as well as augmentation of positive marks such as histone H4 acetylation and H3-K4me3 [19], [20] (for review see [3]). Nonetheless, the association of Polycomb complexes with a subset of Sox2-Oct4-Nanog bound genes that are important for cell fate transitions [16]–[18] would suggest that a highly regulated form of repression must be important in self-renewing ES cells. Indeed several studies have confirmed the presence of H3-K27me3 at the promoters of Polycomb bound loci, many of which overlap with Sox2-Oct4-Nanog binding sites. Interestingly, a subgroup of these promoters contain islands of H3-K4me3 within a larger domain of H3-K27me3, constituting what has been termed a ”bivalent“ chromatin structure [20]. A number of these bivalent genes lose Polycomb binding and H3-K27 methylation upon differentiation, leading to the proposal that bivalency marks genes that are poised for activation upon ES cell differentiation [16], [20]–[23]. Such bivalent domains are not restricted to ES cells and their formation and resolution is likely to be a widespread and dynamic process [21]–[23]. While loss of function of Polycomb subunits and abolishment of H3-K27me3 results in upregulation of differentiation-related bivalent genes in ES cells, these cells continue to self-renew, indicating that Polycomb-mediated repression of these genes is not the sole determinant of the transcriptional regulation that maintains the undifferentiated state [24], [25]. These experiments prompt questions concerning the extent to which histone modifications represent a cause or a consequence of pluripotency. A related issue concerns the precise mechanisms through which widespread epigenetic changes occur. Recently the histone H3-K9 demethylases Jmjd1a and Jmjd2c have been shown to be targets of Oct4 and are implicated in the activities of the Sox2-Oct4-Nanog network and the maintenance of pluripotency-related gene expression [26]. However it is still unclear how these and other histone modifying complexes are recruited to specific gene targets in ES cells.

Another transcriptional regulatory protein that has been connected with ES cell function is Myc. The *myc* oncogene family encodes the c-, N- and L-Myc, bHLHZ proteins that heterodimerize with Max and bind DNA at E-box sequences. Myc family proteins have been associated with cell growth and proliferation through widespread binding to DNA (on the order of 10–20% of genomic loci are bound) and modulation of the expression of hundreds to thousands of genes [27]–[31]. These include genes encoding ribosomal RNA and ribosomal proteins as well as many factors involved in translation and metabolism [32]–[36]. *myc* gene expression is itself regulated by many different mitogens, cytokines, and growth factors, suggesting that Myc serves as an integrator of signal transduction. As such, Myc proteins are likely to link extracellular signals to an acute cellular growth response [37]–[39]. It is therefore unsurprising that Myc is expressed in different types of stem cells including ES cells [40]–[43]. Yet there are several lines of evidence indicating that Myc may function as a regulator of pluripotency. For example, although Myc expression has long been associated with the inhibition of terminal differentiation, recent studies have indicated that it can also drive diverse progenitor cell types down differentiation pathways [44]–[46]. Moreover, c-*myc* loss of function in hematopoietic stem cells leads to their retention in the stem cell niche and inhibition of differentiation [47] while deletion of both N- and c-*myc* results in their ablation [48] . Importantly, in ES cells c-Myc appears to be critical in maintaining self-renewal and inhibiting differentiation [41], [49]. Moreover, *myc* family genes have been shown to cooperate with Sox2, Oct3/4 and Klf4 in the conversion of murine embryo fibroblasts (MEFs) into induced pluripotent stem cells (iPS) [50]–[52] (for review see [9]). In the generation of iPS cells *myc* can be replaced by other factors but at the cost of greatly diminished efficiency [53], [54]. Fibroblasts reprogrammed by Myc, Oct4, Sox2 and Klf4 display widespread epigenetic changes characteristic of the chromatin in ES cells [55]. In this regard it is interesting that *myc* loss of function has been associated with a reversible global change in histone acetylation and methylation [56]. Furthermore induction of c-Myc in a B cell line results in increased histone H3 and H4 acetylation at a defined group of Myc target genes [57]. Reasoning that Myc may have gene targets and functions that are unique to ES cells we have carried out a genome location (ChIP-chip) analysis of c-Myc binding in mES cells and examined the consequences of altering Myc levels on histone methylation. Our data indicate that Myc is involved in the transcriptional regulation of several members of the ES cell pluripotency network and in widespread histone methylation mediated by the Polycomb complex.

## Results

### Identification of Genes Bound and Regulated by c-Myc in ES Cells

**Figure 1:**
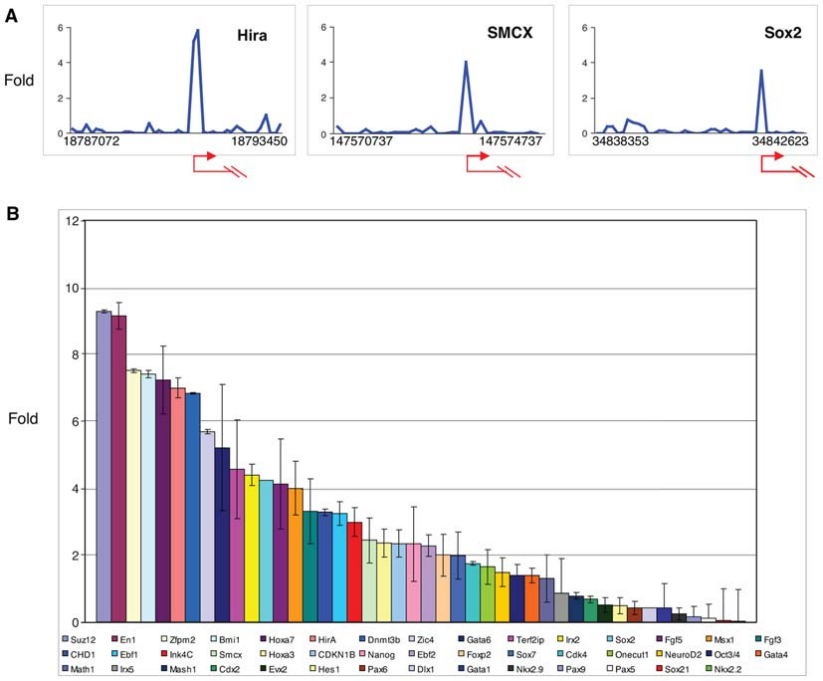
c-Myc-bound promoters in mouse embryonic stem cells identified by ChIP-chip analysis and validated by qChIP-PCR. (A) Examples of three gene promoter regions identified using chromatin immunoprecipitation with anti-c-Myc antibody to probe DNA microarrays (see Methods and Materials for details). Shown are the fold enrichment ratios for anti-Myc ChIP-enriched versus total input genomic DNA (y axis) for all probes within the genomic regions indicated. Numbers represent the beginning and the end probe positions (x axis). The transcriptional start sites and direction of transcription are noted by arrows. (B) Validation by qChIP-PCR of putative c-Myc target genes in AK7 mES cells. Forty-four genes were selected at random for validation and included c-Myc target and non-c-Myc target genes as determined from the initial promoter array. Equal amounts of anti-c-Myc ChIP DNA and total input DNA were used for quantitative PCR employing SYBR Green detection with an ABI7900HT system. Bar heights represent the average fold enrichment ratios from 2 independent sets of anti-Myc ChIP-enriched versus total input genomic DNA. qChIP-PCR data derived for R1 mES cells is shown in Supplementary Figure S2).

We mapped genomic binding sites for endogenous c-Myc in murine embryonic stem (ES) cells by employing DNA derived from anti-Myc chromatin-immunoprecipitation to probe DNA microarrays (ChIP-chip) comprised of 21,632 mouse gene promoters. For these experiments we employed sorted SSEA-1 positive R1 and AK7 ES cells and a pre-absorbed antibody with reactivity restricted to c-Myc protein (see Materials and Methods). We imposed a cut-off of four-fold enrichment following chromatin immunoprecipitation with anti-Myc compared to input, and also applied the widely used ACME/R analysis software to identify significant peaks across gene promoters by assigning a probability value (p-value) to each probe in the array (we used p-value<0.0001 as our cut-off) [58], [59] (see Materials and Methods and Figure. S1, Table S1). Using these approaches we identified 3189 promoter binding sites representing 12.8% of promoters analyzed. Examples of enrichment profiles (ChIP-enriched versus total genomic DNA) observed for Myc binding within a ∼5 kb region of the transcriptional start sites of the genes encoding the histone chaperone HirA, the histone demethylase SMCX, and the Sox2 transcription factor are shown in Figure 1A (see Table S1 for list of c-Myc-bound genes). The percentage of c-Myc bound promoters identified is comparable with earlier promoter and genomic binding experiments in Drosophila and mammalian cells [27]–[29], [31]. We further validated association of c-Myc with a subset of the genomic loci identified in our ChIP-chip experiments by employing qChIP-PCR in four independent experiments with c-Myc antibody, carried out using two different ES cells lines (AK7 and R1) (Figure 1B, Figure S2). We randomly selected 44 genes for validation. Nineteen of these genes (Dlx-1, Evx2, Nkx2.2, Nkx2.9, Gata1, Pax5, Sox21, Sox7, Pax9, Pax6, Onecut1, Hes1, Oct3/4, Gata4, Cdx2, Mash1, Irx5, Math1, Cdk4) were not bound by Myc in the ChIP-chip assay and did not meet our criteria (>2 fold enrichment) for Myc binding in the qChIP-PCR. Two genes (Irx2 and Msx1) were negative in our ChIP-chip assay but positive in qChIP-PCR, and one gene (NeuroD2) was positive in the ChIP-chip assay but fell below the cut-off in the qChIP-PCR. The remaining genes were positive in both assays (Figure 1B). From these data we calculate an average false discovery rate of 6.8% AK7 ES cells and 11.3% in R1 ES cells.

**Figure 2:**
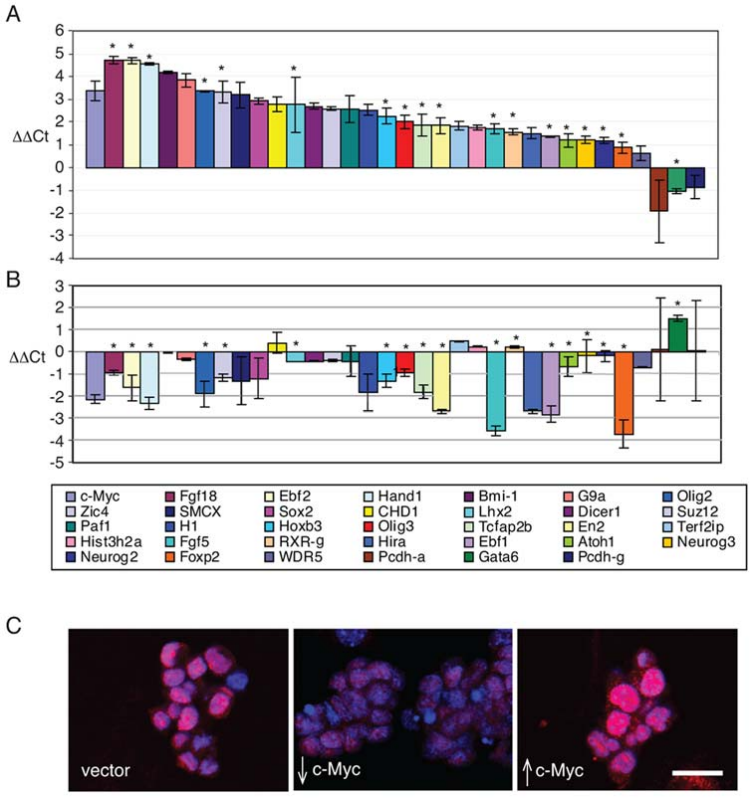
Transcriptional response of c-Myc target genes to c-Myc overexpression or knockdown. (A) Response of a subset of c-Myc target genes to Lentiviral mediated overexpression c-Myc in mES cells as determined by Real-Time PCR. Each bar represents the average (ΔΔCt) from triplicate sets of experiments on a log2 scale after normalization to internal controls and vector alone. The fold differences detected range from ∼2–20 fold. Note an approximately eight-fold increase in c-*myc* RNA levels (bar at far left). Color-coded genes are read from left to right (inset). (B) Response of a subset of c-Myc target genes to RNAi mediated knock-down of c-Myc in mES cells as determined by Real-Time PCR. Each bar represents the average (ΔΔCt) from a triplicate set of experiments on a log2 scale after normalization to internal controls and vector alone. c-*myc* RNA levels decreased 4 fold (bar at far left). Color-coded genes are read from left to right (inset). * indicates previously identified bivalent gene. (C) Sox2 immunostaining in mES cells. mES cells were infected with empty lentiviral vector alone (WT; left panel); lentiviral vector expressing shRNA against c-*myc* (middle panel); lentiviral vector expressing c-*myc* (right panel). Scale bar indicates 50 µm See Supplementary Figure S4 for immunoblots of Sox2 protein.

To determine whether c-Myc regulates the levels of expression of genes identified in our analysis we examined the transcript levels of a 33 gene subset of the promoters identified as bound by c-Myc in our ChIP-chip experiments (Figure 2A, B). We manipulated c-Myc abundance in ES cells by employing Lentiviral vectors to drive overexpression of c-*myc*, or to introduce shRNAs designed to knockdown c-*myc* expression. We observed an 8-fold average increase in c-*myc* RNA levels following infection with c-*myc* expressing Lentiviruses and a 4-fold decrease in c-*myc* RNA after introduction of shRNA directed against c-*myc* (Figure 2A, B, far left). Myc protein levels were also shown to respond to introduction of the c-*myc* shRNA and overexpression vectors [60]. These changes in c-Myc protein level induced altered proliferation rates where increased c-Myc resulted in increased BrdU incorporation and decreased population doubling time, while c-Myc knockdown has the opposite effect (Figure S3). Nearly 90% (30/33) of the c-Myc target genes examined showed a two-fold or greater augmentation of expression following c-*myc* overexpression (Figure 2A). Twenty-four genes in this group displayed decreased levels of expression subsequent to c-*myc* knockdown (Figure 2B). Of the 33 genes examined, three genes (Gata6, Pcdh-α. Pcdh-γ) appeared to be either marginally or substantially downregulated upon c-*myc* overexpression (Figure 2A). GATA 6 also displayed increased expression upon c-*myc* knockdown, indicating that it is repressed by c-Myc. This is consistent with considerable data showing that *myc* has a repressive, as well as an activating, role in transcription (for review see [61]).

### c-Myc Associates with ES Cell Genes Involved in Chromatin Structure and Pluripotency

Ontology analysis of genes associated with endogenous c-Myc in ES cells indicates, as expected from previous studies, that they fall into multiple functional classes involving growth, metabolism, cell signaling pathways, cell cycle progression, and apoptosis.

**Figure 3:**
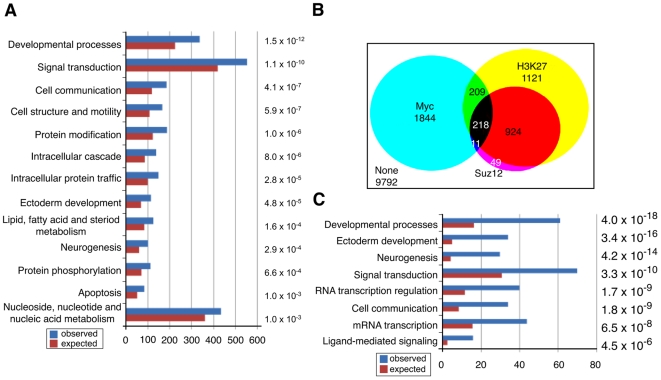
c-Myc, and Polycomb proteins share target genes. (A) Gene Ontology categories of c-Myc associated genes in murine ES cells. Functional categories derived for genes enriched at least 4-fold for c-Myc binding and possessing an ACME p<0.0001 in the ChIP-chip assay (see Materials and Methods and Supplementary Table S1). The numbers to the right represent the p-values for the statistical over-representation in each category. (B) Venn diagram depicting the overlap among murine ES cell genes bound by c-Myc (this paper), Suz12, and those displaying trimethylation of H3-K27 [16]. Nearly 96% of promoters bound by Suz12 also show the H3-K27me3 mark. Of genes associated with Myc (2282) 17% are associated with the H3-K27me3 mark and 9.5% associated with both Suz12 and H3-K27me3 (for gene list see Supplementary Table S3). (C) Gene ontology categories of gene displaying overlap among c-Myc, Suz12 binding and H3-K27me3 marks. Functional categories were derived for genes enriched at least 4-fold for c-Myc binding in the ChIP-chip assay (see Methods and Supplementary Table S1). Suz12 and H3-K27me3 bound genes were previously described [16]. Numbers to the right are p-values for the statistical over-representation among genes within a functional category.

In mES cells we also detect c-Myc binding to genes involved in developmental processes (e.g. neurogenesis and ectoderm development) (see Figure 3A) as well as genes involved in chromatin structure, such as the histone chaperone HirA, the histone lysine demethylase SMCX, and the SWI/SNF related SMARC genes (see Figure 1, Table S1). Such genes have not been previously linked to c-Myc binding or regulation and likely represent mES cell-specific c-Myc targets. Of particular interest is our finding that the *sox2* gene is bound by c-Myc proximal to the transcription start site (TSS) in ES cells (Figure 1A), an interaction confirmed by direct qChIP-PCR (Figure 1B). Moreover, *sox2* expression increases ∼8-fold in response to c-*myc* overexpression and decreases 2-fold when endogenous c-*myc* expression is reduced by shRNA (Figure 2A, B). As expected, we also observe changes in Sox2 protein level following modulation of c-Myc expression (Figure 2C, Figure S4). Because Sox2 and its binding partner Oct3/4 are key components of a transcription factor network that can induce pluripotency in human and murine fibroblasts [51]–[53] it is possible that Myc participates in this network, at least in part, through its ability to stimulate Sox2 expression.

### c-Myc Associates with a Subset of Polycomb-Regulated and Bivalent Genes in ES Cells

In *Drosophila*, d*myc* and *polycomb* (PcG) interact genetically [62] and genes in the trithorax group, that oppose PcG function, have been demonstrated to interact with dmyc genetically and physically [63], [64]. To test the hypothesis that c-Myc impinges on PcG function in mES cells, we compared c-Myc and PcG binding more extensively. In ES cells, PcG complex binding has been previously identified through genome-wide location analysis and found to associate with, and transcriptionally repress developmental regulatory genes [16], [18]. We compared our mES c-Myc binding data and the mES PcG ChIP-chip data from the study of Boyer et al, [16] (Table S2) and found that of our 2282 c-Myc-bound promoters represented on the Boyer et al. array 9.5% are H3-K27 methylated and associated with the PcG protein Suz12 (p = 3.4E−03, Chi-square calculation). This represents 18% of Suz12 + H3-K27me3 genes and 17% of all genes displaying H3K27 trimethylation (p = 2.9E−03) (Figure 3B, Table S2). The overlapping Suz12/H3-K27me3/c-Myc genes predominantly encode transcription factors involved in development and differentiation (Figure 3C) such as Ebf2, MyoD1, Hand1, Isl1 and Atoh1, the homeobox gene family members Hox b,c,d, and genes involved in critical signaling pathways such as Wnt and FGF (for gene list see Table S2).

Our ChIP-chip analysis also revealed that among the group of PcG bound genes associated with c-Myc are many genes previously characterized as possessing bivalent chromatin marks [20], [21]. Furthermore when we examined a group of these known bivalent genes by qPCR we found that they are induced by c-*myc* overexpression and repressed following c-*myc* knockdown (e.g. Zic4, Ebf1 and Foxp2, Fgf5, Fgf18, Ebf2, Hand1, Olig2, Lhx2, HoxB3, Olig 3, Tcfap2b, En2, Atoh1, Ngn2, Ngn3, Gata6 marked by asterisks in Figure 2). These findings indicate that c-Myc binds to and can regulate PcG silenced genes in ES cells.

### c-Myc Influences the Level and Pattern of Histone H3 Lysine Methylation in ES Cells

**Figure 4:**
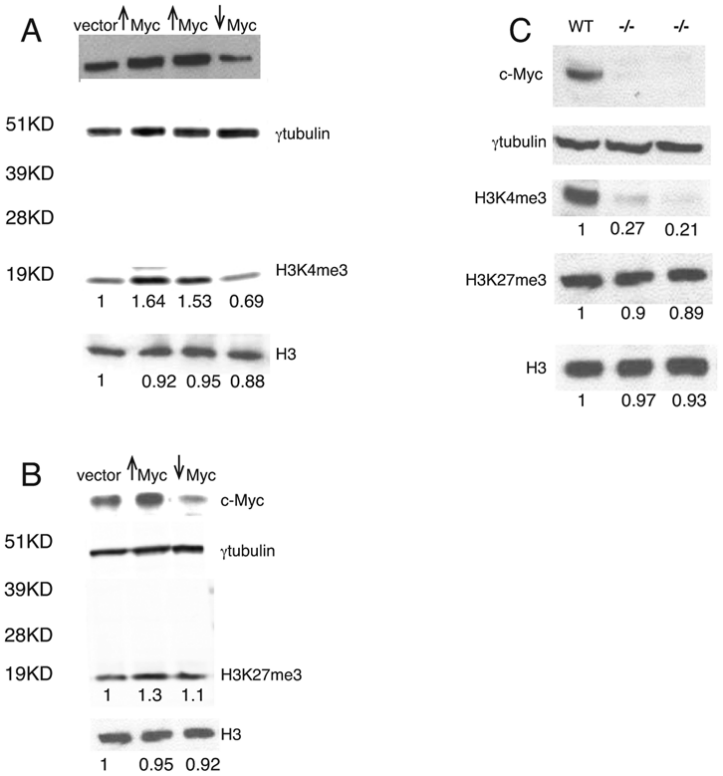
Quantitation of H3K4me3 and H3K27me3 levels in response to c-Myc. (A) Response of H3K4me3 to Lentiviral mediated overexpression or knock-down c-Myc in mES cells as determined by western blot. (B) Response of H3K27me3 to Lentiviral mediated overexpression or knock-down c-Myc in mES cells as determined by western blot. (C) Effect of H3K4me3 and H3K27me3 level in response to c-Myc loss in early developmental embryos. Ratios based on normalization to γ-tubulin internal control wwere calculated using ImageJ software .

Myc has been linked to histone methylation through its inhibitory interaction with the LID histone H3-K4me3 demethylase in Drosophila [63] and by Myc's apparent preference for binding to promoters that are methylated at H3-K4 [57], [65]. Above we have described Myc binding to PcG target genes that contain an H3-K27me3 mark (see Figure 3B). To determine if changes in c-Myc abundance affects the levels of H3-K4me3 and H3-K27me3, we carried out lentiviral-mediated Myc expression and determined the presence of these two histone marks in bulk chromatin using Western blotting. Introduction of the c-*myc* shRNA and overexpression vectors results in the expected changes in Myc protein levels (Figure 4A). Our results show that an approximately 8-fold increase c-Myc augments H3K4me3 abundance by 50–60% (Figure 4A). By contrast, H3K4me3 levels are decreased ∼30% subsequent to c-Myc knock-down (Figure 4A). When we examined H3-K27me3 under the same conditions we were unable to detect any significant change.

To determine whether loss of c-Myc expression has a significant effect on H3K4me3 level during embryonic development we examined embryos from wildtype and c-*myc* null mice. Targeted deletion of both c-*myc* alleles results in embryonic lethality at E10.5 [66] due to defective hematopoiesis [67]. When we analyzed c-*myc* null embryos at E7, a time when no phenotypic change is apparent between wildtype and c-*myc* null embryos, we found a dramatic (∼70–80%) decrease in H3K4me3 levels, but no significant change in H3K27me3 compared to wildtype. Our results suggests that c-Myc expression is involved in maintaining H3K4me3 levels in ES cells and in early embryonic development (Figure 4C).

**Figure 5:**
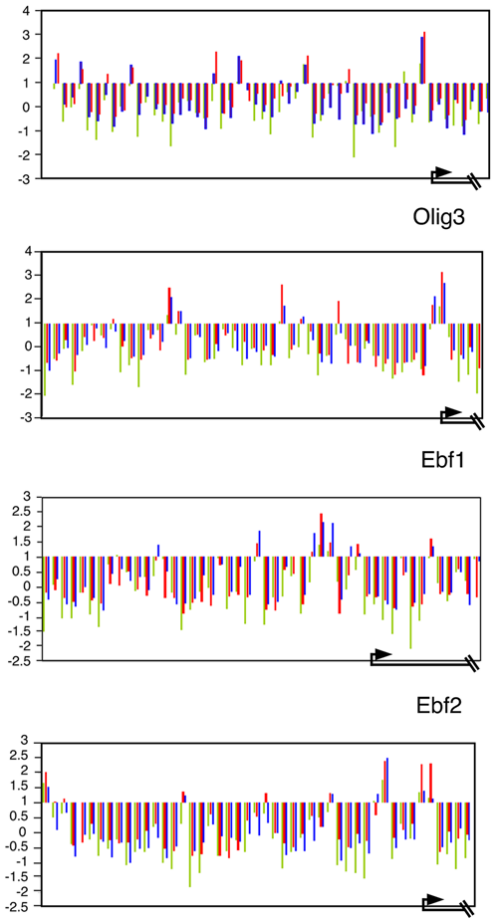
c-Myc binding sites on a subset of gene promoters in mES cells. c-Myc levels were manipulated by Lentiviral-delivered overexpression or knock-down of c-Myc in mES cells followed by anti-Myc ChIP-chip analysis on custom- designed arrays (see text). Unprocessed enrichment ratios (log2 scale) present ChIP-enriched versus total input genomic DNA (crosslinked, sonicated and processed identically to the ChIP sample) (y axis) for all probes within a genomic region 2 kb upstream and 1 kb downstream of the TSS. The TSS and direction of transcription are denoted by arrows at the bottom of the figure. Blue bars: endogenous c-Myc binding in WT mES cells, Red bars: Myc overexpressing mES cells, Green bars: Myc knock-down mES cells. Negative values occur when there is no enrichment in ChIP DNA relative to input.

To examine whether changes in Myc levels influence the H3 methylation landscape at individual Myc-bound genes in ES cells we extended our ChIP-chip analysis using antibodies against H3-K27me3 and H3-K4me3. We employed a custom-designed oligonucleotide tiling array of 815 promoter regions including promoters of genes that were bound by both Myc and Polycomb proteins as well as other Myc target genes and genes bound by Polycomb but not Myc, selecting genes that are predominantly linked to PcG binding, differentiation, pluripotency, and chromatin modification (Table S4). We first carried out anti-Myc chromatin immunoprecipitation on this custom array permitting us to verify our initial array data and examine the effects of modulating Myc levels. We compared vector-alone treated mES with mES cells overexpressing c-*myc* (mES ↑c-*myc*) and mES cells in which c-*myc* has been knocked down (mES ↓ c-*myc*). Figure 5 shows c-Myc binding to a representative group of promoters. We plotted log_2_ enrichment profiles to permit us to more easily compare wildtype ES cells with the mES ↓ c-*myc* and mES ↑c-*myc* cells. Here negative enrichment ratios indicate that the signal from the anti-Myc ChIP was less than the input DNA against which it was normalized. Ebf1, Ebf2, Olig3, Gata6, and Sox 2 display significant endogenous c-Myc binding at several sites within their promoters in mES cells (Figure 5 blue bars, Figure S10) consistent with our promoter array data (Table S1). Hes1, Onecut1, and Jak2 were not identified as positive for Myc binding on the original promoter array, and show relatively low levels of endogenous c-Myc binding in this high density tiling array (Figure S10). Overexpression of c-Myc generally results in increased enrichment (red bars) at endogenous binding sites, although in some promoters we observe overexpressed c-Myc binding at a small number of sites where no or very low endogenous binding signal is detected (e.g. Ebf2, Hes1, Olig3, Gata6) consistent with previously observed occupation of low affinity binding sites at higher Myc levels [27]. By contrast, shRNA-mediated knockdown of c-Myc invariably leads to reduced Myc binding (green bars). These findings further validate our Myc binding assignments and indicate that the extent of binding is dependent on c-Myc levels.

**Figure 6:**
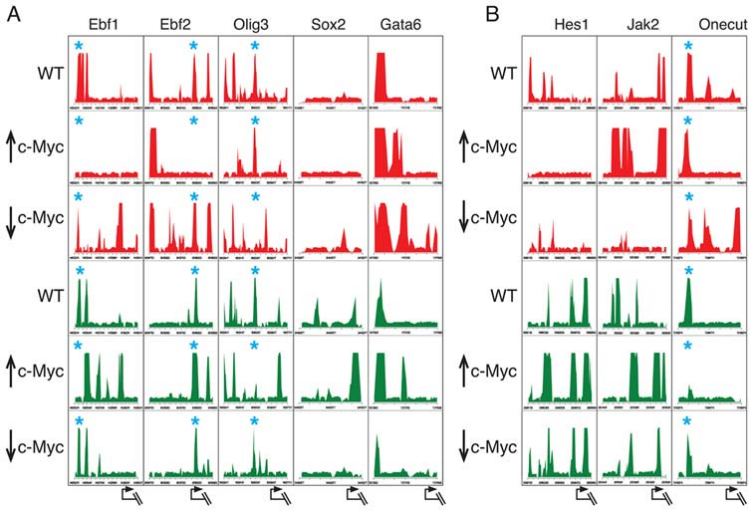
c-Myc levels influence the pattern of histone H3 lysine methylation in mES cells. (A) a group of genes bound by c-Myc (B) a group of non-Myc target genes. c-Myc levels were manipulated by (1) Lentiviral-delivered overexpression (↑c-Myc) (2) knock-down (↓ c-Myc), or (3) empty lentiviral vector control in ES cells. ChIP DNA isolated with anti-H3K4me3 or anti-H3-K27me3 was applied to the custom-designed arrays (see text). Enrichment ratios (log2 scale) for ChIP-enriched versus total input genomic DNA were processed by ACME and the given p values (−log10; y axis) identifying significant sites were plotted (see Methods). Red peaks present H3-K27me3 and green peaks present H3K4me3. Tick marks on the y-axis are spaced 400 bp apart. Asterisks mark positions of overlapping (bivalent) H3-K4me3 and H3-K27me3 peaks. The arrows at bottom indicate transcriptional start sites.

When we plotted histone H3 methylation profiles we found that nearly all promoters on our custom array (∼95%) displayed changes in the intensity and position of H3-K27me3 and/or H3-K4me3 peaks in response to altered Myc levels (profiles for 427 promoters are provided in Figures S5, S6, S7, S8, and S9). However the extent and type of change appear to be largely promoter-specific. Fig. 6 shows H3-K27me3 (red) and H3-K4me3 (green) in wildtype mES, mES↑c-*myc*, and mES↓c-*myc* cells over a 3KB to 5KB region proximal to the TSS of a subset of genes that are illustrative of the types of changes observed more generally (see Figures S5, S6, S7, S8, and S9). Sox2 is a c-Myc bound and regulated gene (Figure 1, 2, 6) that is not known to be a PcG target and, as expected, we fail to observe peaks of H3-K27me3 in the vicinity of the *sox2* promoter in mES or in mES ↑c-*myc* cells. However, H3-K4me3 is apparent in the *sox 2* promoter region in mES and shows, in mES↑c-*myc* cells, an increased signal near the *sox 2* TSS compared to wildtype mES cells (Figure 6A). The presence of an augmented H3-K4me3 peak is consistent with our finding that Sox2 is induced by c-*myc* overexpression (Figure 2A) (see Discussion). In contrast, c-*myc* knockdown abolishes H3-K4me3 and leads to repression of *sox2* (Figure 2B). Interestingly, a small peak of H3-K27me3 is present at the *sox2* promoter in mES↓c-*myc* cells, suggesting that loss of c-Myc binding may lead to PcG activity, at least within this promoter region (Figure 6A).

In ∼13% of the Myc targeted promoters on our custom array the overexpression of c-*myc* increased the levels of H3K4me3 and decreased H3K27me3 levels. Genes with enriched H3K4me3 in mES↑c-*myc* cells, such as Ebf1, Ebf2, and Sox2 (Figure 6A) encode transcription factors involved in early development. Their expression levels increase in mES↑c-*myc* and decrease in mES↓c-*myc* cells relative to wildtype mES cells (Figure 2). In addition, Ebf1, Ebf2 and Olig 3 were previously identified as bivalent genes [20] and indeed we observe that their H3-K4me3 and H3-K27me3 peaks overlap in wildtype mES cells (Figure 6 blue asterisks). For these promoters, augmentation of c-Myc levels results in an apparent resolution of the bivalency. Knockdown of c-Myc had little effect on H3-K4me3 distribution compared to wildtype mES cells although H3-K27me3 peaks are enhanced and clearly display changes in position. Loss of bivalency with c-*myc* overexpression was observed in ∼32% of genes on the custom array (Table S3).

A different pattern emerged from our analysis of another subset of promoters. Here, overexpression of c-Myc frequently results in enrichment of promoter proximal H3-K27me3. These include genes involved in differentiation such as MyoD1, mesenchyme homeobox 2 (Meox2), and Smarca2 (also known as Brahma, a component of the SWI/SNF related chromatin remodeling complex) or genes highly expressed in differentiated cells such as very low density lipoprotein receptor (VLDLR), glutamate receptor (Grm7), dopamine receptor D4 (Drd4), and the protocadherin cluster (see Table S3). Within the GATA6 promoter, Myc overexpression induced an H3-K4me3 peak as well as an overlapping H3-K27me3 peak, thereby creating a new bivalent region (Figure 6). GATA6 expression is repressed under these conditions (Figure 2A,B). Knockdown of c-Myc results in decreased H3-K4me3 in the presence of widespread H3-K27me3. Therefore induction of GATA6 expression upon c-Myc knockdown (Figure 2A,B) is presumably dependent on other changes in chromatin modification or remodeling independent of H3 methylation (Figure 2).

Analysis of a group of genes that did not score as Myc targets showed no or only small changes in methylation patterns (Figure 6B). However we find that some non-Myc targets such as Jak2, Hes1, and Onecut1 reveal changes in H3 methylation (Figure 6B). Jak2 shows an increase and spreading of H3-K27me3 as well as H3-K4me3 over the promoter region. mES↓c-*myc* cells display a nearly complete loss of the H3-K27me3 and a diminished intensity of H3-K4me3 in the Jak2 promoter region. Onecut1 and Hes1 also display changes in both H3-K27me3 and/or H3-K4me3 in response to changes in Myc levels. These effects are likely due to secondary or long-range effects of Myc on chromatin (see Discussion). Overall our data suggest that altering Myc levels induces varied and widespread changes in methylation patterns.

## Discussion

The recent findings that Myc family proteins can collaborate with other transcription factors (Oct4, Sox2, Klf4) to reprogram mouse and human fibroblasts [50], [51], [68]–[70] as well as earlier work showing the importance of c-Myc in maintenance of mES cell self-renewal [41] prompted us to examine the nature of c-Myc target genes in mES cells using ChIP-chip analysis. Previous studies employing ChIP-chip and Chip-PET in human cells and DamID in Drosophila cells indicated widespread binding by Myc [27]–[31]. Genomic loci associated with Myc binding were found to encompass a wide range of biological functions including metabolism, protein biosynthesis, and cell signaling – functions generally consistent with the ability of Myc proteins to stimulate cell growth. Our ChIP-chip analysis of Myc bound loci in mES cells has identified a similarly broad range of functional categories related to growth and metabolism among 3189 binding sites. However, among the mES cell c-Myc targets identified here, we also detected genes involved in developmental processes (e.g. Sox2, Ebf1, Atoh1, En1, Hand1, Bmi1, Suz12) many of which had not been previously connected to Myc. We also detected genes not previously identified as Myc targets that are involved in chromatin assembly and modification including HirA, HirIP5, SMCX, SMRCA2, Anp32b, and Anp32e. Examination of gene targets using qChIP-PCR and/or RT-PCR confirmed that many are bound and regulated by Myc. That Myc can elicit cell-type specific gene regulatory programs had been earlier suggested by a series of expression profiling experiments in pancreatic β-cells that uncovered a striking, and most likely direct, regulation of pancreatic cell-specific differentiation related genes by c-Myc [71]. Our data indicate that in mES cells Myc participates in binding and regulation of genes important in controlling aspects of chromatin structure and pluripotency. That Myc affects ES cell biology is consistent with an earlier study employing overexpression of mutant forms of Myc and demonstrated that c-Myc promoted mES cell self-renewal through inhibition of differentiation [41] as well as recent data demonstrating that Myc regulates ES cell differentiation through the induction of a specific subset of microRNAs [60].

While this work was in progress three reports were published describing Myc binding sites in ES cells [72]–[74]. Two of these studies focused on binding by multiple core transcription factors [72], [73] and the third examined Stat3 and Myc [74]. In these reports Myc binding was largely examined in the context of binding and gene regulation by these other factors. In this paper we have used cells sorted for the ES cell marker SSEA-1 to achieve a highly purified and phenotypically uniform population of ES cells. In addition we not only identified genes bound by endogenous c-Myc in ES cells but have examined the effects of Myc overexpression and knockdown on promoter occupancy and the landscape of H3-K4 and H3-K27 methylation. In general while our results agree with these reports in finding widespread binding of Myc to many genes critically involved in ES cell function, we have also found evidence implicating Myc in histone methylation and in bivalency in a subset of PcG target genes.

### Participation of Myc in ES Cell Transcription Circuitry

It is noteworthy that *sox2*, encoding a critical transcription factor regulating pluripotency, is regulated and bound by Myc. Moreover histone H3 methylation at the sox2 promoter is altered in response to changes in c-*myc* abundance. This result is consistent with a previous study demonstrating elevated Sox2, among other pluripotency factors, in an ES cell-like expression module discovered in diverse human epithelial cancers [76]. Recently several pluripotency genes have been shown to be regulated by N-Myc overexpression in neuroblastoma [75]. This raises the possibility that Myc may participate in the Sox2 pluripotency network by directly up regulating Sox 2. We also detected overlap between Myc targets and genes marked by H3-K27me3 and Suz12 binding. Other studies have reported only a low correlation between c-Myc targets and H3-K27me3 [65], [73]. Our data are consistent with this work in that we find only a fraction of Myc targets that overlap with PcG target genes- thus Myc in general does not associate with H3-K27 methylated gene regions. However the 218 genes that do display Myc, Suz12 and H3-K27me3 overlap include many transcription factors involved in developmental regulation of differentiation functions (predominantly ectoderm and neuronal differentiation). The genes encoding these transcription factors are repressed by PcG complexes (including the Suz12 subunit) in ES cells [16]–[18]. Importantly, of the 17 common Myc-PcG target genes tested we found that 14 (Fgf18, EBF1, EBF2, Zic4, En2, Bmi1, Lhx2, Fgf5, Ngn2, Ngn3, Olig 2, Olig3, Atoh1, and HoxB3) are activated upon Myc overexpression and repressed subsequent to Myc knockdown in mES cells (Figure 2A, B). Because many of these promoters marked by H3-K27me3 are considered bivalent [21]–[23] we surmise that Myc is involved in the maintenance of the bivalent state.

### Myc Influences the H3 Lysine Methylation Landscape

While there is a compelling connection between Myc binding and the induction of histone acetylation [57], [65], [77] it is less clear how Myc influences histone methylation. In a human B cell line Myc binding is strongly associated with promoters marked by H3-K4me3 prior to the induction of Myc [65]. In Drosophila dMyc binds to and inhibits the activity of the H3-K4me3 demethylase Lid, [63] suggesting that at least in this system Myc may act to maintain H3-K4 methylation levels by blocking the action of a demethylase [63]. When we analyzed c-Myc regulation of H3-K4 and H3-K27 methylation in bulk chromatin derived from ES cells we found that H3-K3me3 levels positively correlate with c-Myc levels while H3-K27me3 levels were unaffected by c-Myc abundance (Figure 4A, B). Surprisingly, phenotypically normal E7 embryos from c-*myc* null mice (approximately 3 days prior to the advent of embryonic lethality) have strikingly reduced H3-K4me3, while H3-K27me3 was essentially unchanged (Figure 4C). This result extends our earlier work demonstrating global decreases in H3-K9 methylation and H3/H4 acetylation in bulk chromatin in the nervous system and in cell lines upon Myc loss of function [56].

To examine the effects of c-Myc levels on individual genes we analyzed H3-K4 and H3-K27 trimethylation within the subset of Myc associated genes linked to PcG binding, differentiation, pluripotency, and chromatin modification. Our results show broad and complex changes in the trimethylation patterns of both H3-K27 and H3-K4 in nearly all the promoters on the array in response to altered Myc levels. These changes display several noteworthy features. First, H3 methylation changes in response to altered Myc levels are generally, but not exclusively, observed in promoter regions directly bound by Myc. However, while Myc binding sites are frequently proximal to altered H3 methylation sites, peaks of Myc binding can also be found several kb away. Second, the patterns of H3 methylation changes observed appear to be promoter specific. A recent study in human B cells also reported that induction of Myc resulted in histone methylation changes at individual promoters at both Myc and non-Myc target genes [57]. Here we find that methylation changes can take the form of shifts in position within the promoter and/or quantitative changes that presumably reflect changes in the fraction of mES cells containing the altered methylation mark. Third, H3-K27 and K3-K4 methylation bivalency is both lost and gained depending on the specific promoter and the levels of Myc. It is important to note that while the changes observed in H3-K4 and H3-K27 trimethylation patterns can be construed as consistent with activation or repression in response to changes in c-Myc levels, we have also observed cases where altered methylation patterns do not correspond to changes in gene expression. This is consistent with a previous study demonstrating that during differentiation active PcG recruitment to some genes can result in increased H3K27me3 but not to inhibition of transcription [25]. H3-K4 and H3-K27 methylation patterns represent only a part of the complex interplay of modifications and factors involved in chromatin structure and gene regulation [73], [78] underscoring the importance of more detailed analysis of other histone marks and transcription factor interactions at individual promoters.

### Mechanisms of Epigenetic Regulation Mediated by Myc

How does Myc elicit changes in the histone H3 methylation patterns of promoters in mES cells? A great deal of previous work has indicated that the Myc protein recruits multiple activities involved in chromatin modification – these include histone acetyltransferases (GCN5, Tip60, p300), potential chromatin remodelers (p400, Ini1, Tip48/49), ubiquitin ligases (Fbw7, Skp2), the H3-specific kinase Pim1, the HDAC1 histone deacetylase, and the JARID1A/LID histone H3-K4 demethylase (for reviews see [38], [61], [79]). Furthermore, studies in Drosophila indicate that dMyc interacts with members of the Trithorax group, including components of histone methyltransferases and Swi/Snf remodeling complexes [63], [64]. It is likely that the precise mix of co-regulators recruited by Myc to DNA is determined in a context-specific manner and, given the short half-life of Myc proteins, is subject to rapid exchange. The consequences of these multiple interactions are predicted to have profound effects on chromatin proximal to Myc binding sites and may in principle lead to erasure or establishment of H3 methylation patterns. Such modification patterns may be spread along chromatin, as has been suggested for H3-K9 and H3-K27 methylation and heterochromatin formation [80]. These effects may be reinforced by the nature of the target genes induced by Myc which include chromatin modification enzymes (GCN5, SMCX) and remodelers (SMARC). Moreover, recent work has shown that nucleosome replacement mediated by factors controlling assembly and disassembly may underlie epigenetic changes that govern accessibility of transcription factor to promoters (for review see [8]). In this regard it is of interest that the gene encoding the histone assembly factor HirA is bound and regulated by Myc. HirA levels have been previously shown to regulate unbound histones and ES cell differentiation [1]. Therefore Myc may stimulate a more generalized nucleosome instability leading to widespread changes in epigenetic marks. Such generalized effects of Myc -through spreading of chromatin modifications, through induction of Myc target genes which themselves modify chromatin, and through nucleosome instability – may account for the fact that we detect changes in histone methylation patterns in regions distant from Myc binding sites and in promoters in which Myc is not bound. We surmise that the ability of Myc to broadly affect the epigenetic marks and expression of genes important in pluripotency and differentiation is related for Myc's role as a reprogramming factor critical in the generation of iPS cells.

## Materials and Methods

### Cell Culture

The ES cell lines, R1 and AK7, the most commonly used cell lines for generating transgenic mice, were obtained from Andras Nagy and Philippe Soriano respectively. Both mES cell lines were cultured in DMEM supplied with 10% FBS, L-glutamine, non-essential amino acid, sodium pyruvate, LIF (leukemia inhibitory factor), and β-mercaptoethanol. ES cells were thawed on plates with a mitomycin C-treated mouse embryonic fibroblast feeder layer and then grown and expanded on 0.1% gelatin-coated plates (without feeder cells) in medium containing LIF for subsequent experiments.

### c-Myc Constitutive Knock-Out Mice

The c-Myc constitutive knock-out mice were generated by crossing more-Cre and c-Myc ^floxed/floxed^ mice (a gift of I.M. de Alborán) to delete the floxed c-Myc allele and then back-crossing to wild-type to remove more-Cre. The resulting c-Myc heterozygous mice were bred to obtain the c-Myc null embryos for further experiments.

### Ethics Statement

All research involving mice has been conducted according to the Institutional Animal Care and Use Committee Guidelines and has been pre-approved by the Institutional Review Board at the Fred Hutchinson Cancer Research Center.

### Chromatin Immunoprecipitation (ChIP) and DNA Microarrays (ChIP-chip)

ES cells were purified by SSEA-1 flow sorting and treated with 1.1% formaldehyde for crosslinking which was terminated with 0.125M glycine before lysis and sonication to produce 0.2∼1kb DNA fragments. Protein-A- agarose and protein-G-sepharose beads were blocked with 1 µg/ml sonicated salmon sperm DNA and 0.5 mg/ml BSA and washed twice in lysis/sonication buffer (50 mM Tris, 10 mM EDTA, 1%SDS, pH = 8). For ChIP with mouse monoclonal or rabbit polyclonal antibody, chromatin was pre-cleared with appropriate beads before immunoprecipitation overnight with antibody. Antibodies used for chromatin immunoprecipitation were c-Myc (Santa Cruz Biotechnology Inc. #SC764, Lot:F1307; Ab5 mouse monoclonal antibody from Thermo Scientific #MS-1054), H3K4me3 (Upstate, #05-745), and H3K27me3 (Upstate, #07-449), EZH2 (Abcam, #Ab3748-100), and Suz12 (Upstate #07-379). The N262 antibody antibody has been used extensively for ChIP of c-Myc [27], [31], [81]. We further pre-absorbed the N262 antibody with electrophoretically fractionated proteins extracted from c-Myc null embryos and demonstrated that the pre-absorbed antibody is specific for c-Myc protein and does not cross-react with N-Myc as determined by IP-western blotting.

Chromatin immunoprecipitation and amplification (ligation-mediated PCR) for ChIP-chip analysis were carried out according to the Roche/NimbleGen protocol. Input DNA represents an aliquot of chromatin from the same sample used for ChIP and was crosslinked, sonicated, and processed identically to the ChIP sample. Duplicate samples were applied to the arrays. Our initial mouse promoter DNA microarray comprised 21,632 putative promoters from annotated mouse genes (NimbleGen, two mm8 promoter arrays). Using R software (http://www.R-project.org), we merged normalized data with the corresponding gene identifiers. Genes bound by Myc were identified as those in which any probe in the 5 kb promoter segment had a log_2_-ratio (ChIP vs. input DNA) >2. Data was further analyzed by the ACME package (www.bioconductor.org), a computer program developed for analysis of data obtained from NimbleGen-tiled microarrays, using a window of 500 bases and a threshold  = 0.95 [58] [R Development Core Team (2008). R: A language and environment for statistical computing. R Foundation for Statistical Computing, Vienna, Austria. ISBN 3-900051-07-0].

To validate the ChIP-chip data from the promoter array, 1 ng input and ChIP DNA for Real-Time PCR was carried out using SYBR green PCR mix (Applied Biosystems) on ABI7900HT detection system. The Ab5, c-Myc specific antibody was used for qChIP-PCR validation. The enrichment ratio was calculated by ChIP versus input and the positive targets were determined by criteria of 2-fold enrichment. We evaluated enrichment from duplicates of 2 independent ChIP assays obtained from both the R1 and AK7 ES cell lines. The sequences for all primers for ChIP-PCR are available upon request.

Custom-design DNA microarrays for a subset of genes (see text) were tiled through >5 kb upstream and 1 kb downstream of transcription start sites using isothermal probes of 70–80 nucleotides. Triplicate samples were applied to the array. Genomic sites enriched for H3-K4me3 or H3-K27me3 binding were identified using ACME (window = 200, thresh = 0.9) and the given p values for each probes from ACME were plotted into graphs using the SAS 9.1 program.

### Analysis of Myc Target Genes

To determine overlap among genes bound by c-Myc in mES cells (this paper) as well as by PcG and H3-K27me3 in mES cells we merged the list of genes on our arrays with those on the platform used by Boyer, *et. al*. [16].The majority of PcG bound sequences were detected within 1 kb of transcription start site [16]. Genes not included in both datasets were excluded from the analysis. Myc binding promoter –associated genes were annotated using Panther software (http://www.pantherdb.org/) [82]. Genes that are not assigned to a biological process (biological process unclassified) were not included in the figures. Each list is compared to the reference list (NCBI:M. Musculus genes) using the binomial test for each biological process term.

### Manipulating c-Myc Level in ES Cells by Lentiviral-Delivered Overexpression or shRNA Knock-Down

c-Myc cDNA was amplified from ES-derived cDNA library and cloned into lentiviral vector pLenti6/V5-DEST (Invitrogen) for overexpression. The shRNA sequences against c-Myc was designed by using Dharmacon siDESIGN and then hairpin shRNA oligonucleotides were annealed and cloned into modified FUGW lenti-vector with H1 promoter (modified by Dr. Valera Vasioukhin). Lentiviral production was followed the protocol described in Lois C. et. al., Science, 2002, then drug selection for positive integration in ES cells followed by 3 days infection. Positive clones were picked and expanded for custom ChIP-chip array, qChIP-PCR, gene expression analysis by RT-PCR, and differentiation subsequent to LIF withdraw.

### Gene Expression Analysis by RT-PCR

Total RNA was extracted by TRIzol (Invitrogen) reagent and cDNA was synthesized by SuperScript II kit (Invitrogen) according to the manufacturer's instructions. The transcript expression level was measured by Real-Time PCR with SYBR Green detection on ABI7900HT system (Applied Biosystems). S16 RNA was used as internal control for normalization (ΔCt). The expression level was measured by subtraction of ΔCt from either overexpression or knock-down to vector control (ΔΔCt). Primers for Real-Time PCR are available upon request.

### Proliferation and Growth Rate Assay FACS Analysis

MTT assays: ES cells were seeded 1000 cells per well of 0.1% gelatin-coated 96-well plate supplied with LIF and analyzed 24, 48, 72, 96 hours after plating. At each time point, ES cell medium was replaced with 100 µl 1 mg/ml MTT (Molecular Probe) for labeling. Subsequently, 100 µl of SDS-HCl was added to each well and mixed thoroughly after 3 hours labeling at 37°C. Following 4 hours incubation at 37°C in a humidified chamber, absorbance was recorded at 570 nm. Triplicate analysis was applied to each time point.

Population doubling time assay: 10^5^ cells was plated on 1 well of 6-well plate and counted by SSEA-1 positive population after 24 and 48 hours later. Briefly, ES cells were harvested by trypsin and washed with PBS prior to SSEA-1 (mouse monoclonal 1∶200 dilution) antibody incubation for 2 hours. After washing with PBS twice, cells were incubated with FITC-conjugated secondary antibody for 1 hour. Finally, cells were washed twice and resuspended in PBS before FACS analysis. The population (%) of positive SSEA-1 stained cells was compared to unstained cells, FITC-isotype control, undifferentiated ES cells, and cells stained with secondary antibody alone. The SSEA-1 positive cell numbers were subjected to the calculation of ”cell population doubling time“ with the equation Y_end_ = Y_start_×2 ^(t/T)^.

## Supporting Information

Figure S1 Identification of Myc binding sites in ES cells. In addition to the log2 ratio >2 we also applied the widely used ACME/R anaylsis which employs a sliding window = 500 b.p. and a threshold = 95% across gene promoters to determine significance (p-value) of each probe. Our identification of Myc binding sites is based on significant peaks (ACME: p-value <0.0001) that take into account binding events on neighboring probes. Shown are a few typical promoters in which are plotted log2 (NimbleGen enrichment ratio scale on left ; probes  = green dots) and -log10 (ACME p value in red. Scale is shown on right of each figure).
(0.15 MB PDF)

Figure S2 Validation of anti-Myc ChIP-chip results in the R1 mES cell line We randomly selected 44 genes for validation and confirmed the enrichment of 44 genes on the array by qChIP-PCR. Known Myc target genes such as Bmi1 served as a positive control. Genes Evx2 and Sox21 not identified in our ChIP-chip analysis and not previously reported as c-Myc targets were used as negative controls for validation by qChIP-PCR. Bar heights represent the mean “Fold” enrichment from 2 independent sets of anti-Myc ChIP-enriched versus total input genomic DNA. Six genes were not consistent in both our ChIP-chip assay and qChIP-PCR. From this we calculate the average “false discovery rate” in R1 ES cells to be 11.3%.
(0.01 MB PDF)

Figure S3 Determination of ES cells proliferation rate in response to c-Myc level. c-Myc levels were manipulated by Lentiviral-delivered overexpression or shRNA knock-down of c-Myc in ES cells. (A) BrdU labeling of a representative ES colonies. (B) Mean growth rate was measured by MTT assay. Mean was measured from triplicate experiments. (C) Mean population doubling time obtained from 4 independent experiments.
(0.03 MB PDF)

Figure S4 Sox2 and HirA protein expression in mES cells. Antibodies against Sox2 and HirA were used for immunoblots prepared from mES cells infected with empty lentiviral vector alone (WT; left panel); with a lentiviral vector expression shRNA against c-myc (middle panel); or with a lentiviral vector expressing c-myc (right panel). γ-tubulin was used for loading control.
(0.04 MB PDF)

Figure S5 H3-K27me3 (red) and H3-K4me3 (green) patterns at promoters of genes activated following c-Myc overexpression. DNA immunprecipitated with anti-H3-K4me3 or anti-H3-K27me3 was applied to the custom-designed array (see text). Enrichment ratios (log2 scale) for ChIP-enriched versus total input genomic DNA for 427 genes were processed by ACME and assigned p-values (-log10; y axis) identifying significant sites were plotted (see Methods). Red peaks present H3-K27me3 and green peaks present H3-K4me3.
(1.49 MB PDF)

Figure S6 H3-K27me3 (red) and H3-K4me3 (green) patterns at promoters of genes repressed following c-Myc overexpression. DNA immunprecipitated with anti-H3-K4me3 or anti-H3-K27me3 was applied to the custom-designed array (see text). Enrichment ratios (log2 scale) for ChIP-enriched versus total input genomic DNA for 427 genes were processed by ACME and assigned p-values (-log10; y axis) identifying significant sites were plotted (see Methods). Red peaks present H3-K27me3 and green peaks present H3-K4me3.
(1.14 MB PDF)

Figure S7 H3-K27me3 (red) and H3-K4me3 (green) patterns at promoters in which bivalency is partly lost upon c-Myc overexpression. DNA immunprecipitated with anti-H3-K4me3 or anti-H3-K27me3 was applied to the custom-designed array (see text). Enrichment ratios (log2 scale) for ChIP-enriched versus total input genomic DNA for 427 genes were processed by ACME and assigned p-values (-log10; y axis) identifying significant sites were plotted (see Methods). Red peaks present H3-K27me3 and green peaks present H3-K4me3.
(4.21 MB PDF)

Figure S8 H3-K27me3 (red) and H3-K4me3 (green) patterns at promoters in which bivalency is gained upon c-Myc overexpression. DNA immunprecipitated with anti-H3-K4me3 or anti-H3-K27me3 was applied to the custom-designed array (see text). Enrichment ratios (log2 scale) for ChIP-enriched versus total input genomic DNA for 427 genes were processed by ACME and assigned p-values (−log10; y axis) identifying significant sites were plotted (see Methods). Red peaks present H3-K27me3 and green peaks present H3-K4me3.
(1.11 MB PDF)

Figure S9 H3-K27me3 (red) and H3-K4me3 (green) patterns at promoters showing only small or no changes in H3-K27me3 and H3-K4me 3 pattern. DNA immunprecipitated with anti-H3-K4me3 or anti-H3-K27me3 was applied to the custom-designed array (see text). Enrichment ratios (log2 scale) for ChIP-enriched versus total input genomic DNA for 427 genes were processed by ACME and assigned p-values (−log10; y axis) identifying significant sites were plotted (see Methods). Red peaks present H3-K27me3 and green peaks present H3-K4me3.
(5.05 MB PDF)

Figure S10 c-Myc binding sites on a subset of gene promoters in mES cells. c-Myc levels were manipulated by Lentiviral-delivered overexpression or knock-down of c-Myc in mES cells followed by anti-Myc ChIP-chip analysis on custom- designed arrays (see text). Unprocessed enrichment ratios (log2 scale) present ChIP-enriched versus total input genomic DNA (crosslinked, sonicated and processed identically to the ChIP sample) (y axis) for all probes within a genomic region 2kb upstream and 1kb downstream of the TSS. The TSS and direction of transcription are denoted by arrows at the bottom of the figure. Blue bars: endogenous c-Myc binding in WT mES cells, Red bars: Myc overexpressing mES cells, Green bars: Myc knock-down mES cells. Negative values occur when there is no enrichment in ChIP DNA relative to input.
(0.16 MB PDF)

Table S1 List of Myc associated promoters in AK7 ES cells.
(0.58 MB XLS)

Table S2 Myc associated promoters showing H3-K27me3 Myc associated promoters showing Suz12 binding Myc associated promoters showing Suz12 binding and H3-K27me3 Note: data on Suz12 and H3-K27me3 from Boyer et al. 2006 (ref 16).
(0.15 MB XLS)

Table S3 List of genes organized into groups according to whether they are activated or repressed by c-Myc overexpression; whether they lose or gain bivalent H3-methylation marks; or show little or no change in methylation pattern. The individual pattern for each gene on the list is presented in Figs. S5–S9.
(0.02 MB XLS)

Table S4 List of genes included in custom-design microarray
(0.50 MB XLS)
