# Supplementary figures and images for: Gene Regulation and Epigenetic Remodeling in Murine Embryonic Stem Cells by c-Myc
Source: PLoS One. 2009 Nov 13;4(11):e7839. doi: 10.1371/journal.pone.0007839 (PMC2773118; doi:10.1371/journal.pone.0007839)

## Lin\_Supplemental FigS1

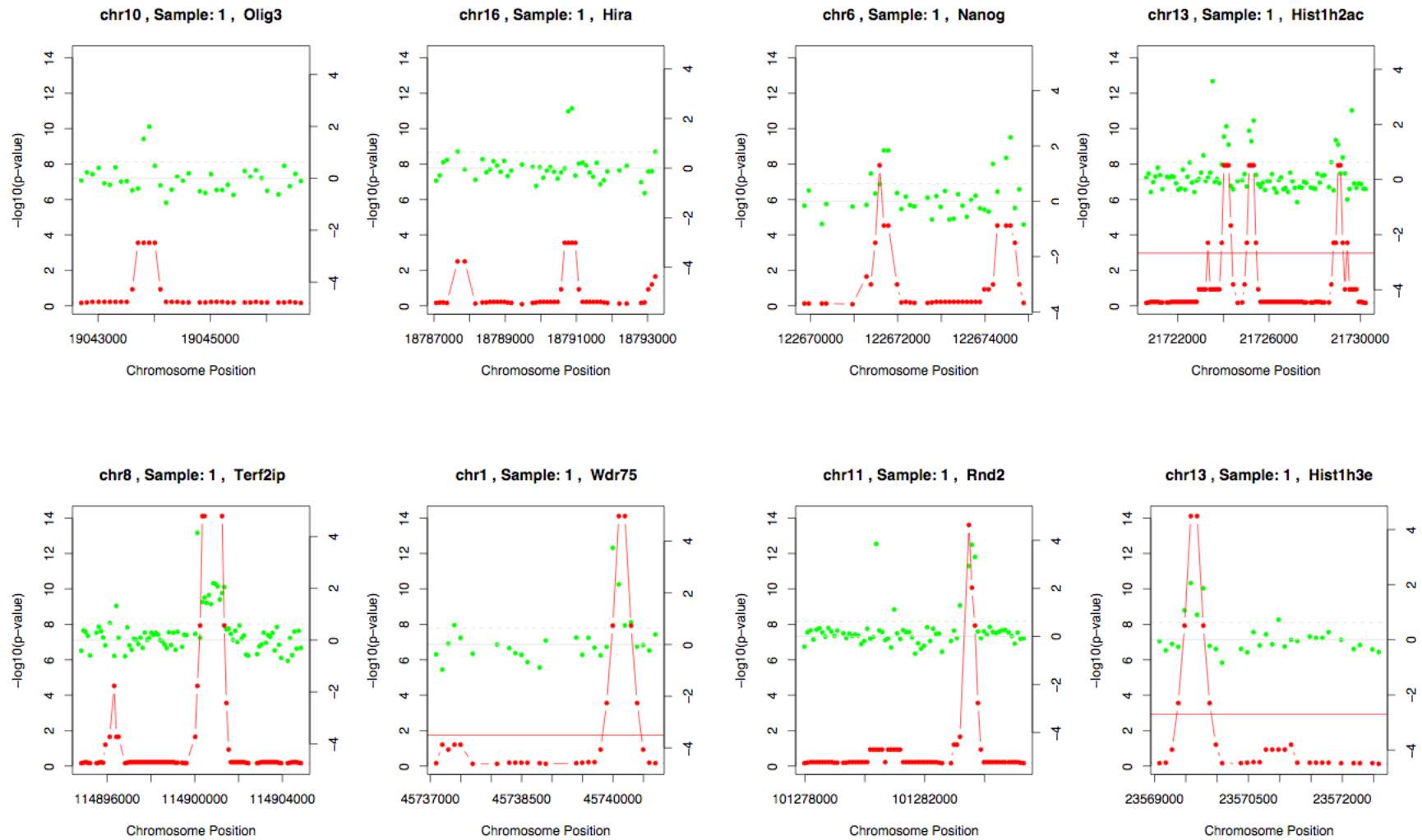

Supplement: Figure S1 — Identification of Myc binding sites in ES cells. In addition to the log2 ratio >2 we also applied the widely used ACME/R anaylsis which employs a sliding window = 500 b.p. and a threshold = 95% across gene promoters to determine significance (p-value) of each probe. Our identification of Myc binding sites is based on significant peaks (ACME: p-value <0.0001) that take into account binding events on neighboring probes. Shown are a few typical promoters in which are plotted log2 (NimbleGen enrichment ratio scale on left ; probes = green dots) and -log10 (ACME p value in red. Scale is shown on right of each figure). (0.15 MB PDF) [file pone.0007839.s001.pdf]

## Lin\_Supplemental FigS2

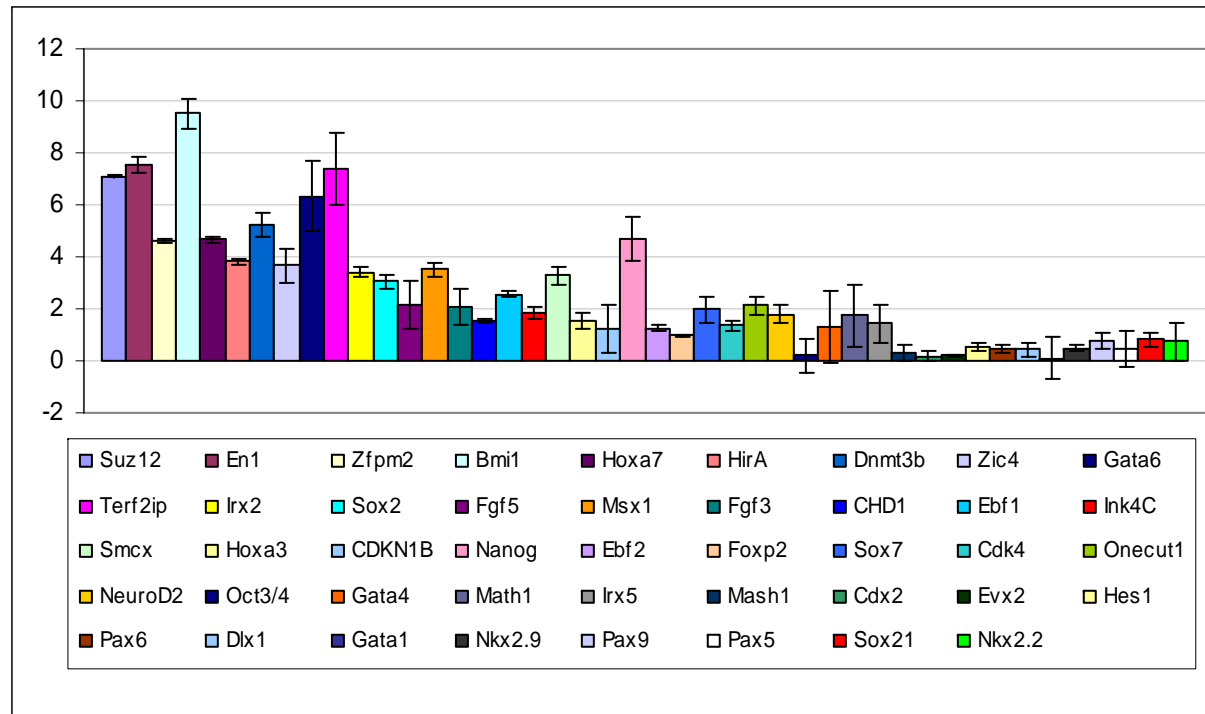

Supplement: Figure S2 — Validation of anti-Myc ChIP-chip results in the R1 mES cell line We randomly selected 44 genes for validation and confirmed the enrichment of 44 genes on the array by qChIP-PCR. Known Myc target genes such as Bmi1 served as a positive control. Genes Evx2 and Sox21 not identified in our ChIP-chip analysis and not previously reported as c-Myc targets were used as negative controls for validation by qChIP-PCR. Bar heights represent the mean “Fold” enrichment from 2 independent sets of anti-Myc ChIP-enriched versus total input genomic DNA. Six genes were not consistent in both our ChIP-chip assay and qChIP-PCR. From this we calculate the average “false discovery rate” in R1 ES cells to be 11.3%. (0.01 MB PDF) [file pone.0007839.s002.pdf]

Lin\_Supplemental FigS3

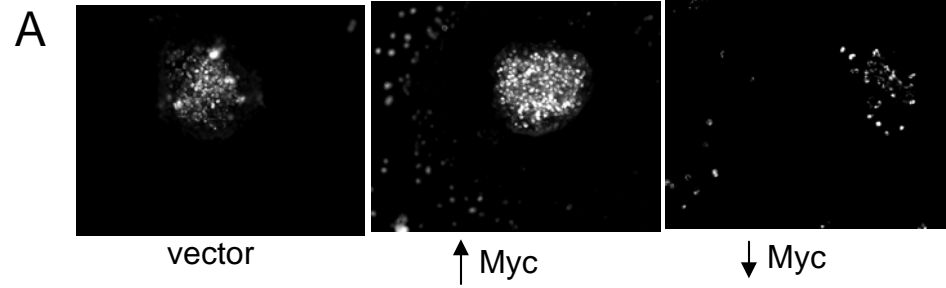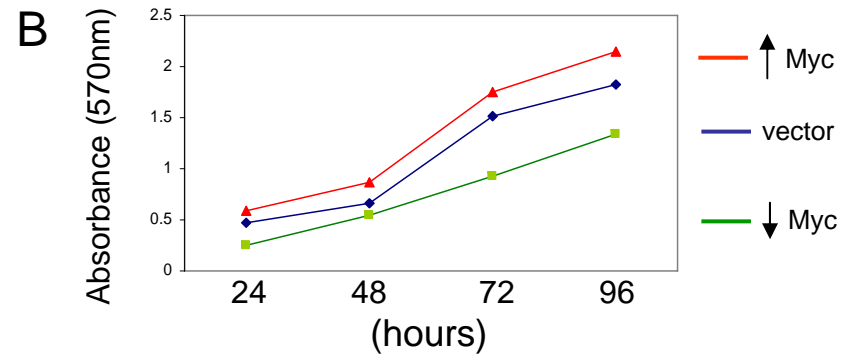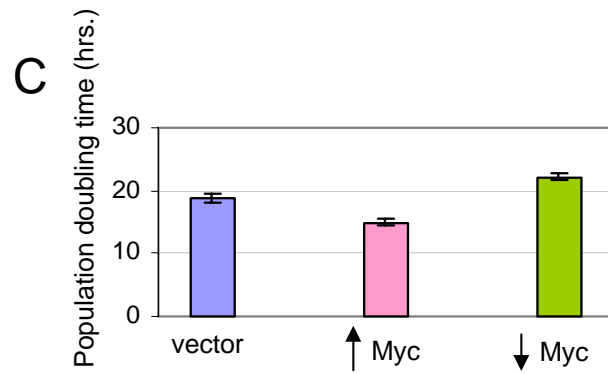

Supplement: Figure S3 — Determination of ES cells proliferation rate in response to c-Myc level. c-Myc levels were manipulated by Lentiviral-delivered overexpression or shRNA knock-down of c-Myc in ES cells. (A) BrdU labeling of a representative ES colonies. (B) Mean growth rate was measured by MTT assay. Mean was measured from triplicate experiments. (C) Mean population doubling time obtained from 4 independent experiments. (0.03 MB PDF) [file pone.0007839.s003.pdf]

## Lin\_Supplemental FigS4

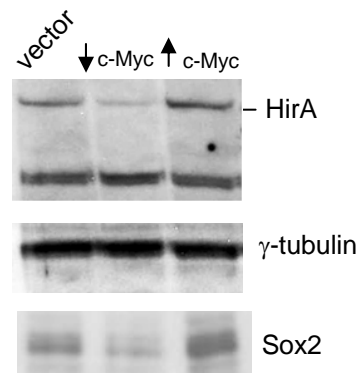

Supplement: Figure S4 — Sox2 and HirA protein expression in mES cells. Antibodies against Sox2 and HirA were used for immunoblots prepared from mES cells infected with empty lentiviral vector alone (WT; left panel); with a lentiviral vector expression shRNA against c-myc (middle panel); or with a lentiviral vector expressing c-myc (right panel). γ-tubulin was used for loading control. (0.04 MB PDF) [file pone.0007839.s004.pdf]

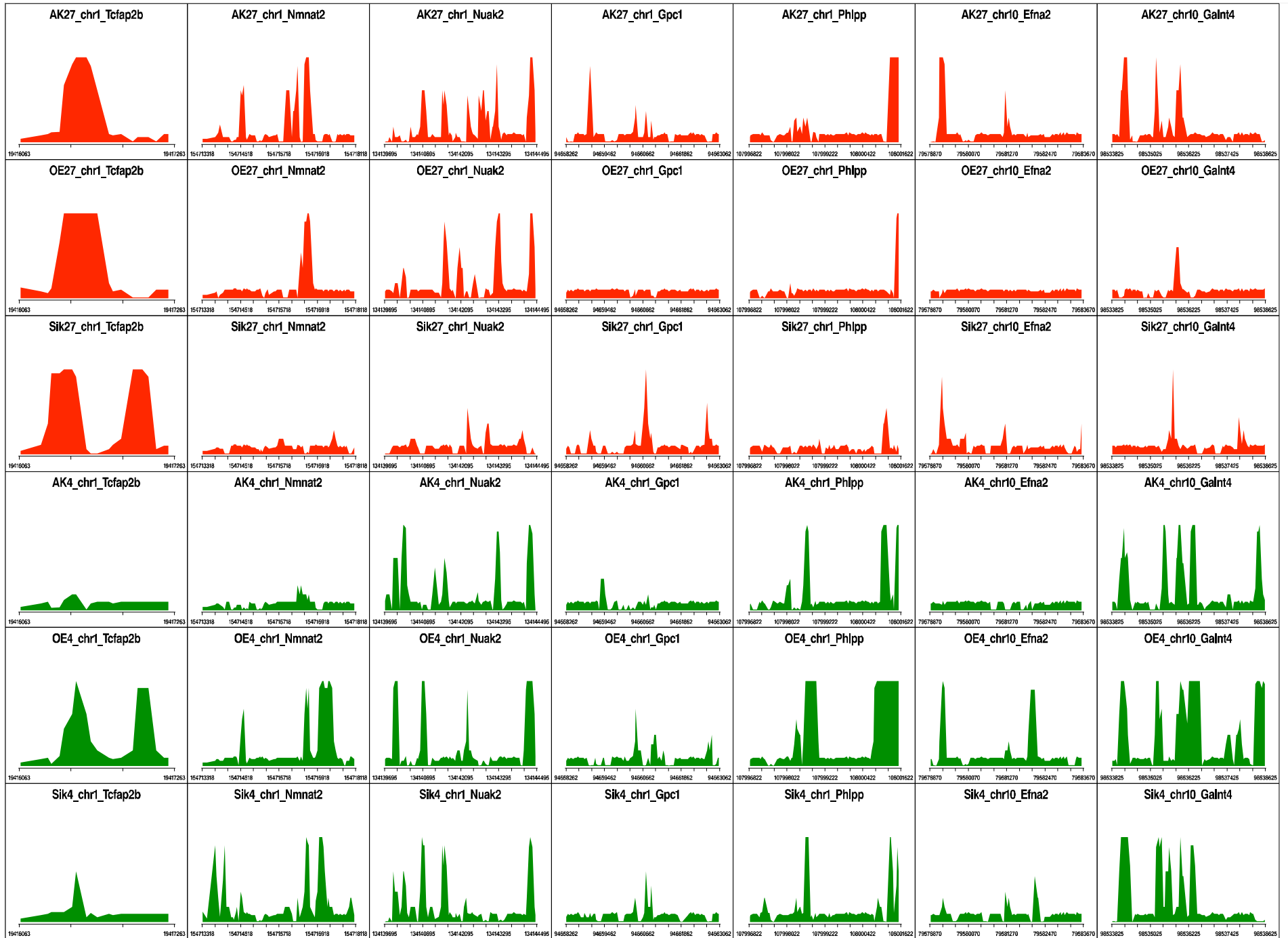

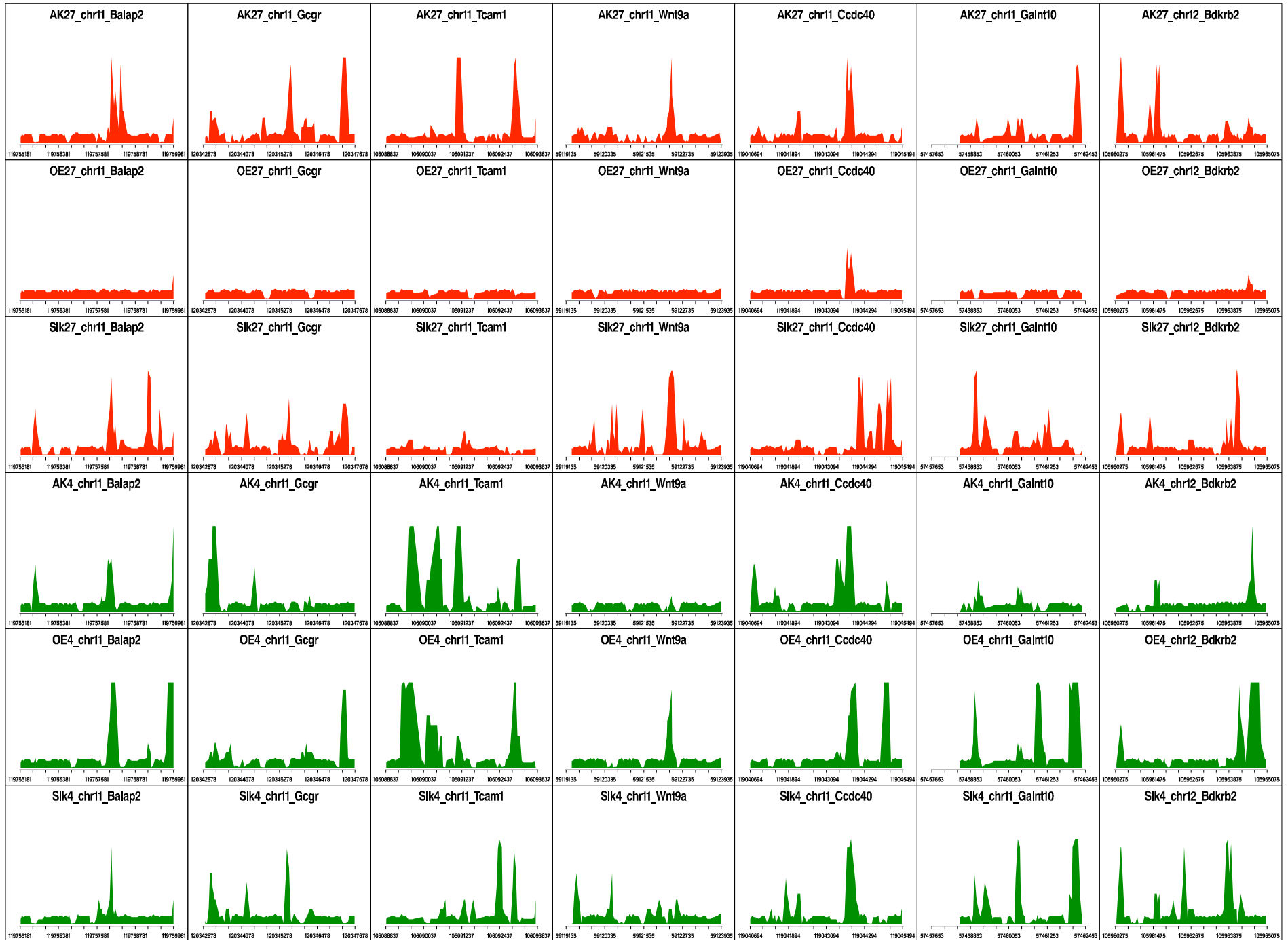

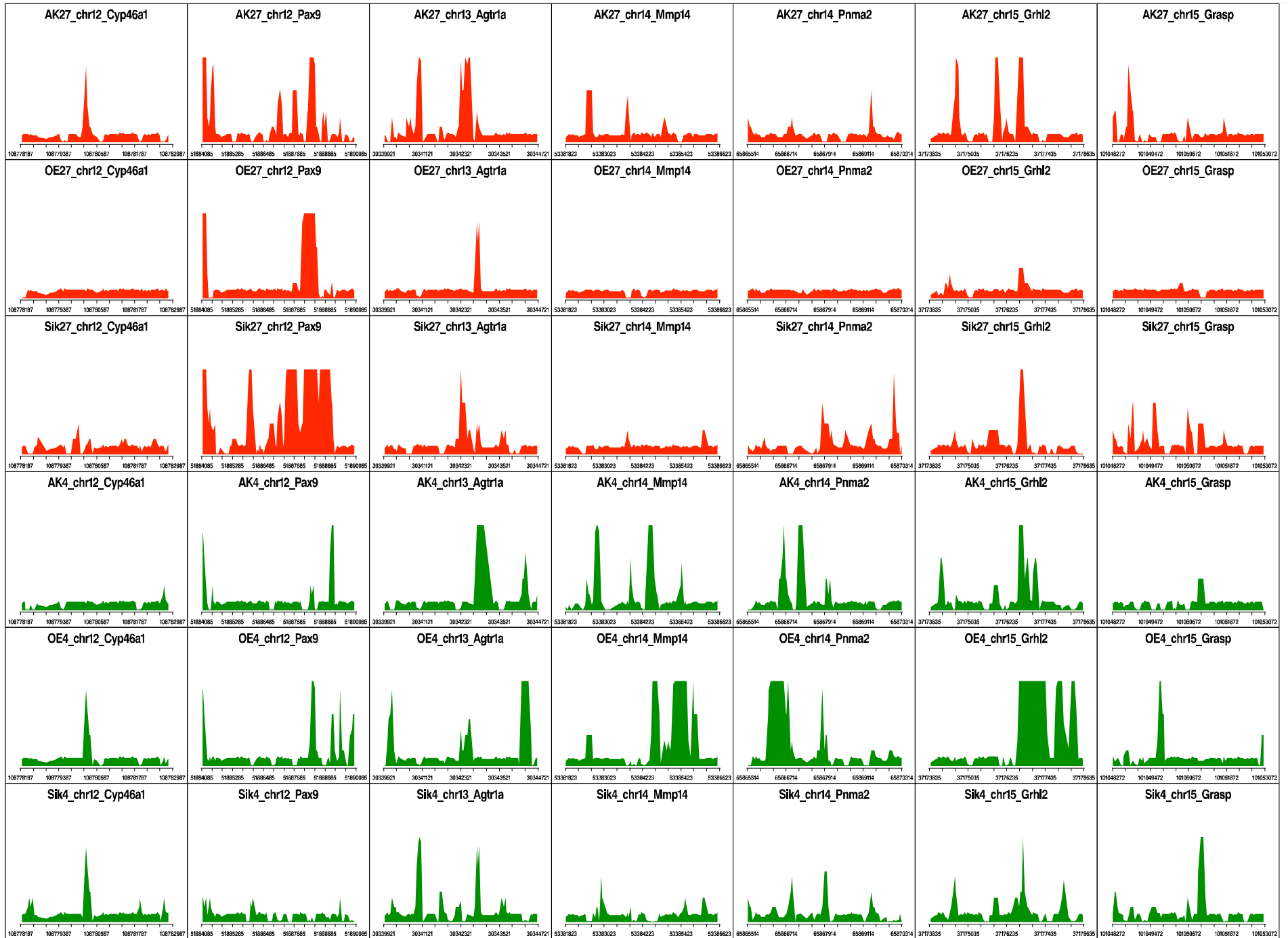

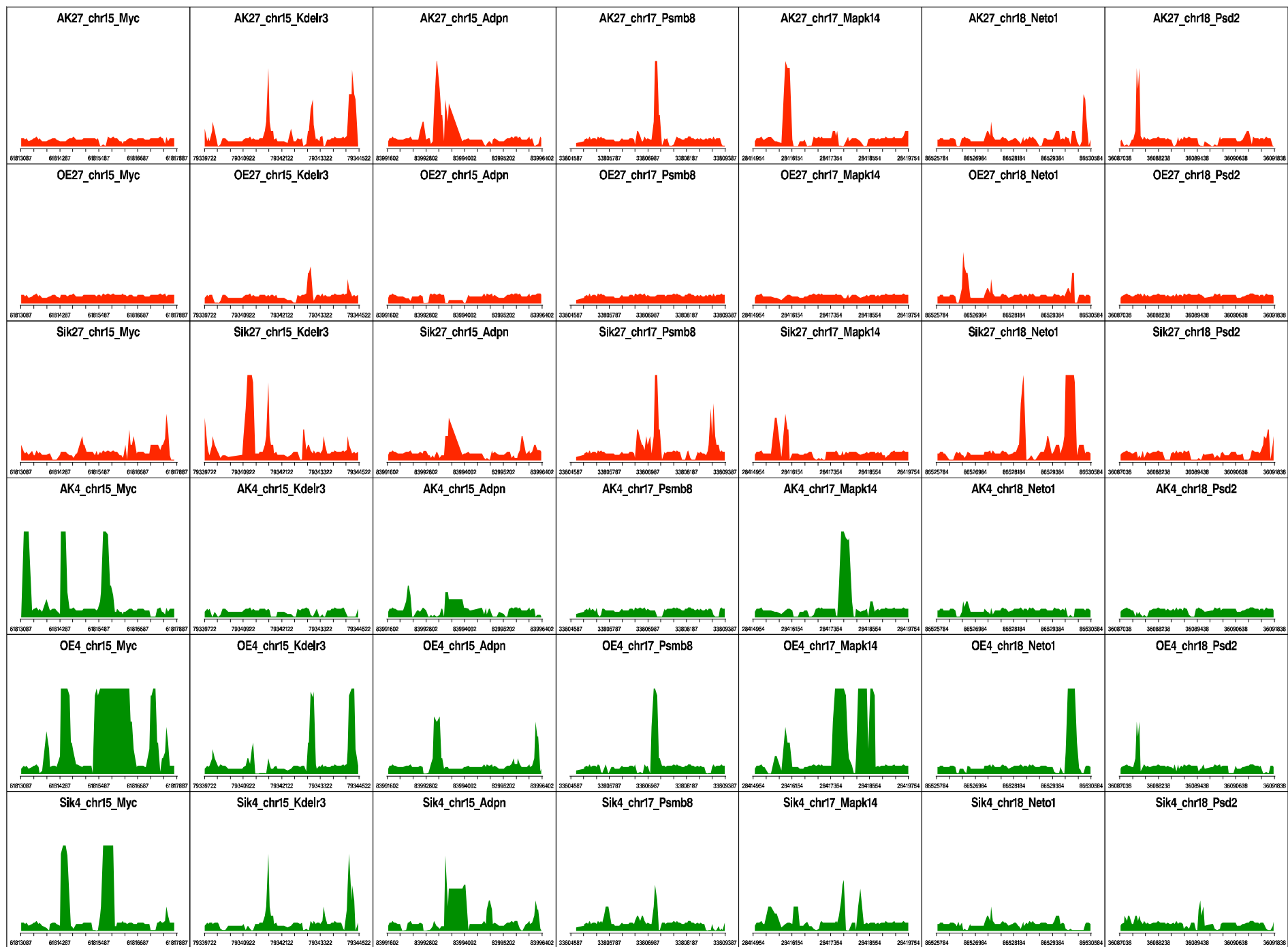

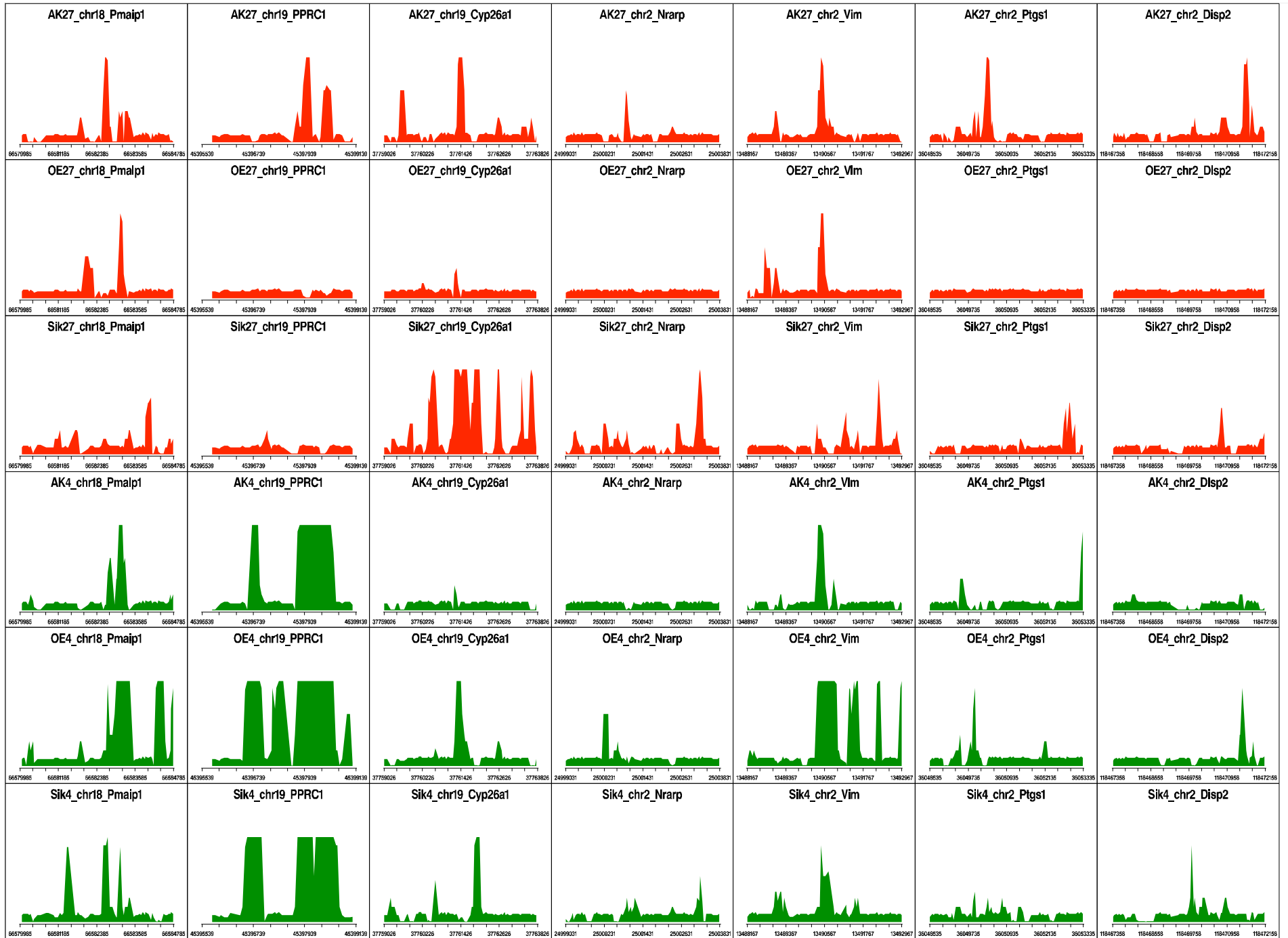

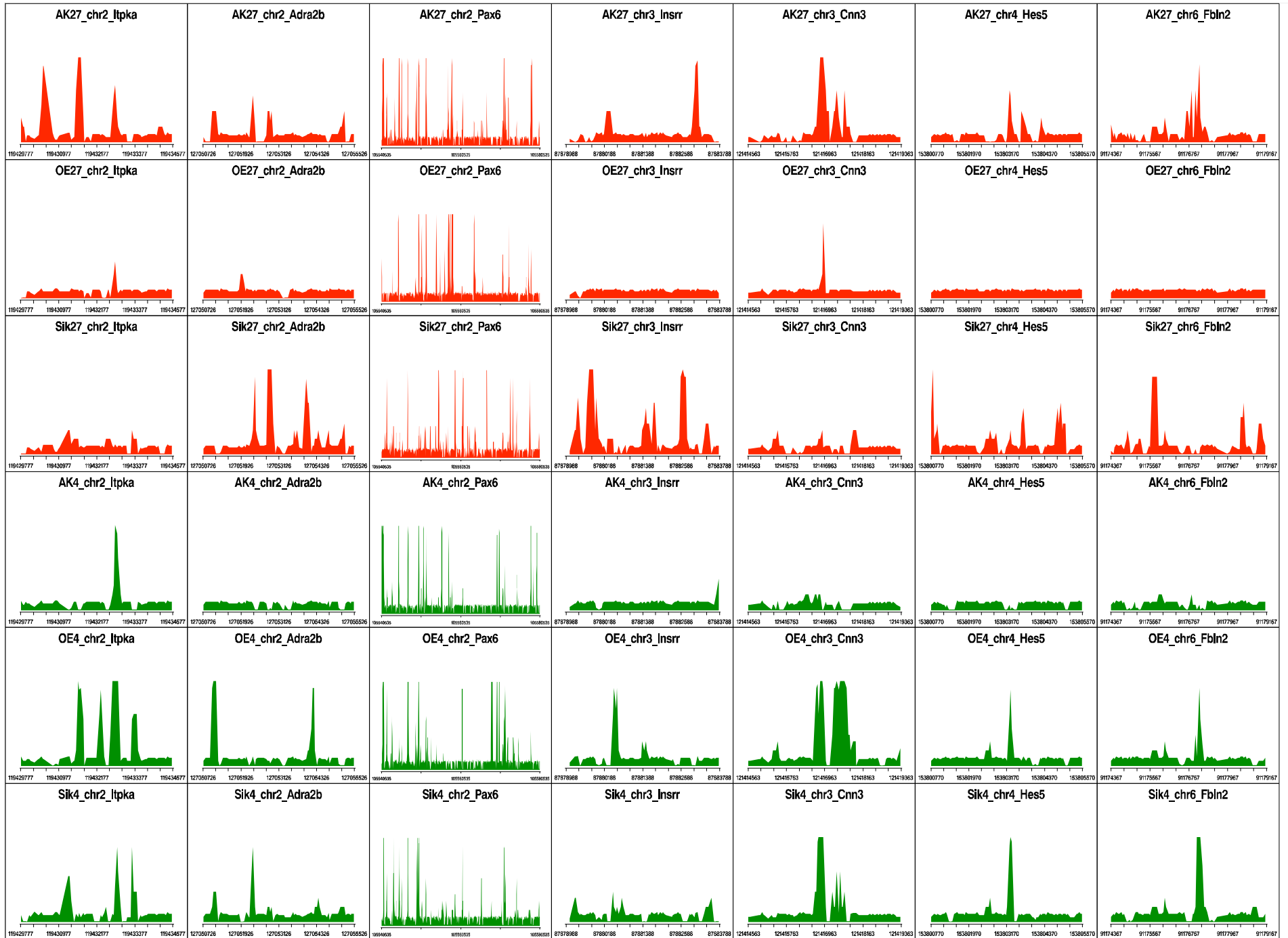

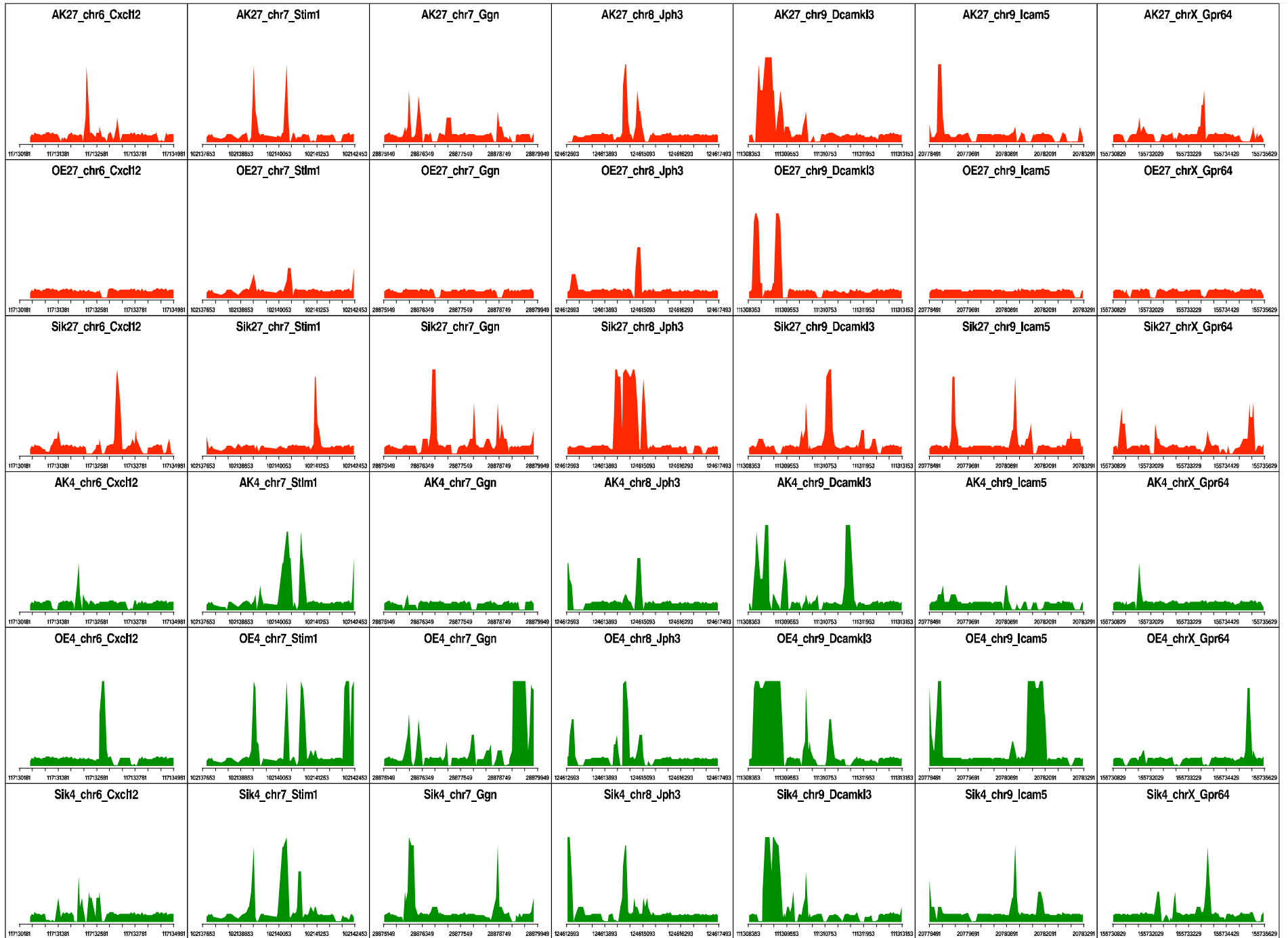

Supplement: Figure S5 — H3-K27me3 (red) and H3-K4me3 (green) patterns at promoters of genes activated following c-Myc overexpression. DNA immunprecipitated with anti-H3-K4me3 or anti-H3-K27me3 was applied to the custom-designed array (see text). Enrichment ratios (log2 scale) for ChIP-enriched versus total input genomic DNA for 427 genes were processed by ACME and assigned p-values (-log10; y axis) identifying significant sites were plotted (see Methods). Red peaks present H3-K27me3 and green peaks present H3-K4me3. (1.49 MB PDF) [file pone.0007839.s005.pdf]

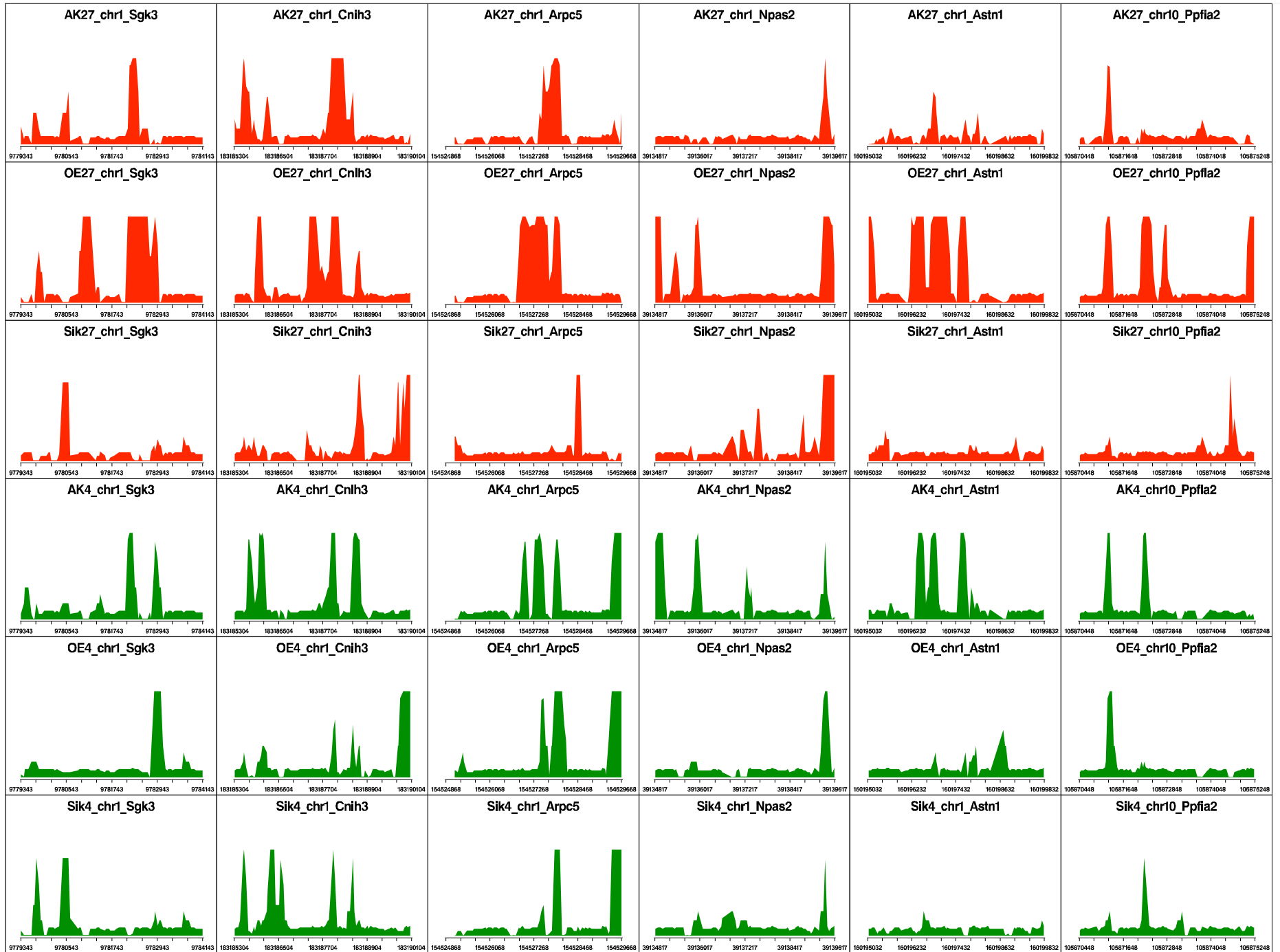

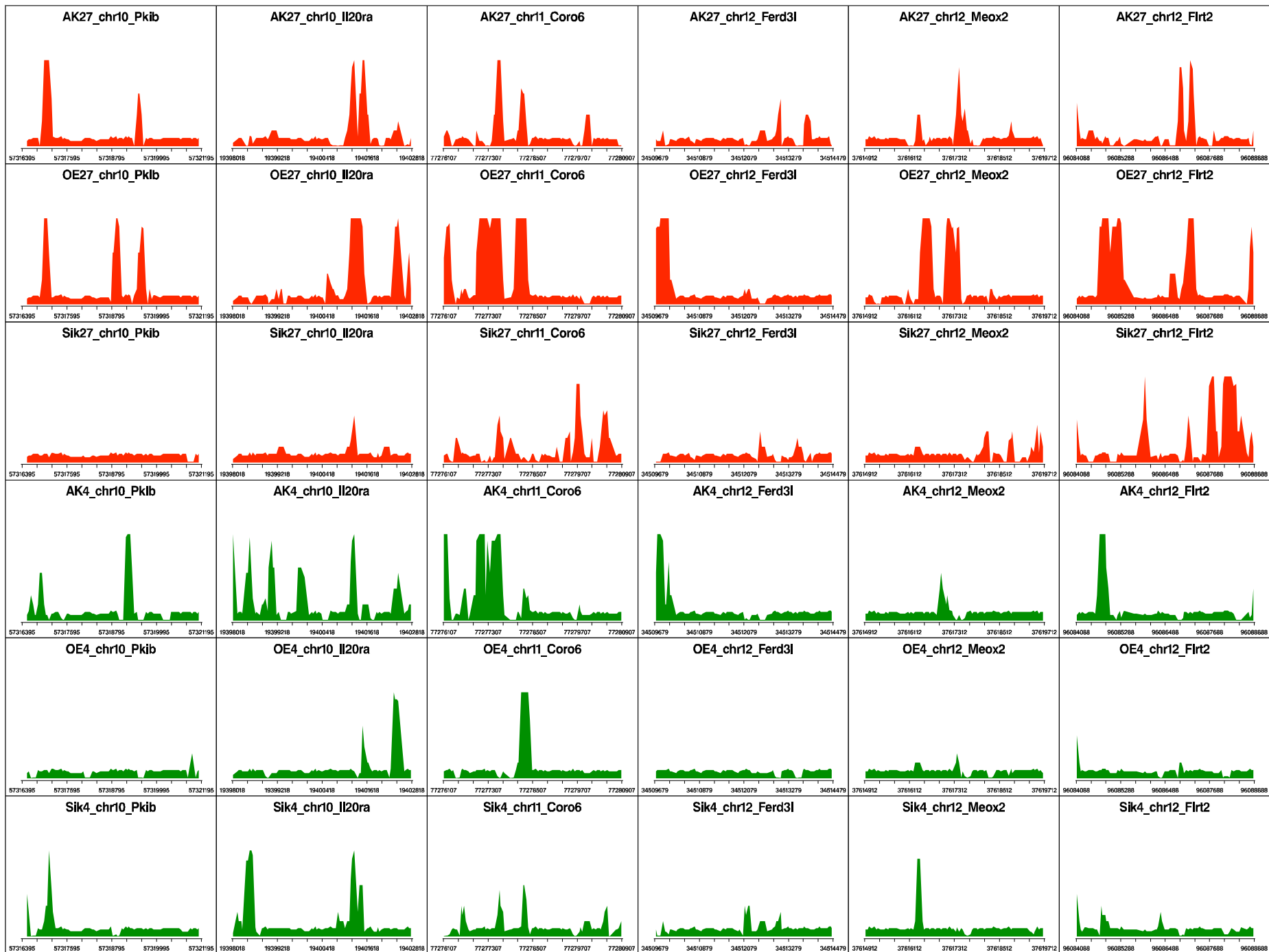

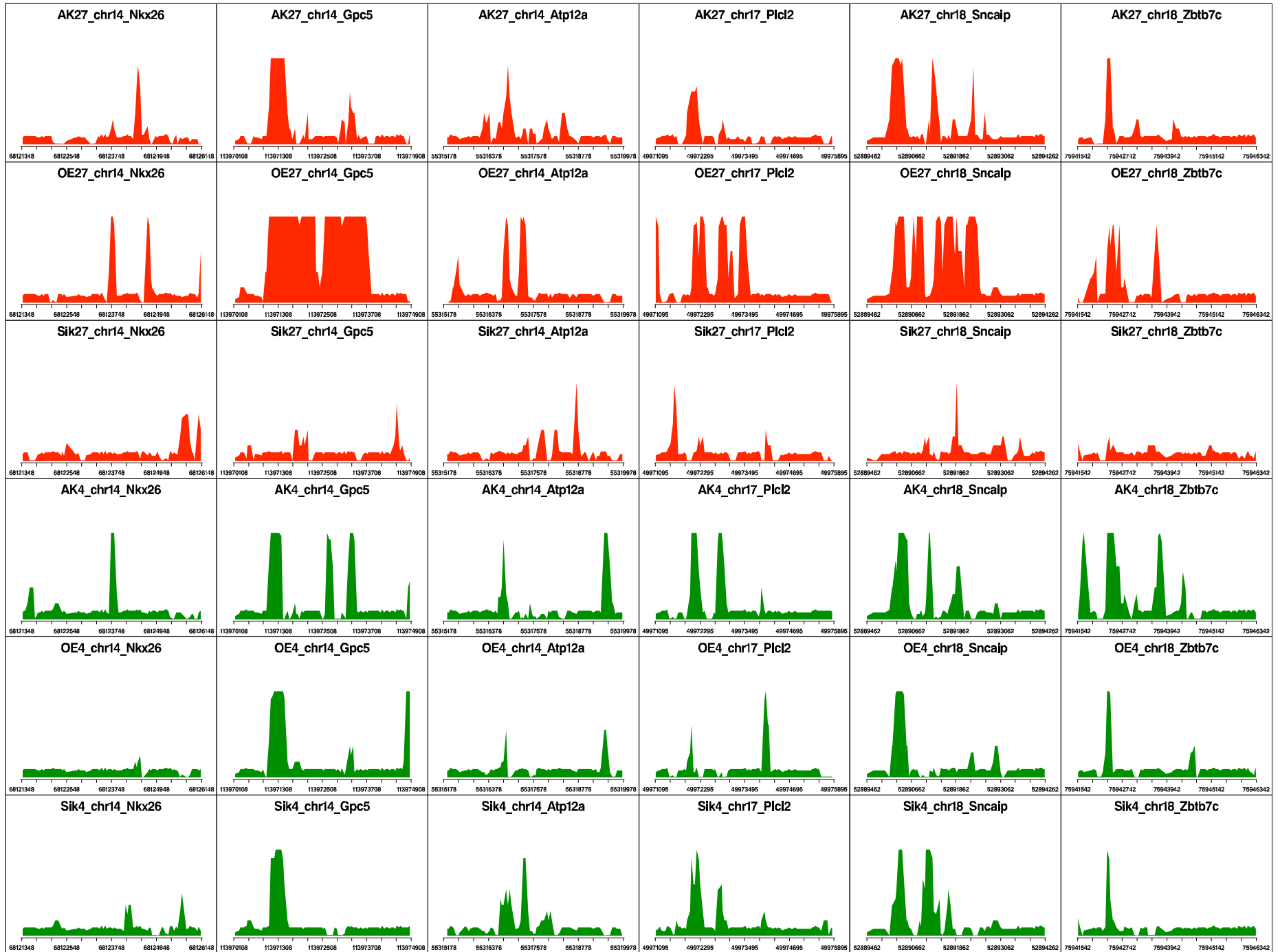

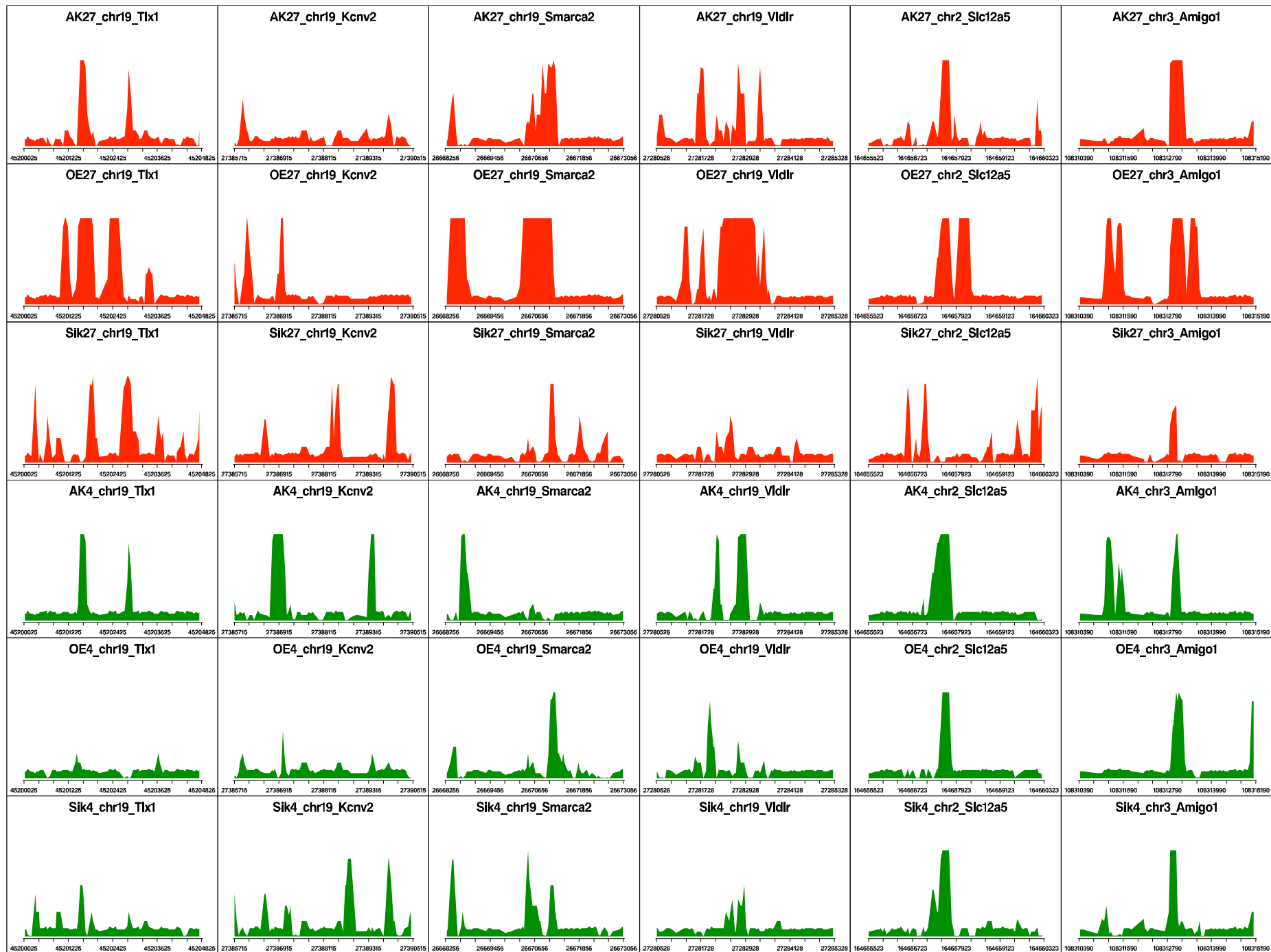

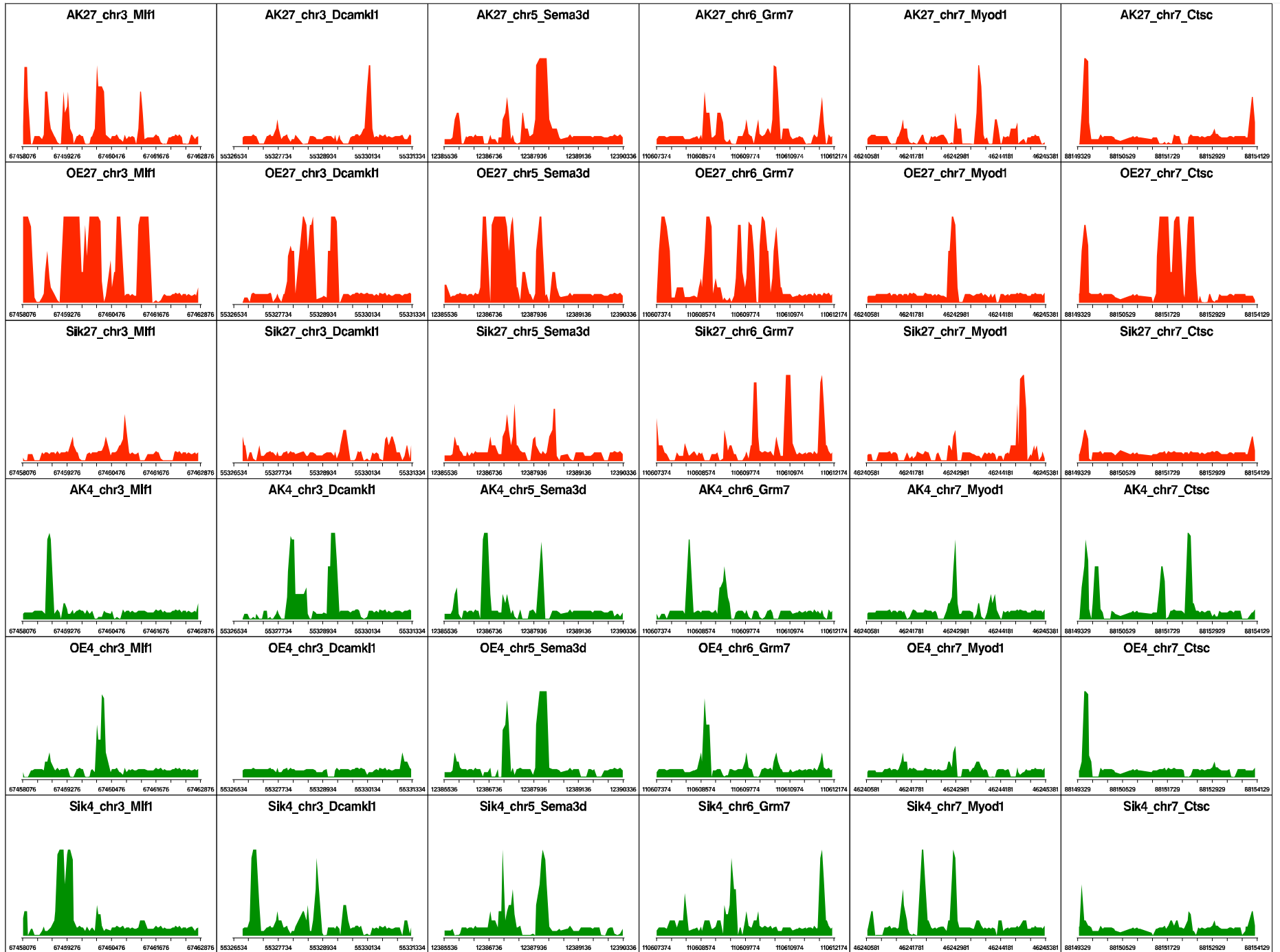

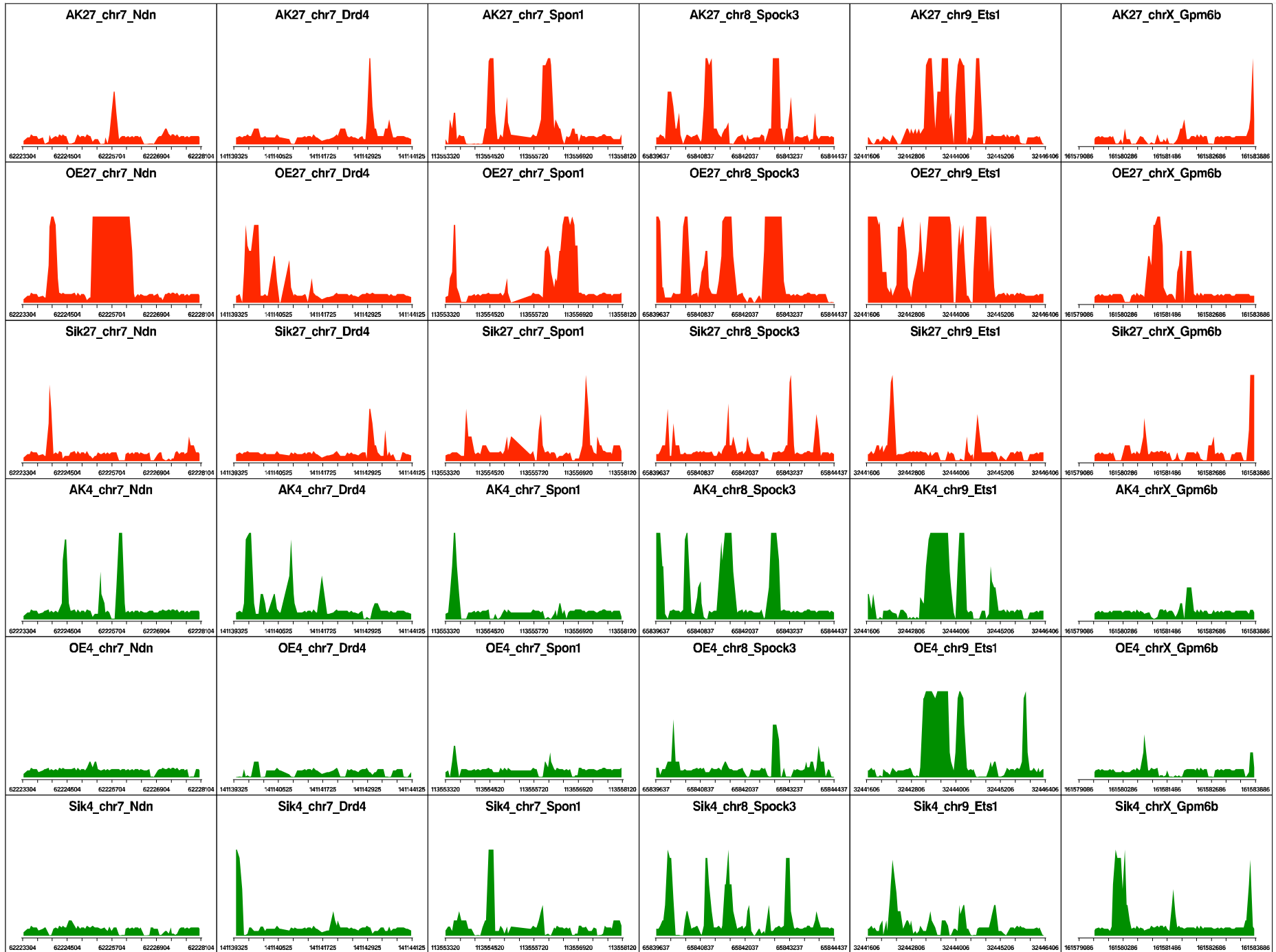

Supplement: Figure S6 — H3-K27me3 (red) and H3-K4me3 (green) patterns at promoters of genes repressed following c-Myc overexpression. DNA immunprecipitated with anti-H3-K4me3 or anti-H3-K27me3 was applied to the custom-designed array (see text). Enrichment ratios (log2 scale) for ChIP-enriched versus total input genomic DNA for 427 genes were processed by ACME and assigned p-values (-log10; y axis) identifying significant sites were plotted (see Methods). Red peaks present H3-K27me3 and green peaks present H3-K4me3. (1.14 MB PDF) [file pone.0007839.s006.pdf]

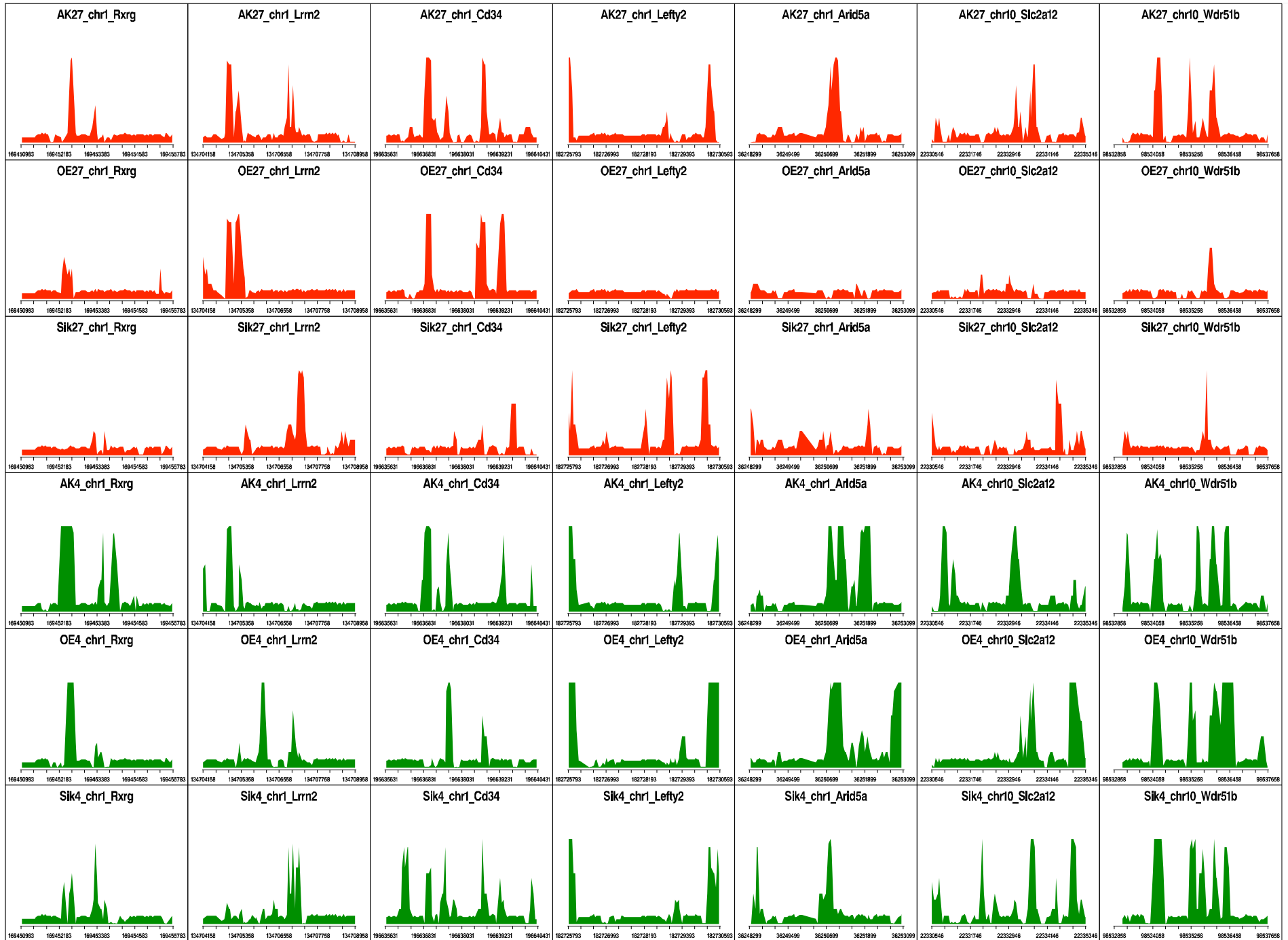

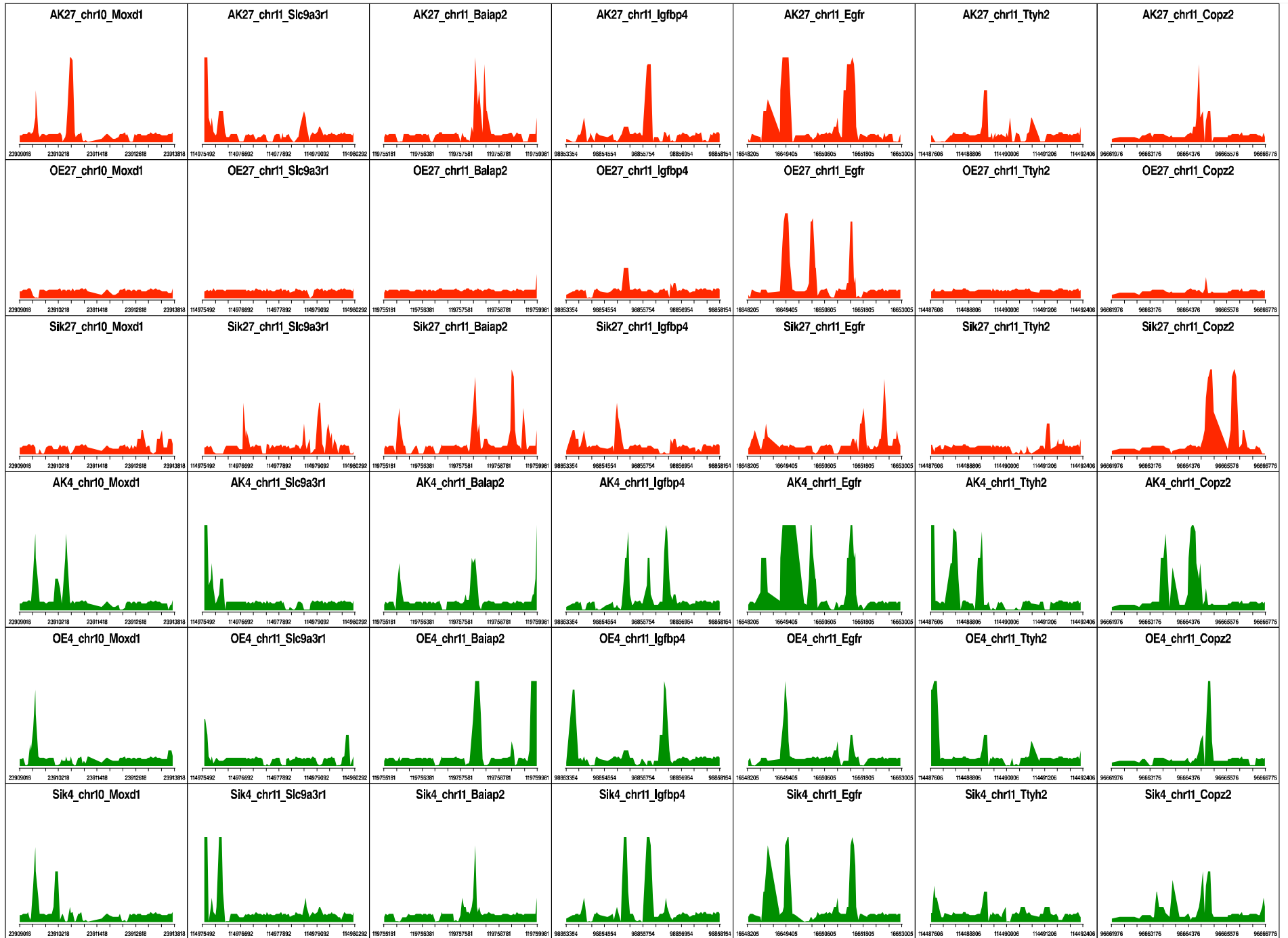

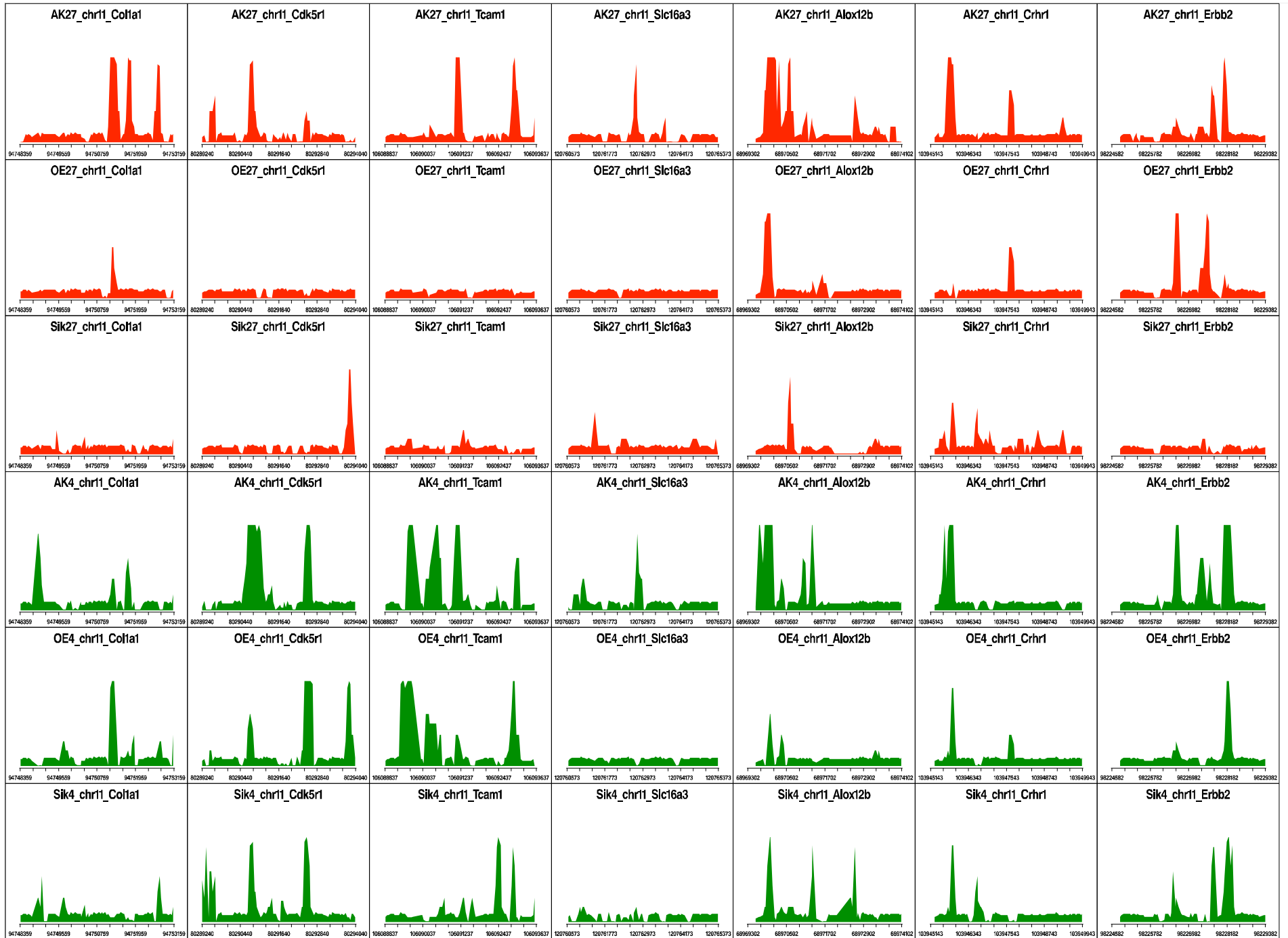

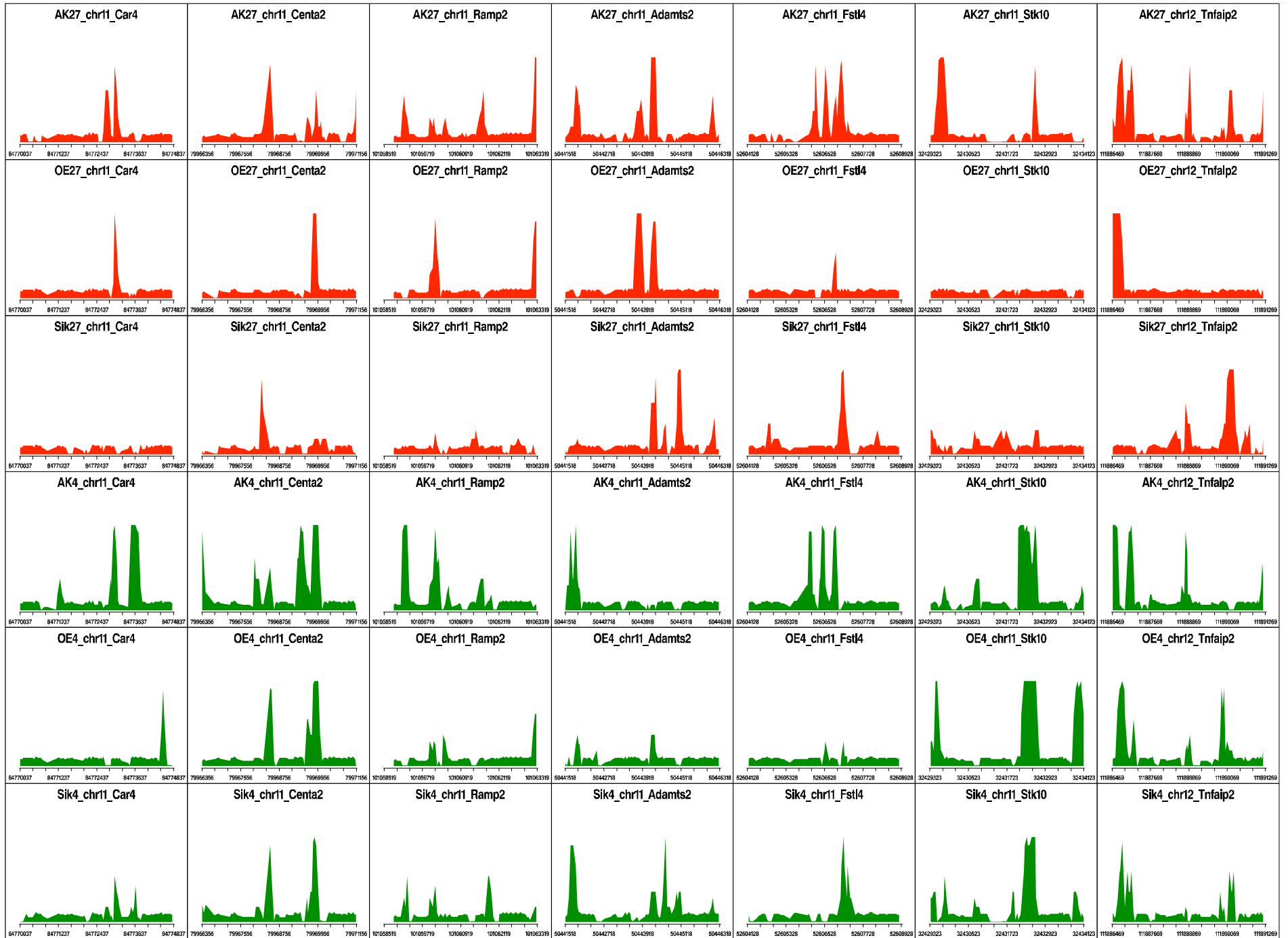

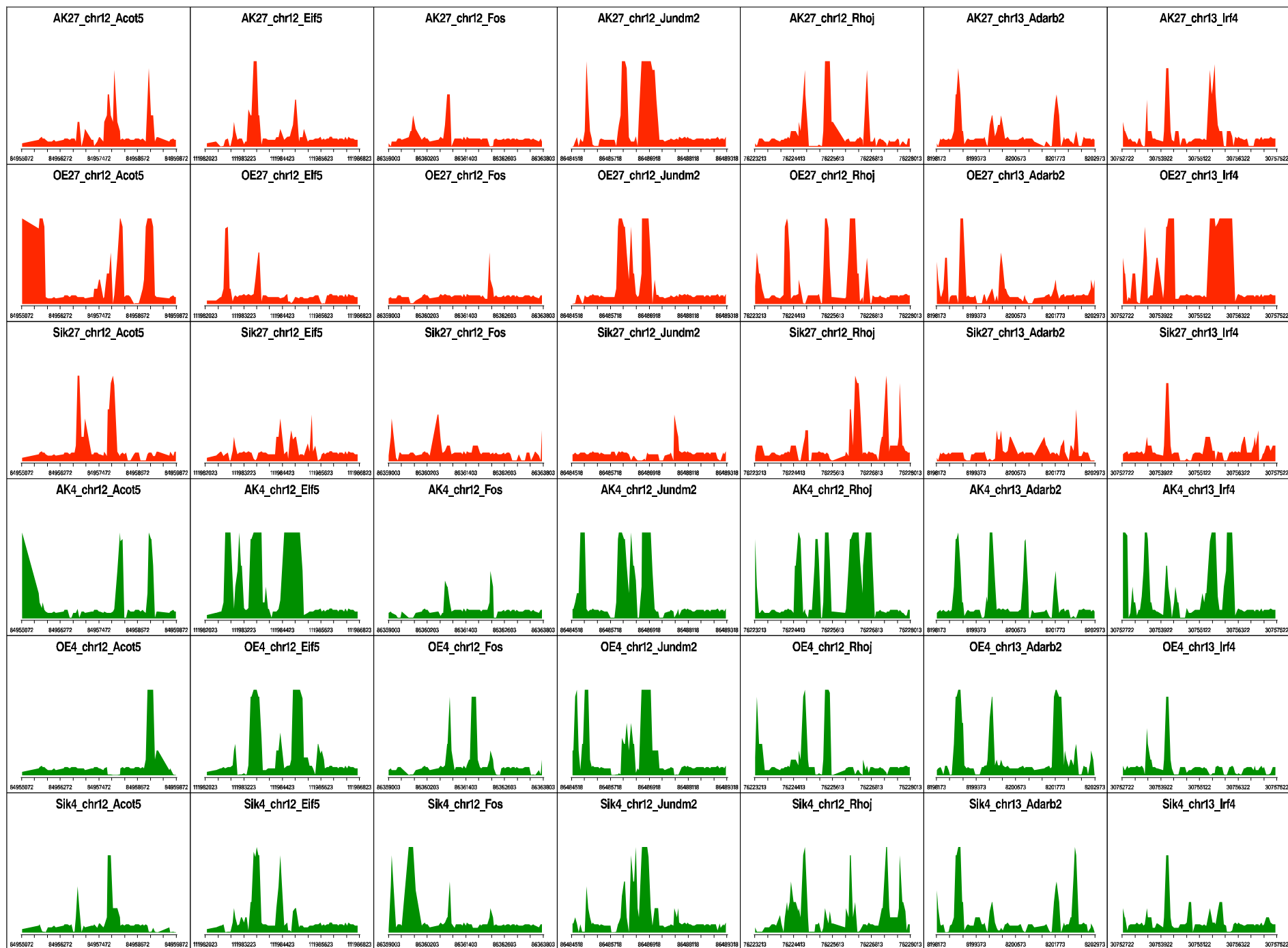

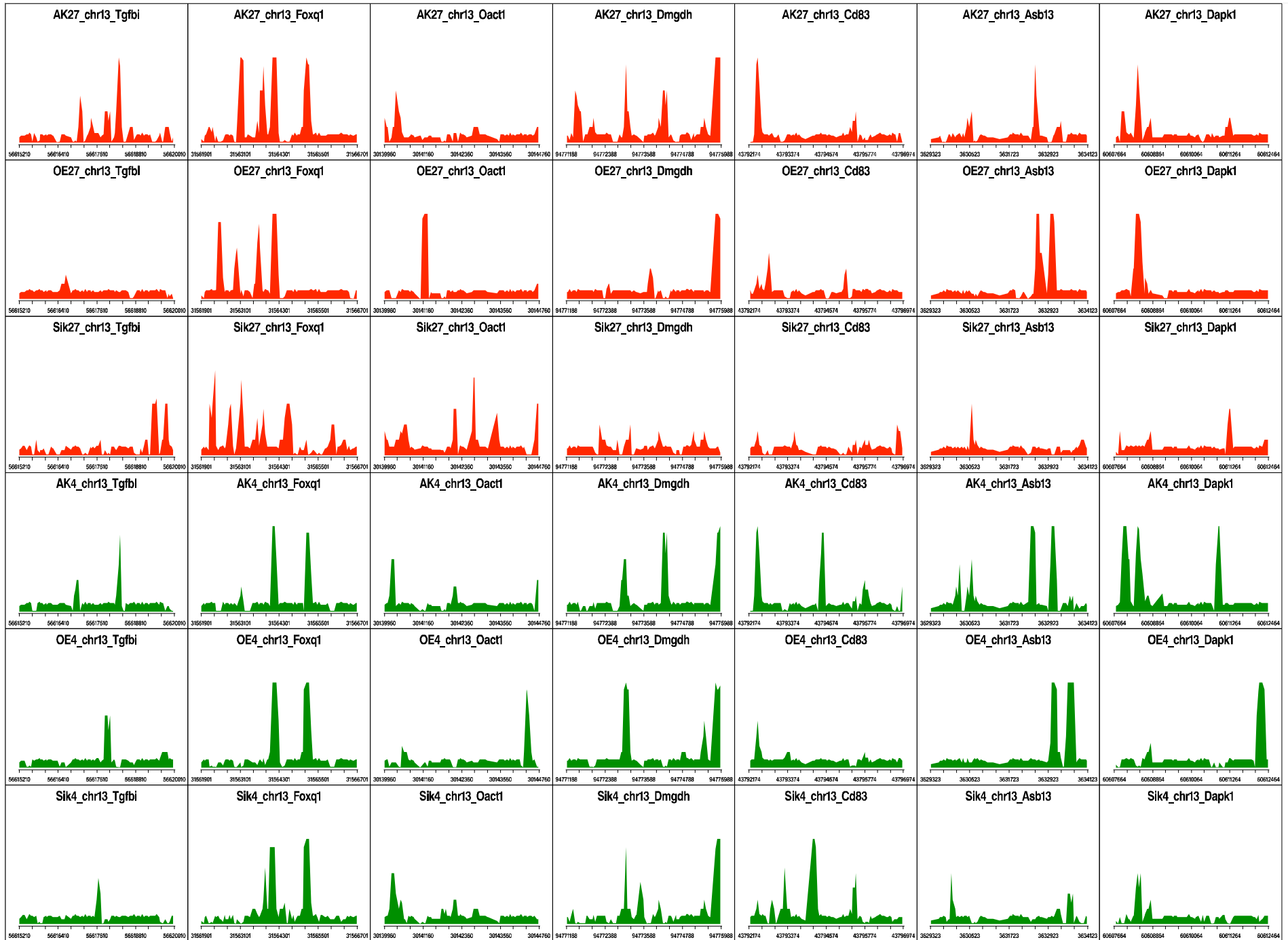

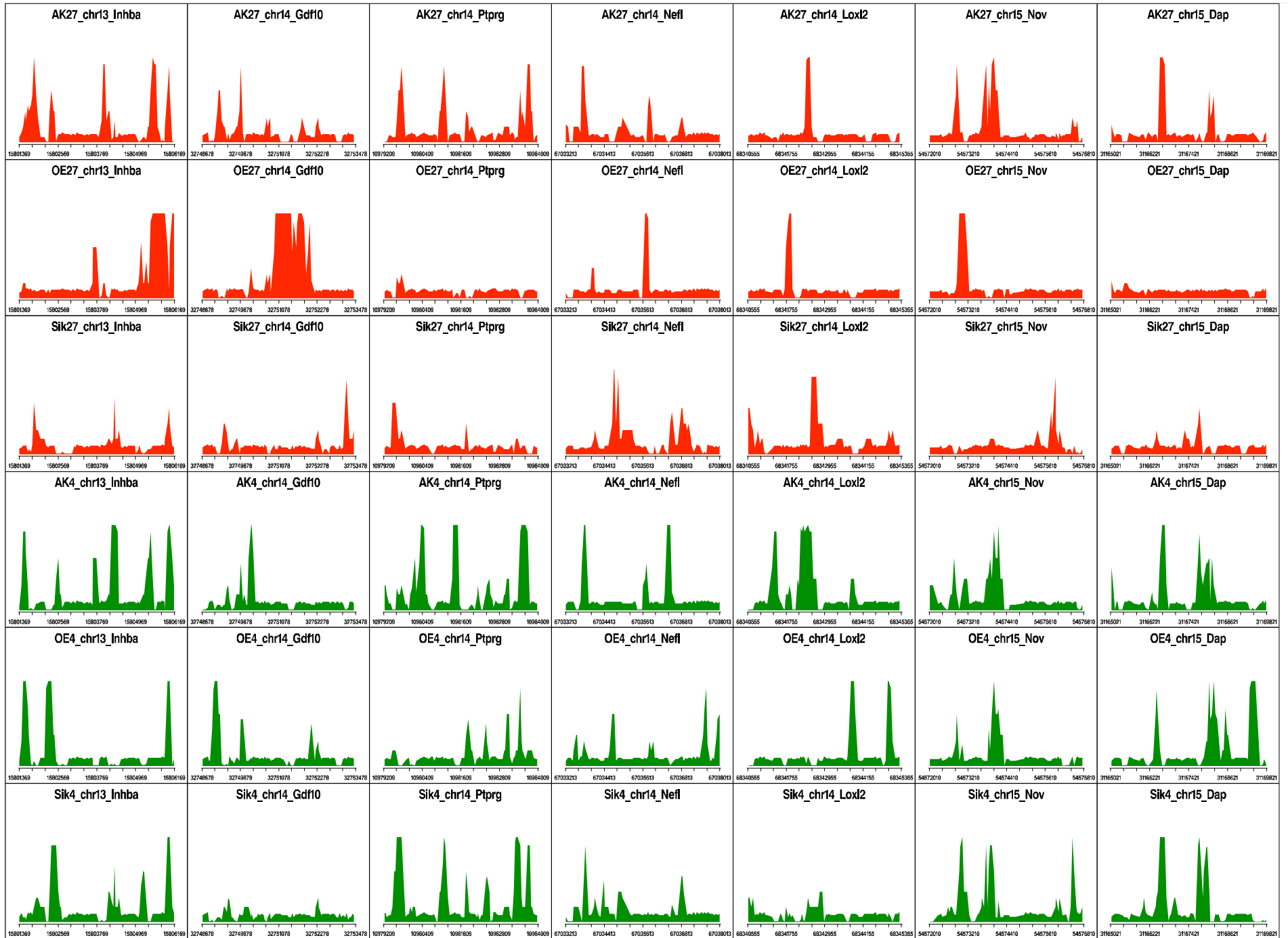

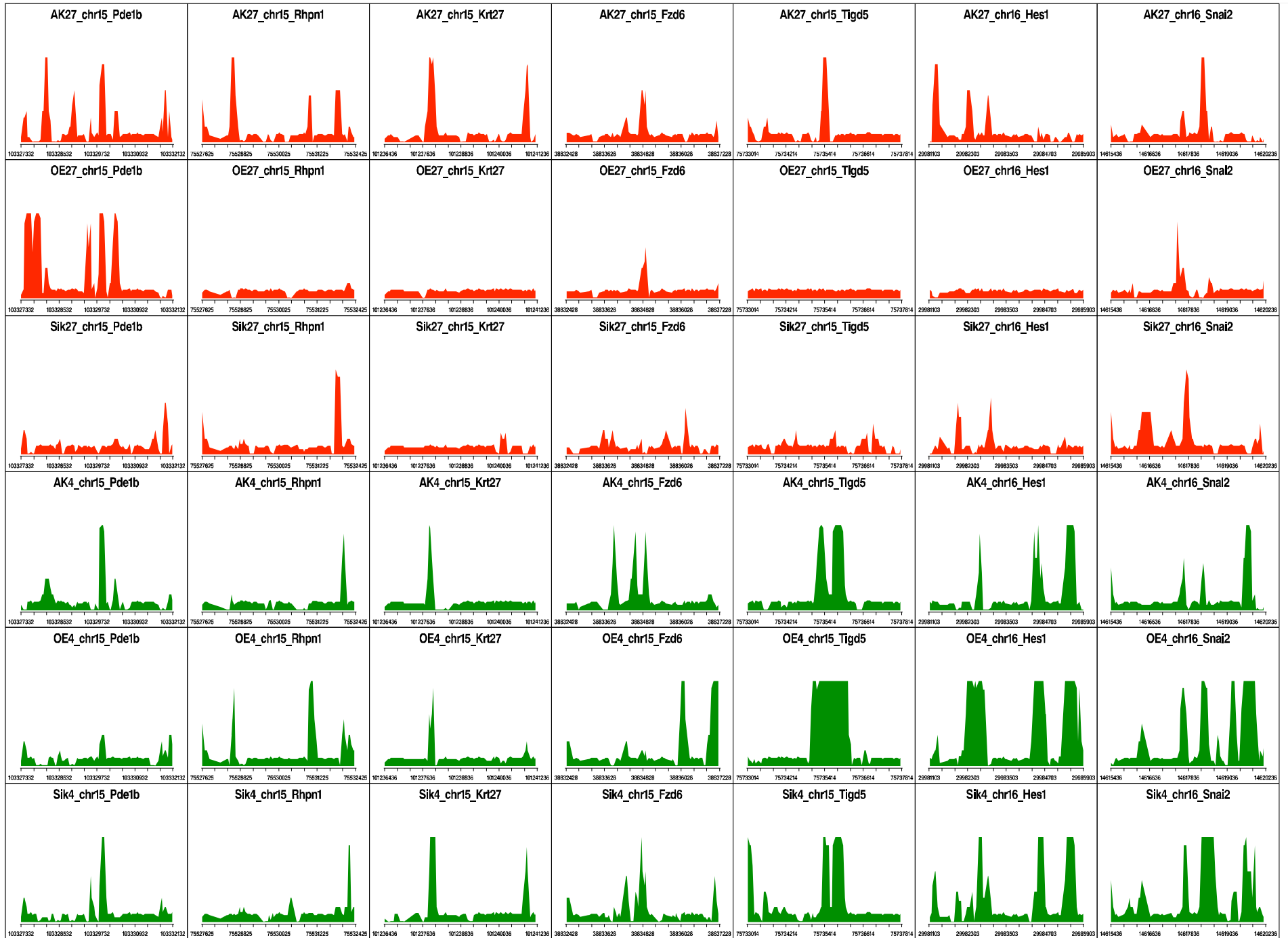

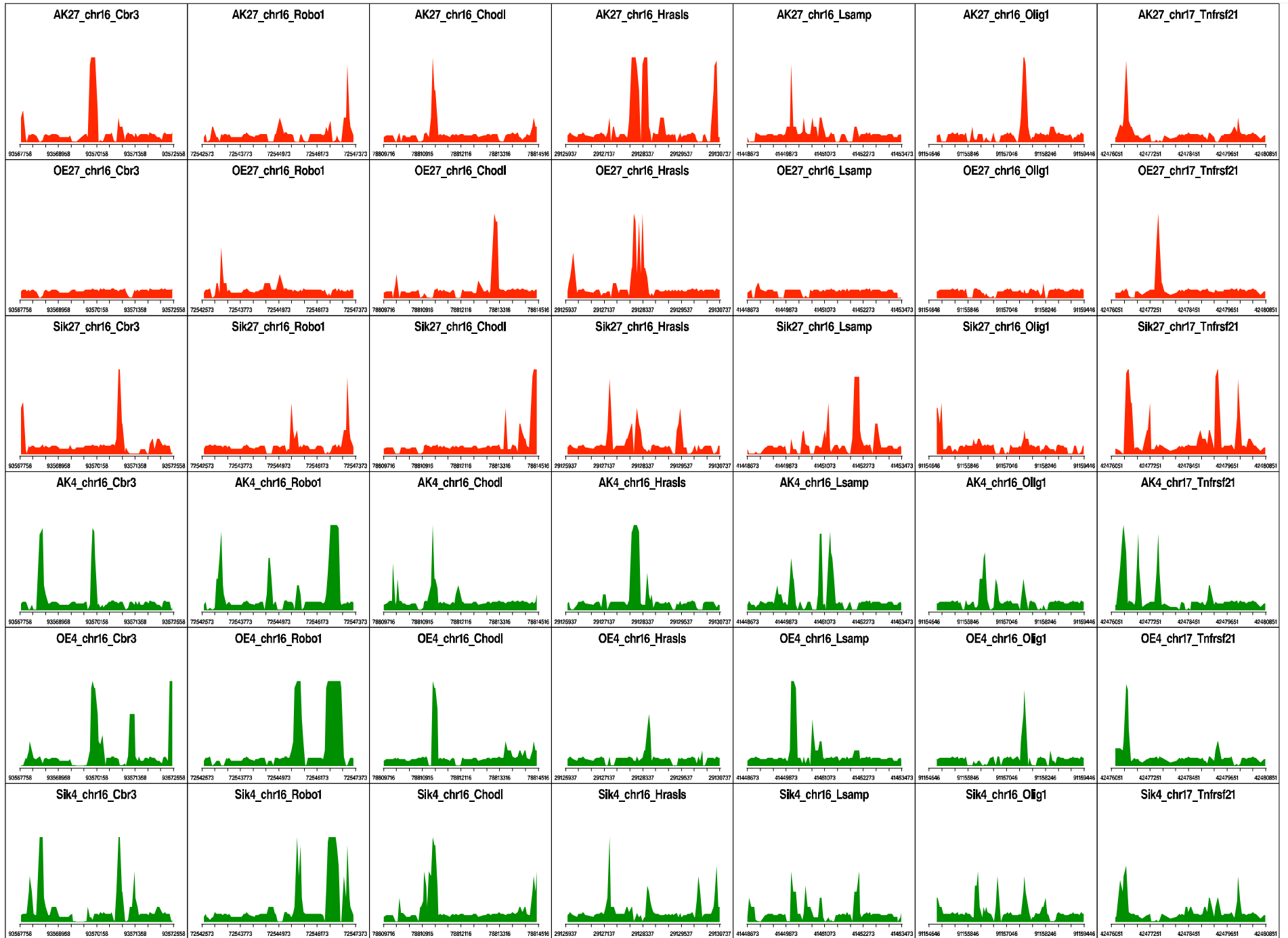

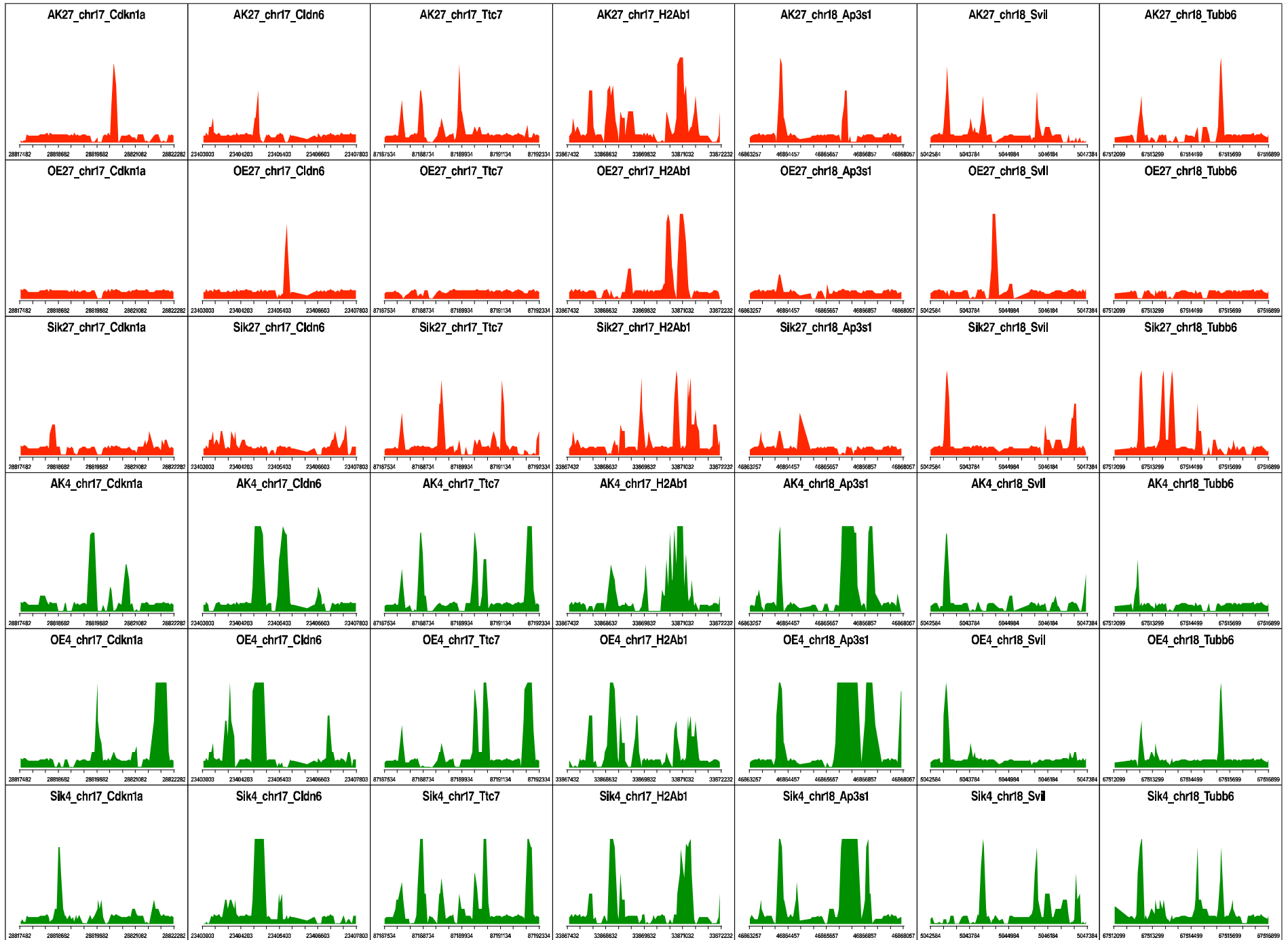

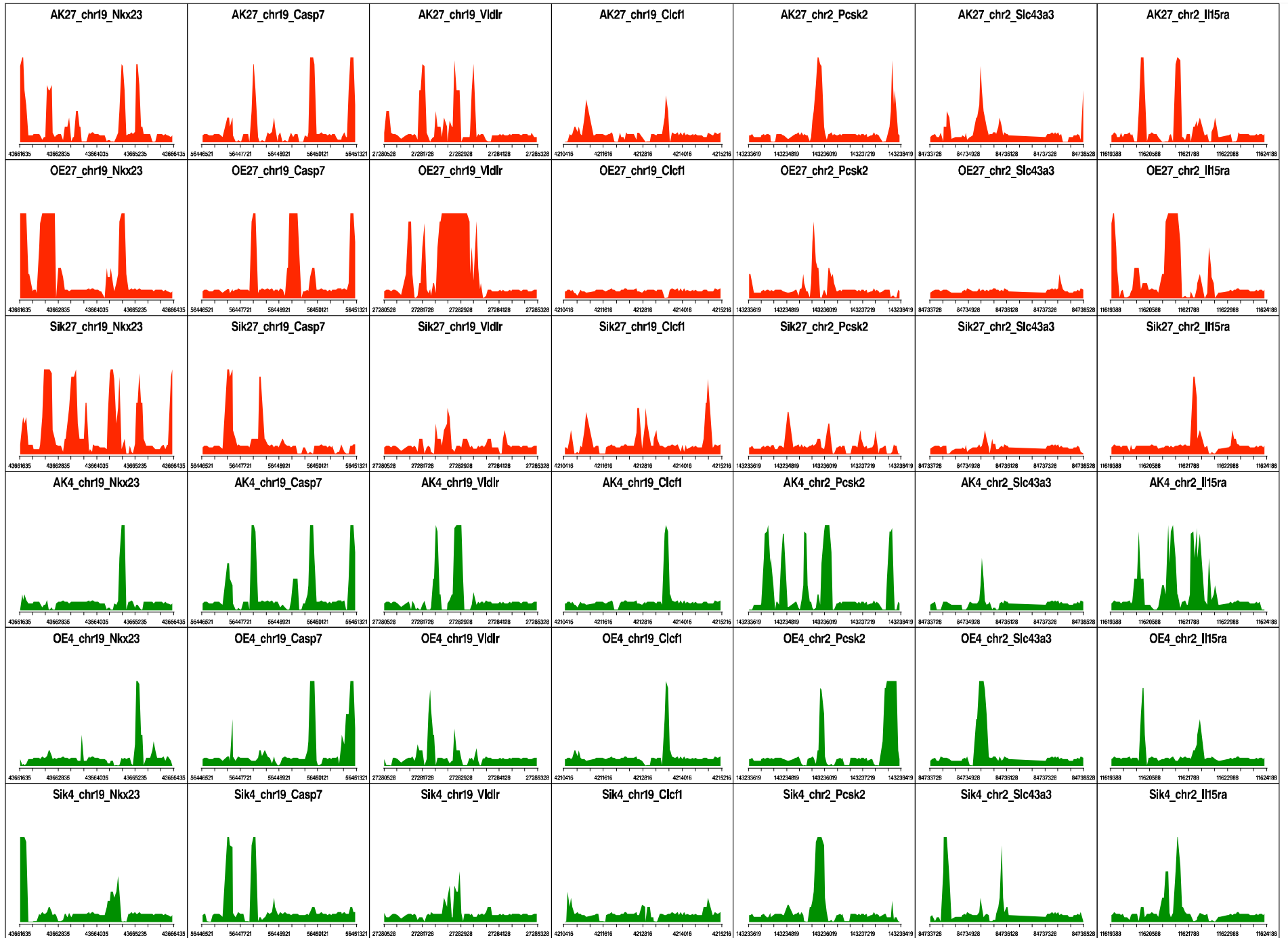

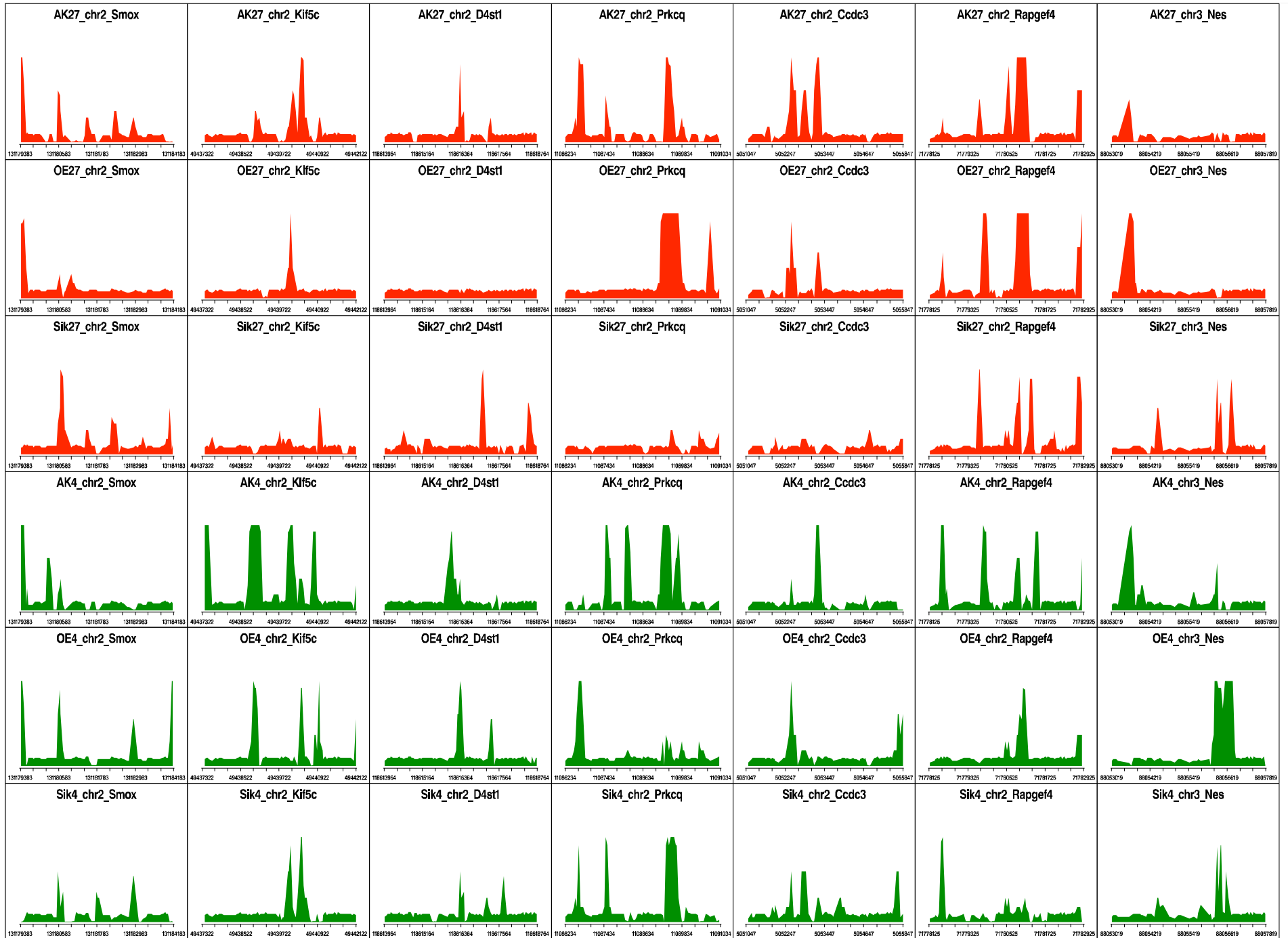

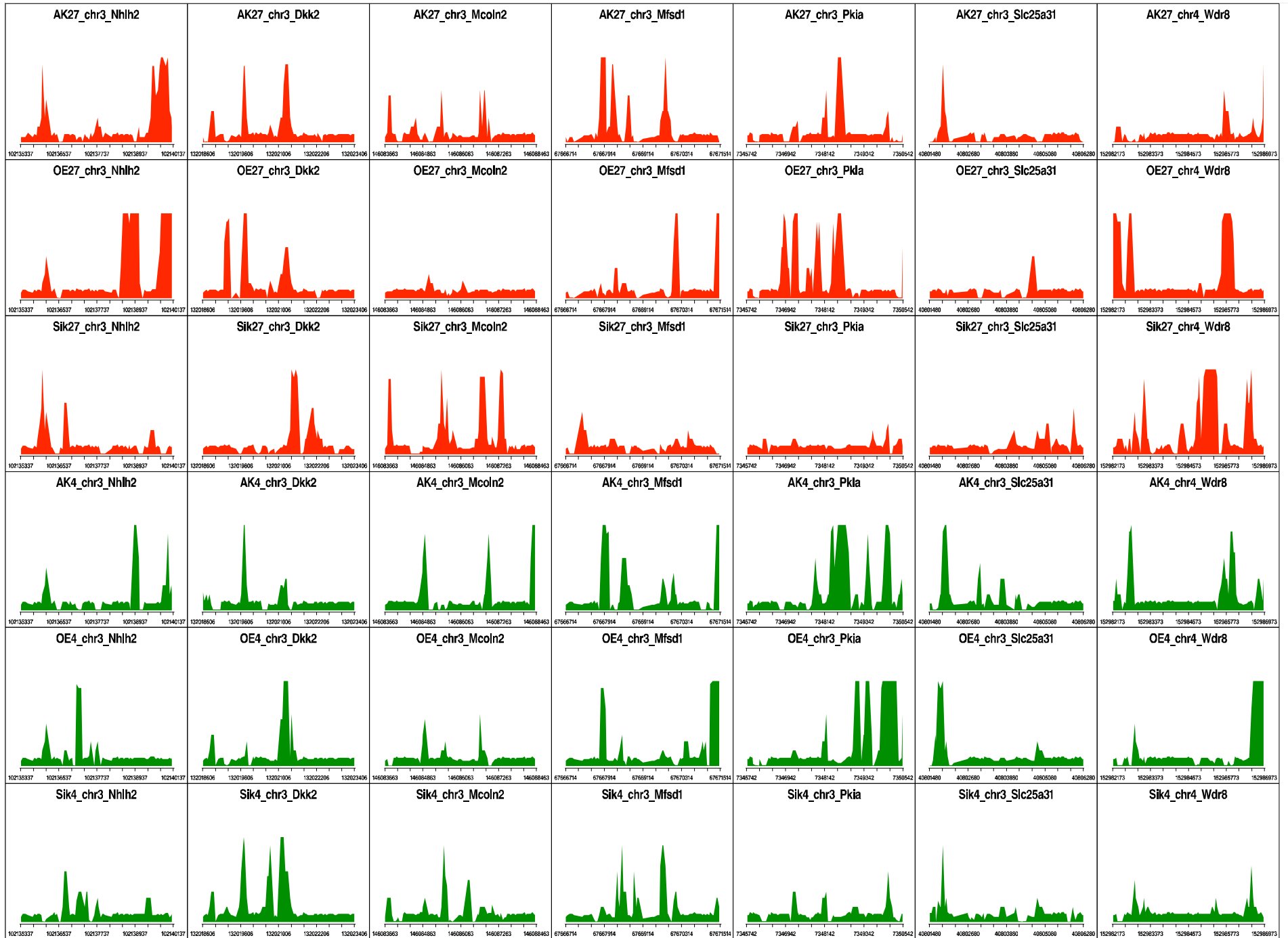

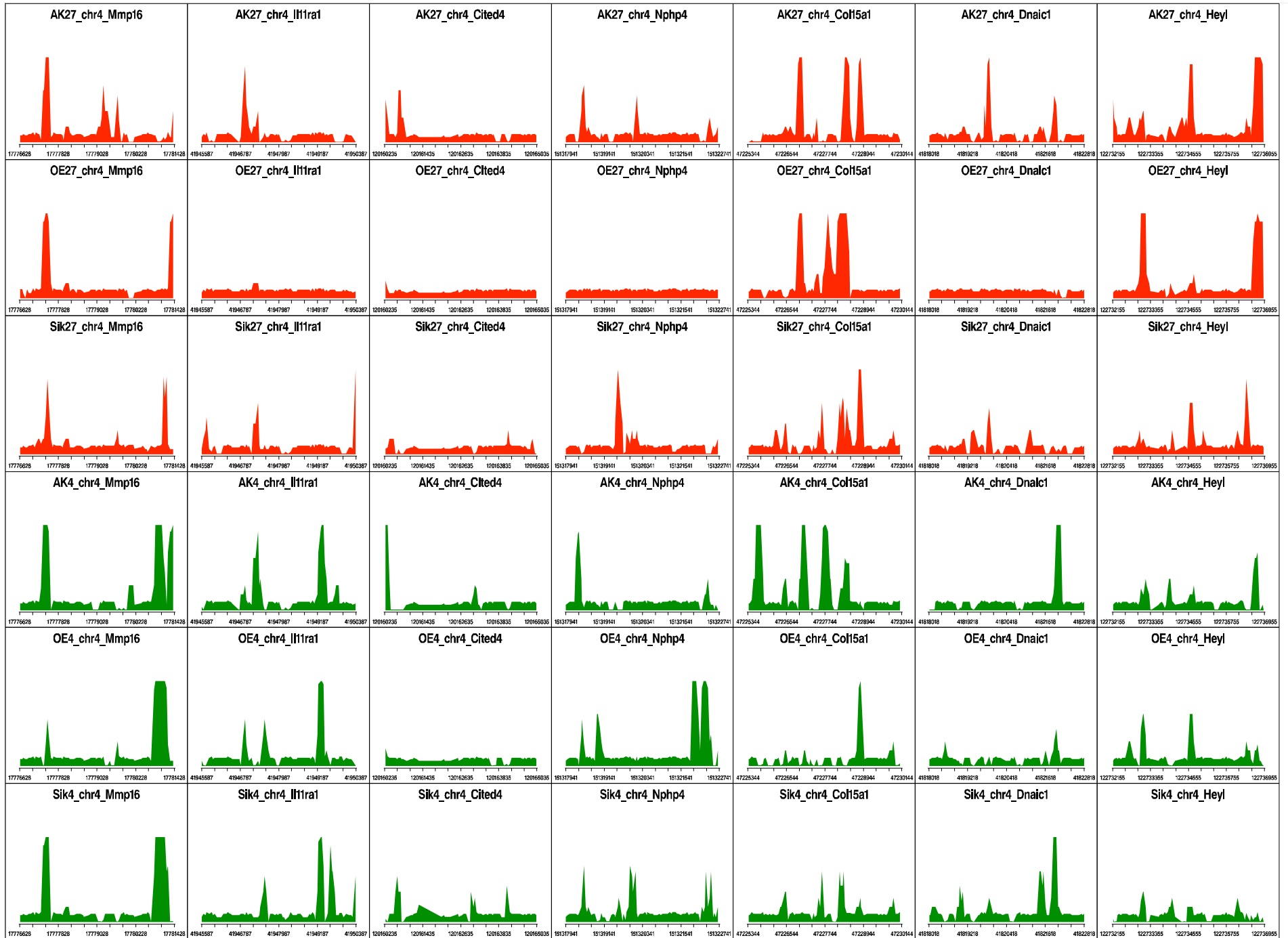

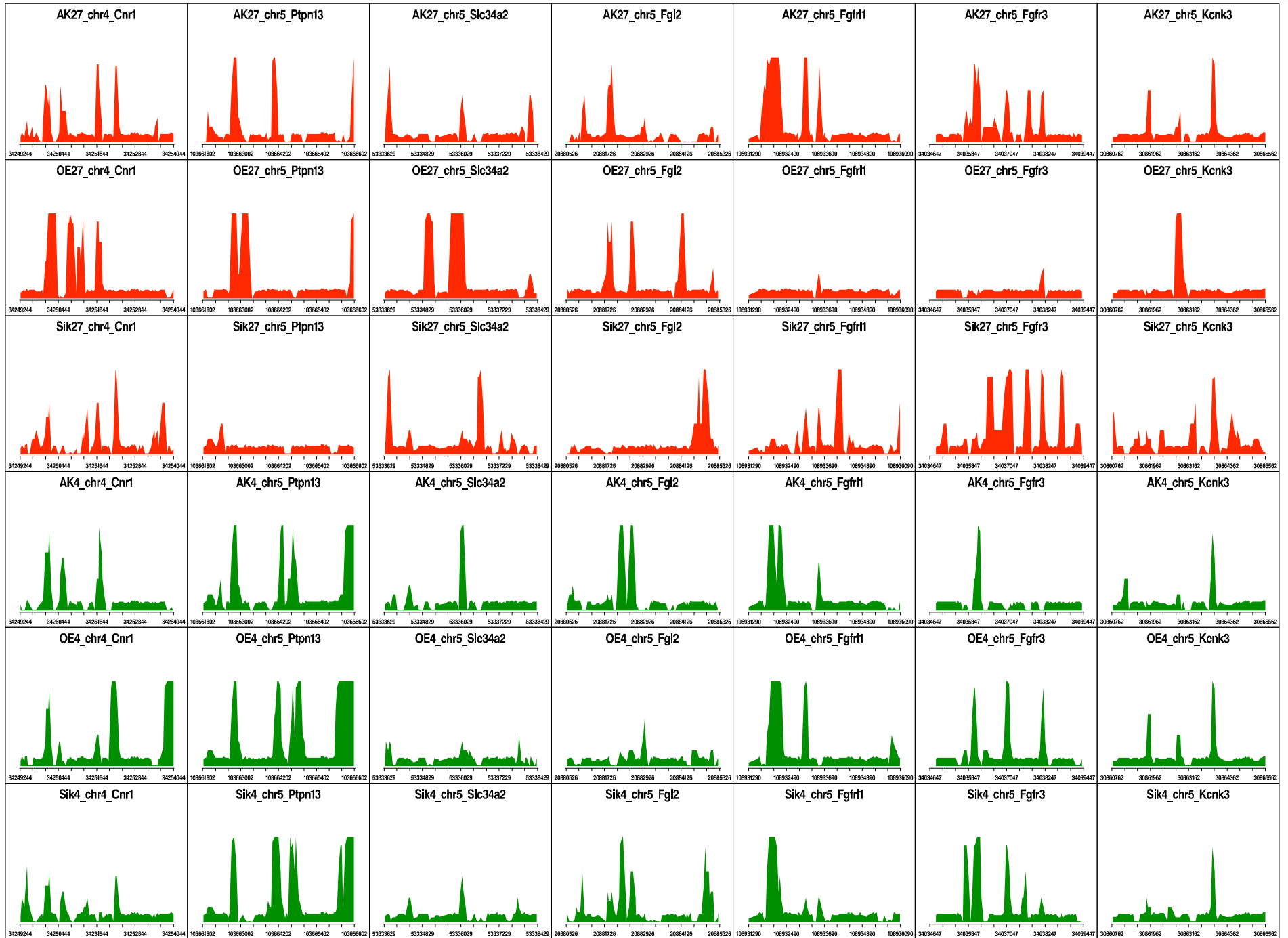

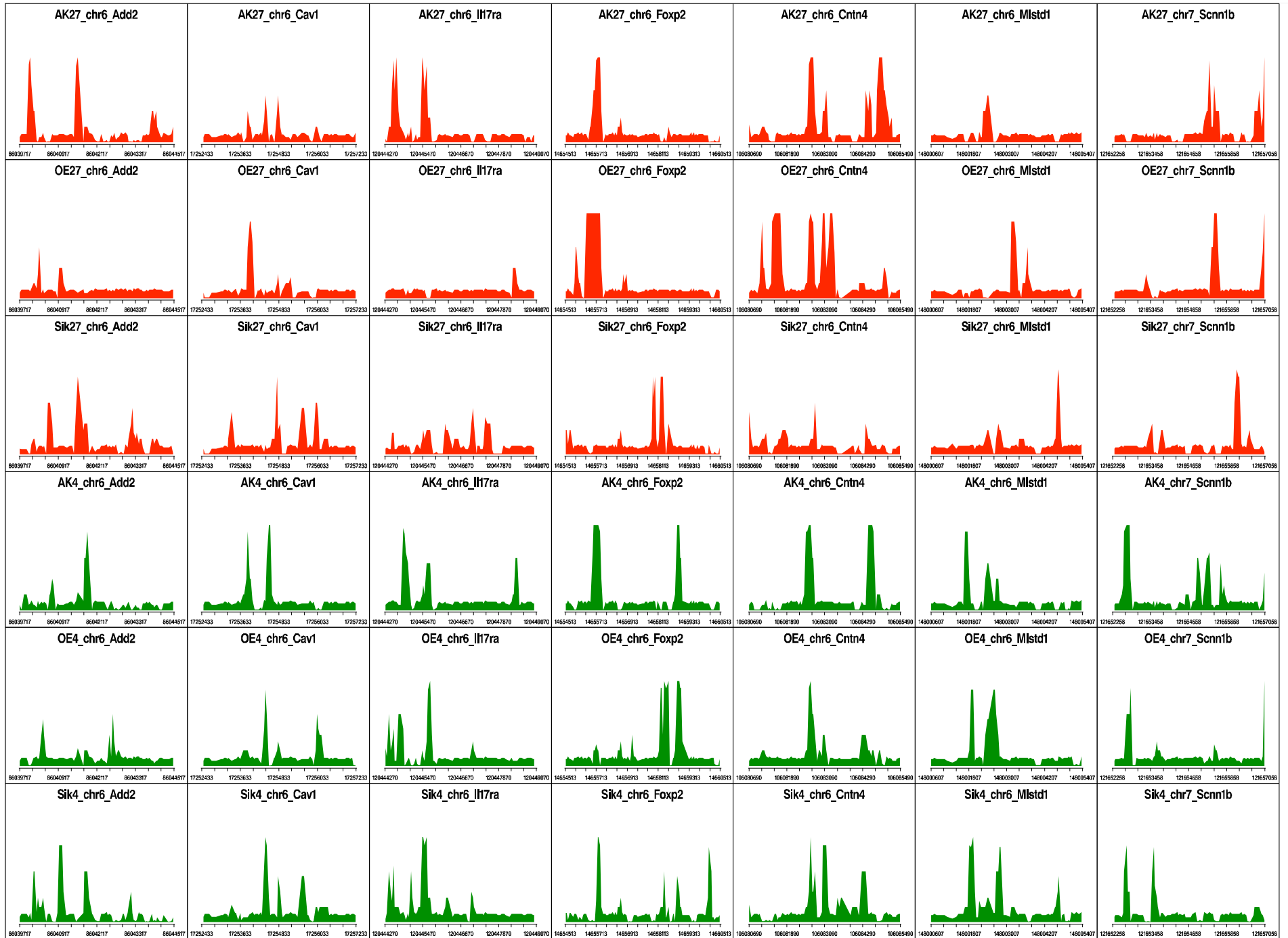

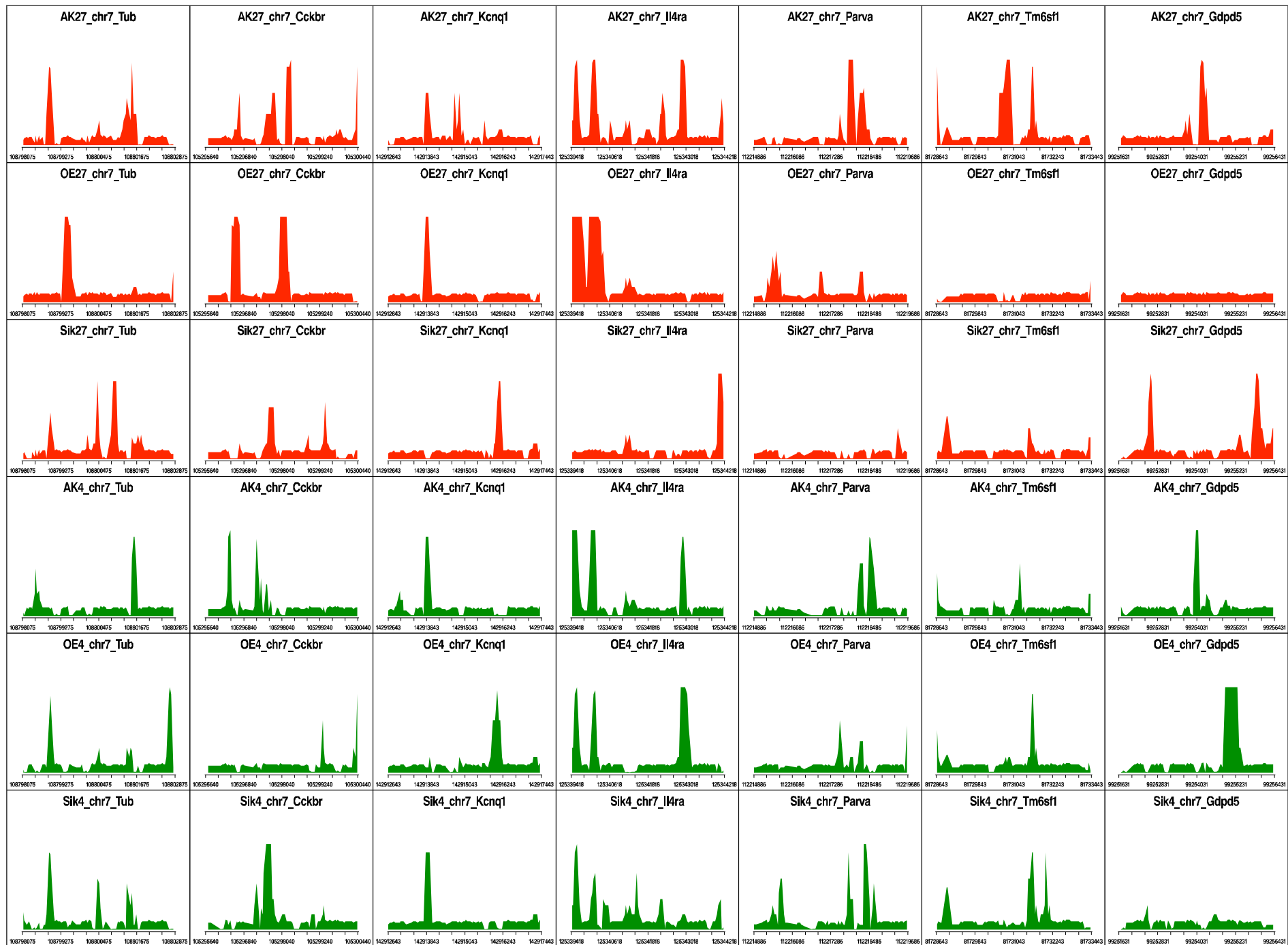

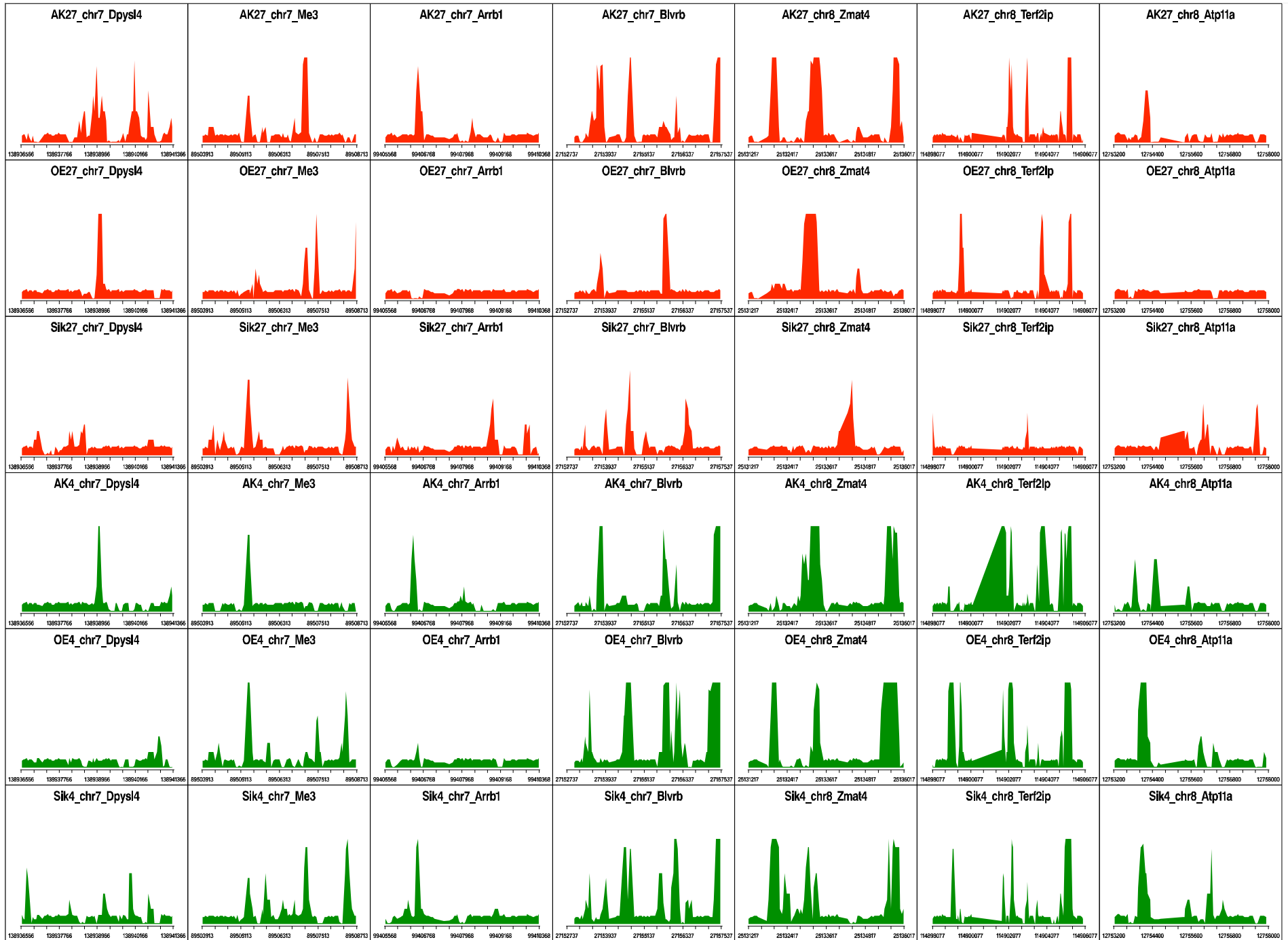

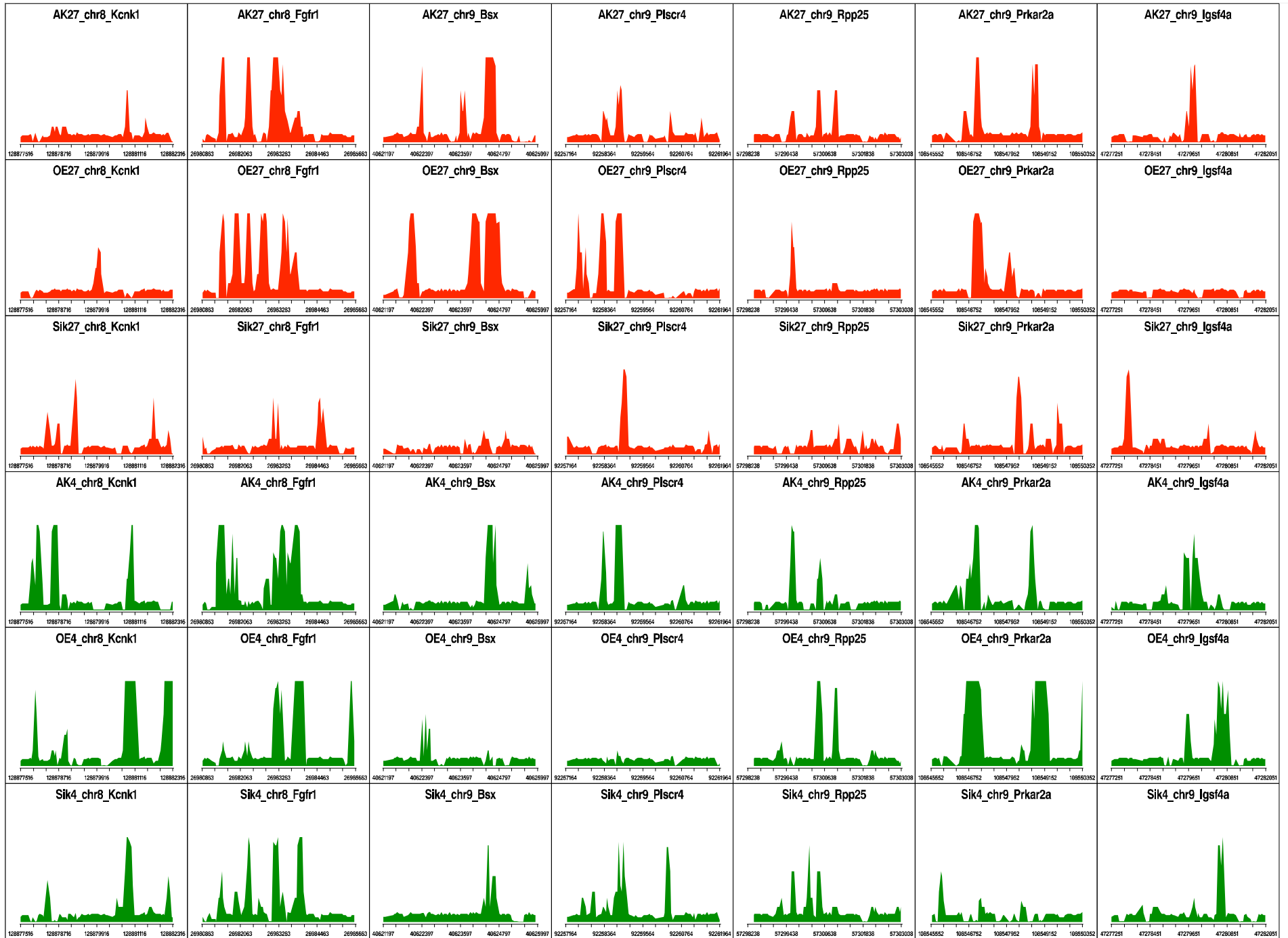

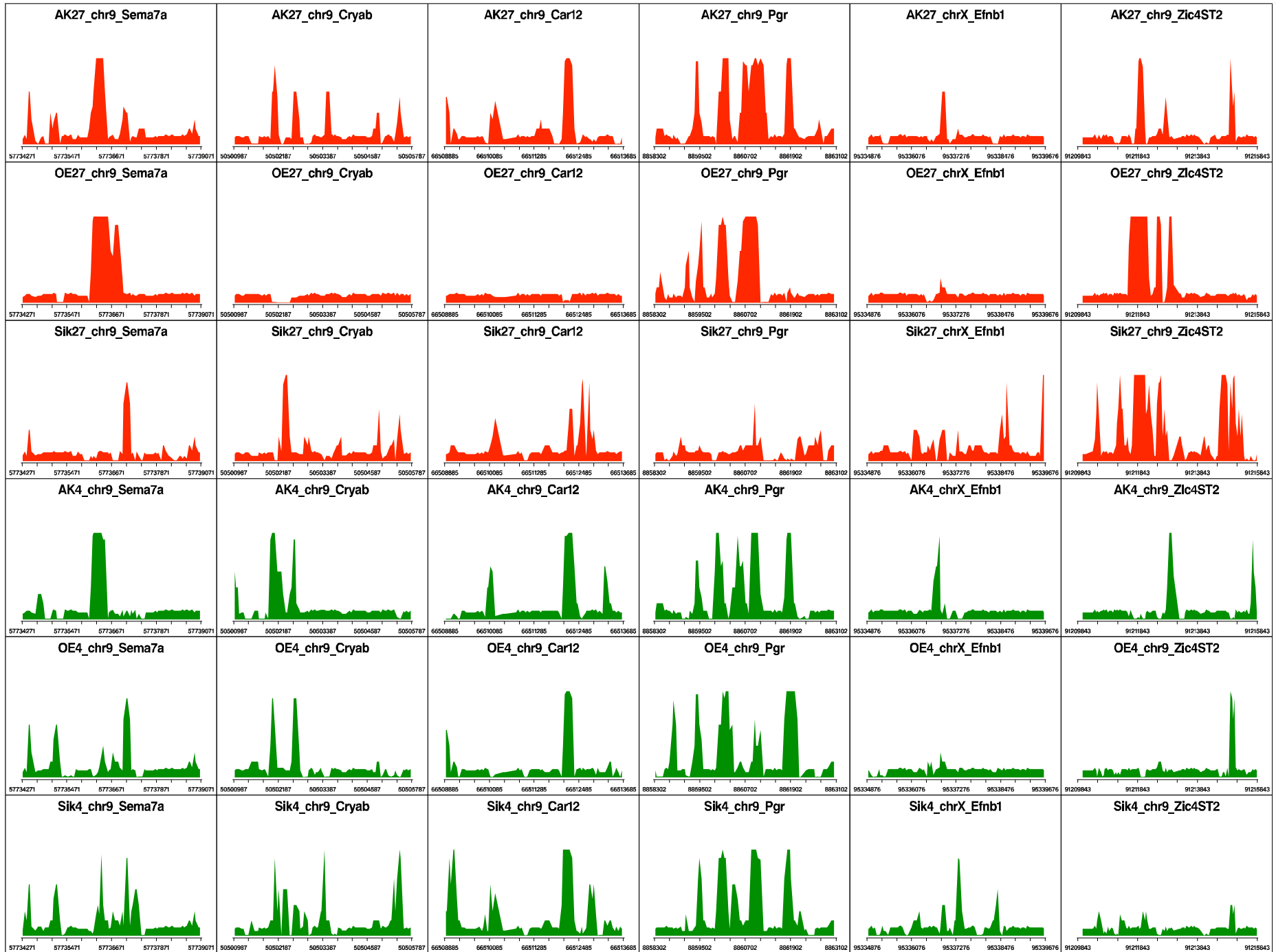

Supplement: Figure S7 — H3-K27me3 (red) and H3-K4me3 (green) patterns at promoters in which bivalency is partly lost upon c-Myc overexpression. DNA immunprecipitated with anti-H3-K4me3 or anti-H3-K27me3 was applied to the custom-designed array (see text). Enrichment ratios (log2 scale) for ChIP-enriched versus total input genomic DNA for 427 genes were processed by ACME and assigned p-values (-log10; y axis) identifying significant sites were plotted (see Methods). Red peaks present H3-K27me3 and green peaks present H3-K4me3. (4.21 MB PDF) [file pone.0007839.s007.pdf]

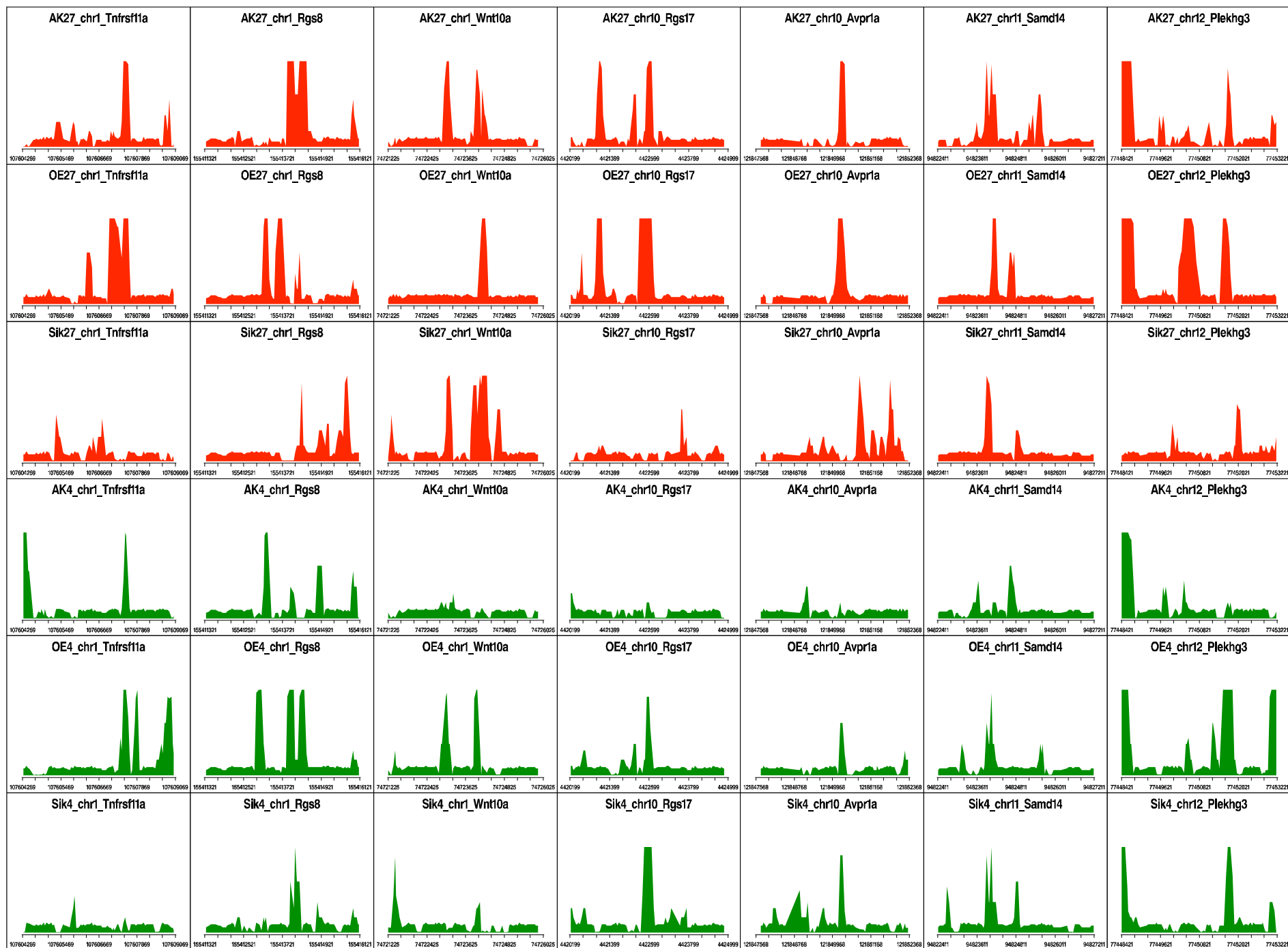

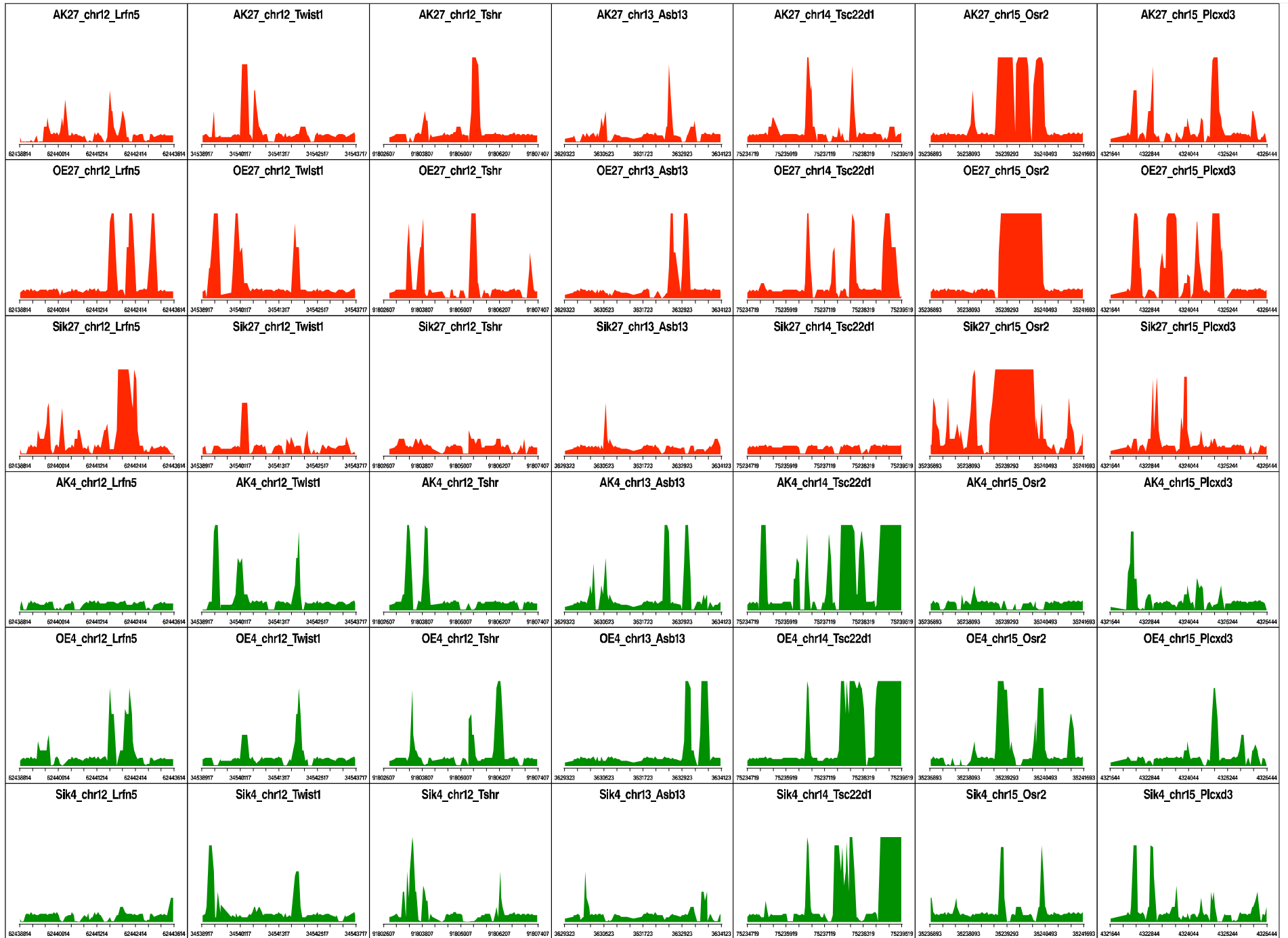

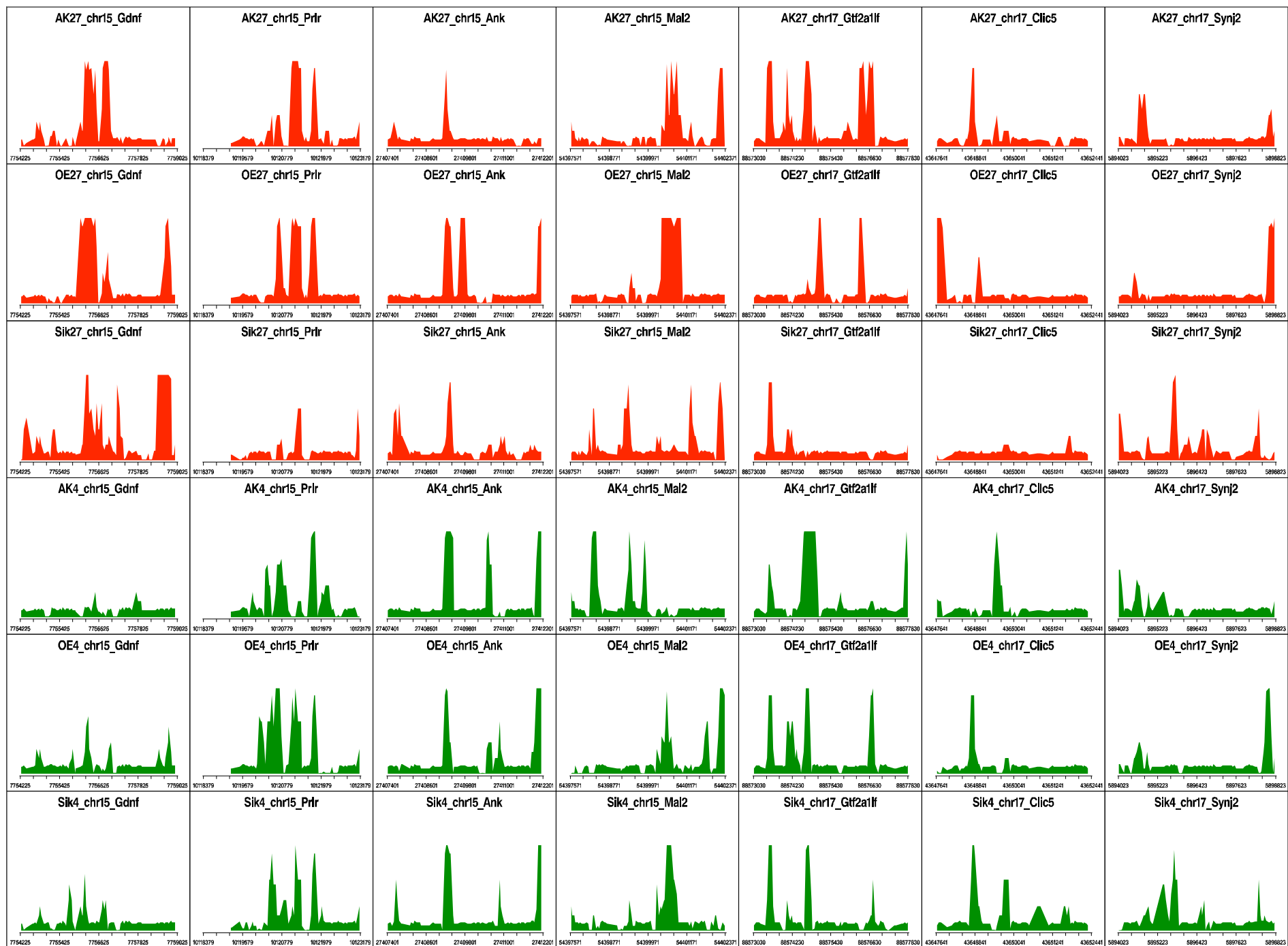

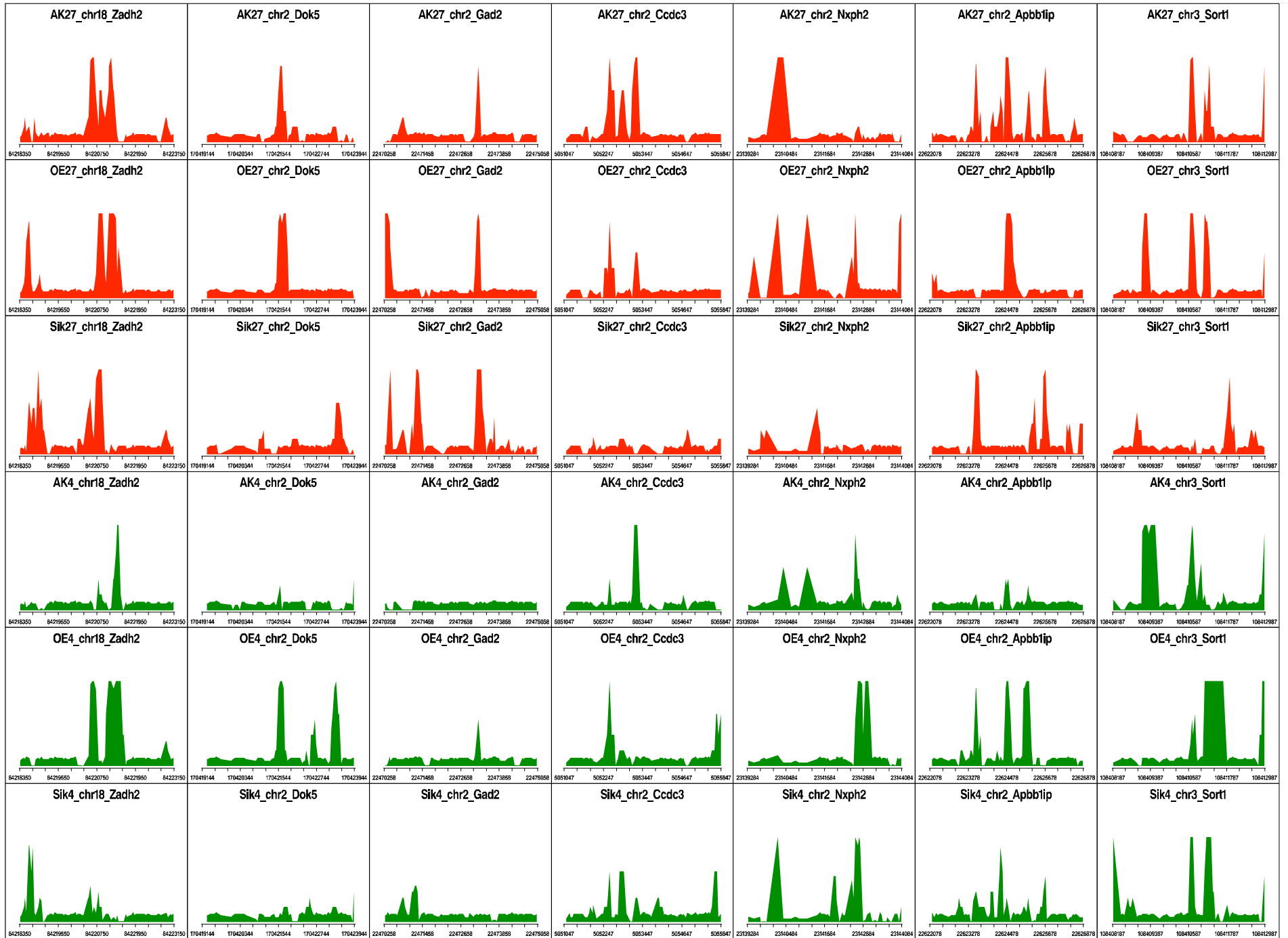

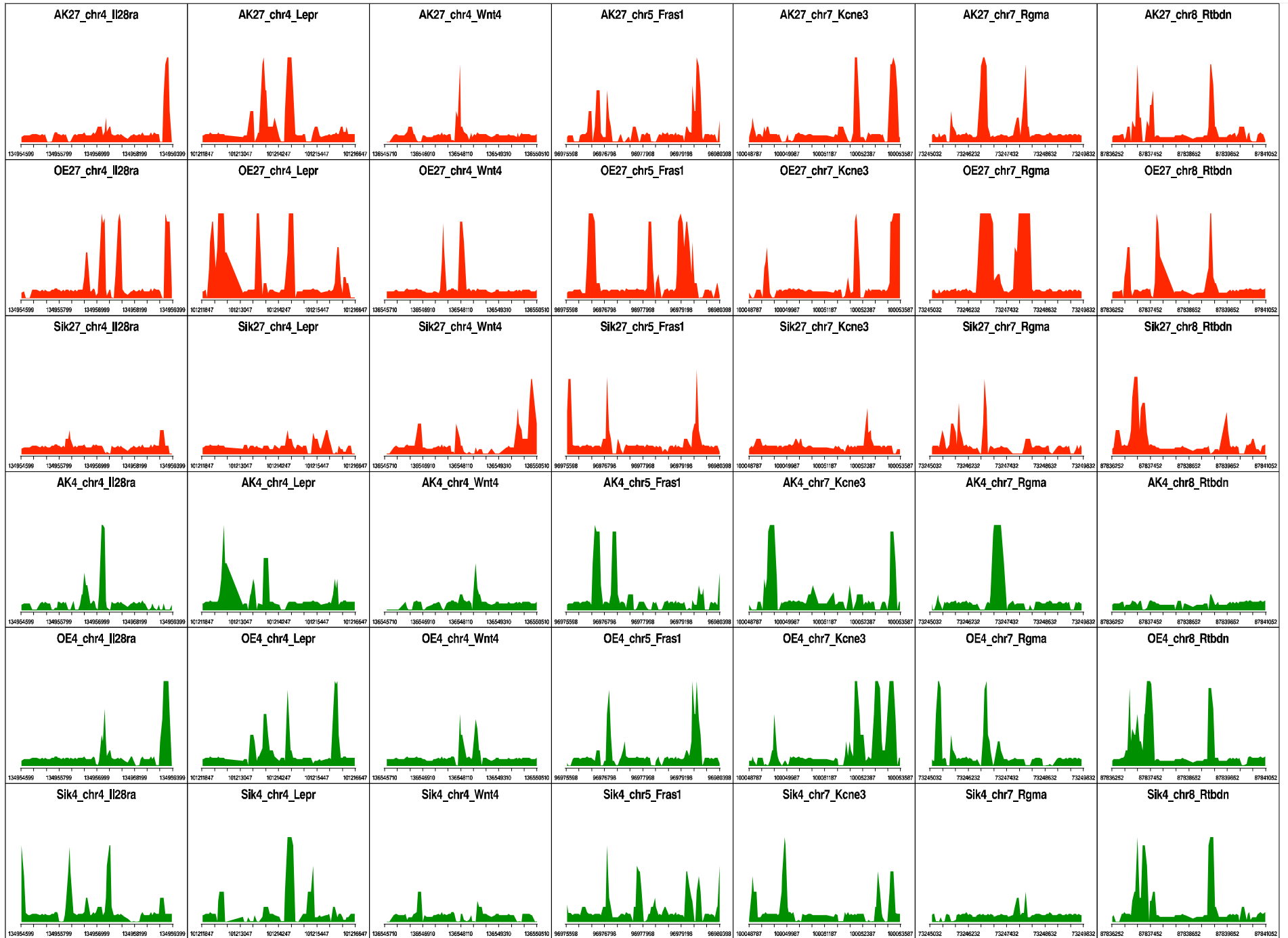

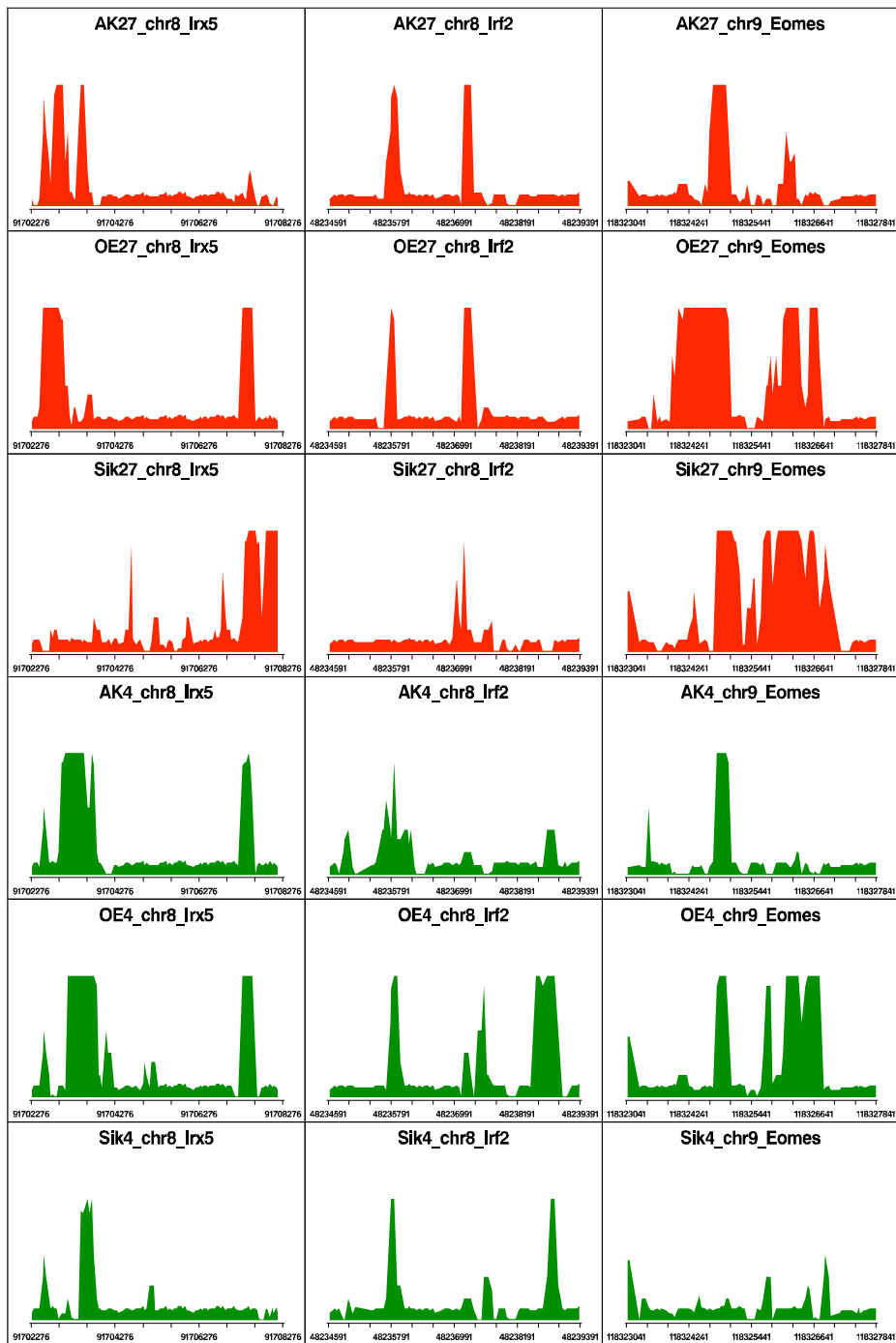

Supplement: Figure S8 — H3-K27me3 (red) and H3-K4me3 (green) patterns at promoters in which bivalency is gained upon c-Myc overexpression. DNA immunprecipitated with anti-H3-K4me3 or anti-H3-K27me3 was applied to the custom-designed array (see text). Enrichment ratios (log2 scale) for ChIP-enriched versus total input genomic DNA for 427 genes were processed by ACME and assigned p-values (−log10; y axis) identifying significant sites were plotted (see Methods). Red peaks present H3-K27me3 and green peaks present H3-K4me3. (1.11 MB PDF) [file pone.0007839.s008.pdf]

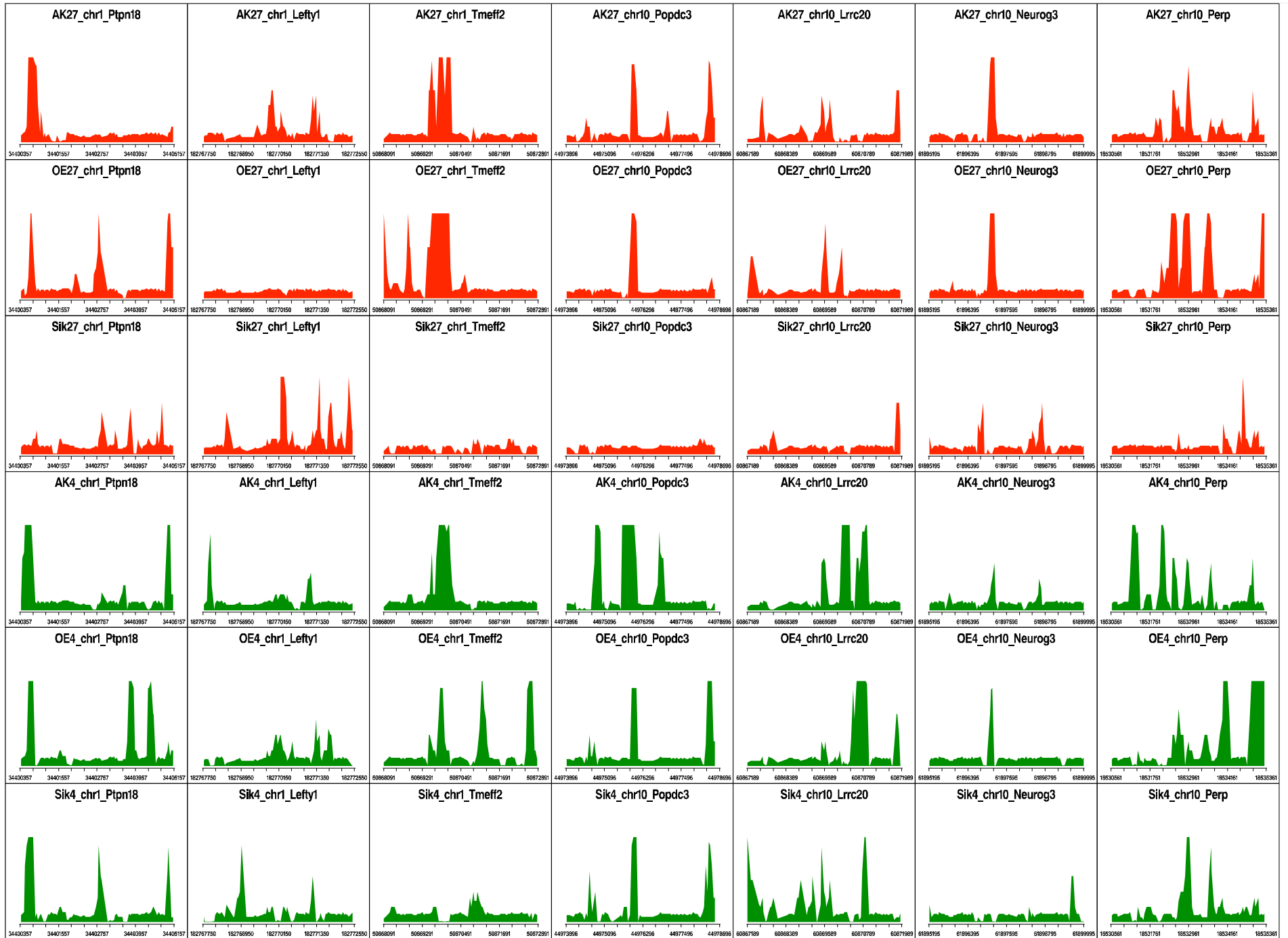

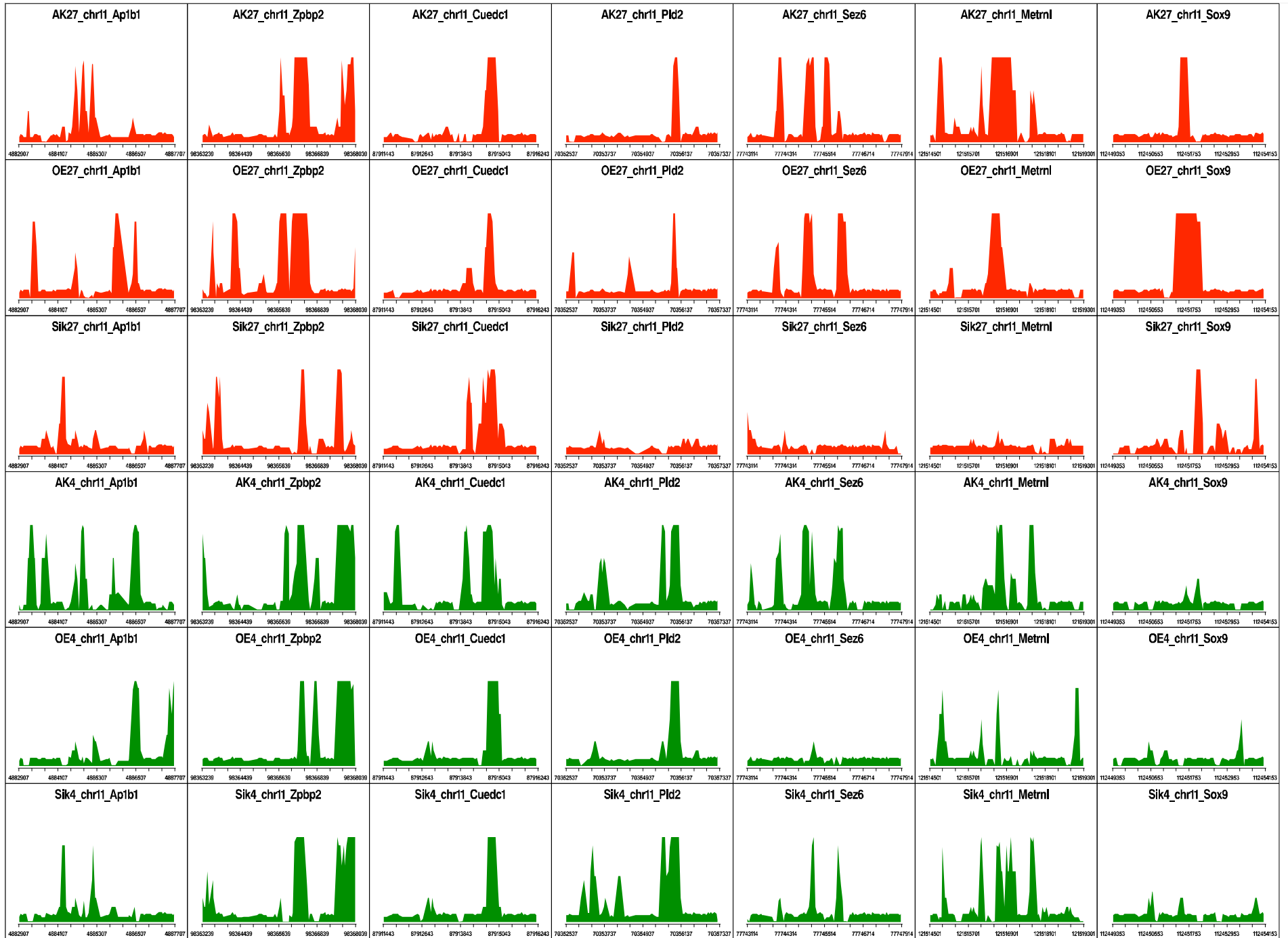

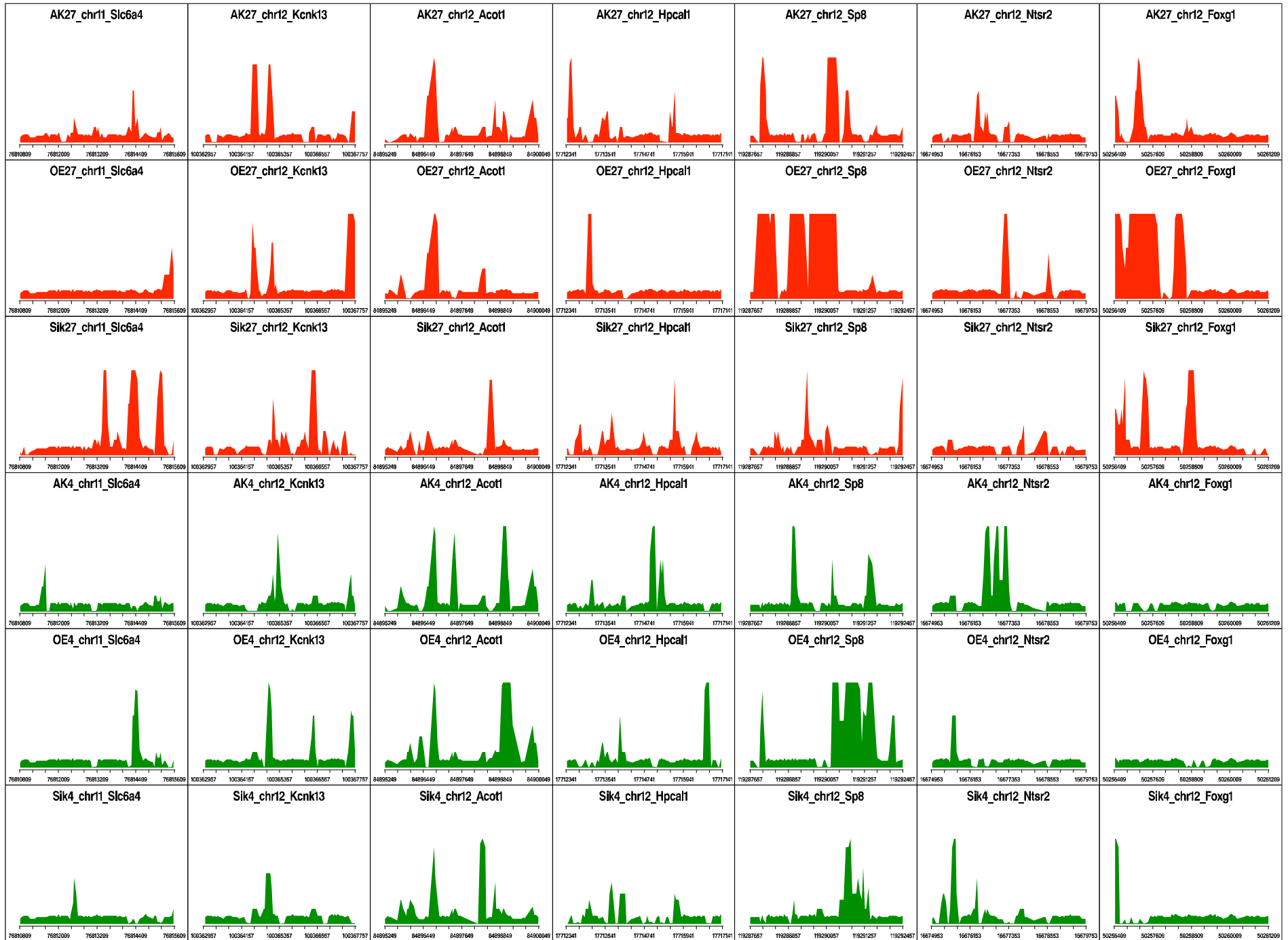

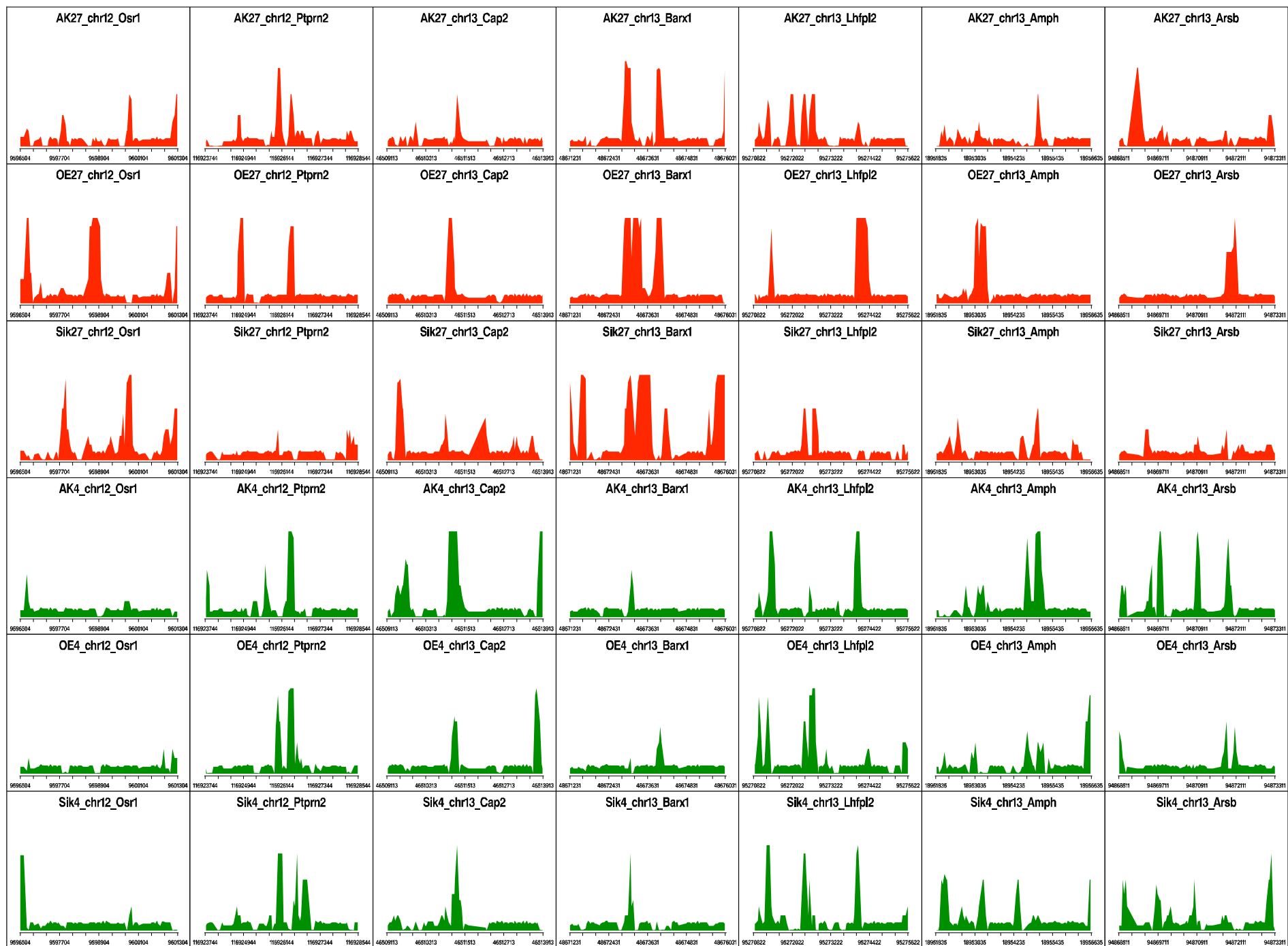

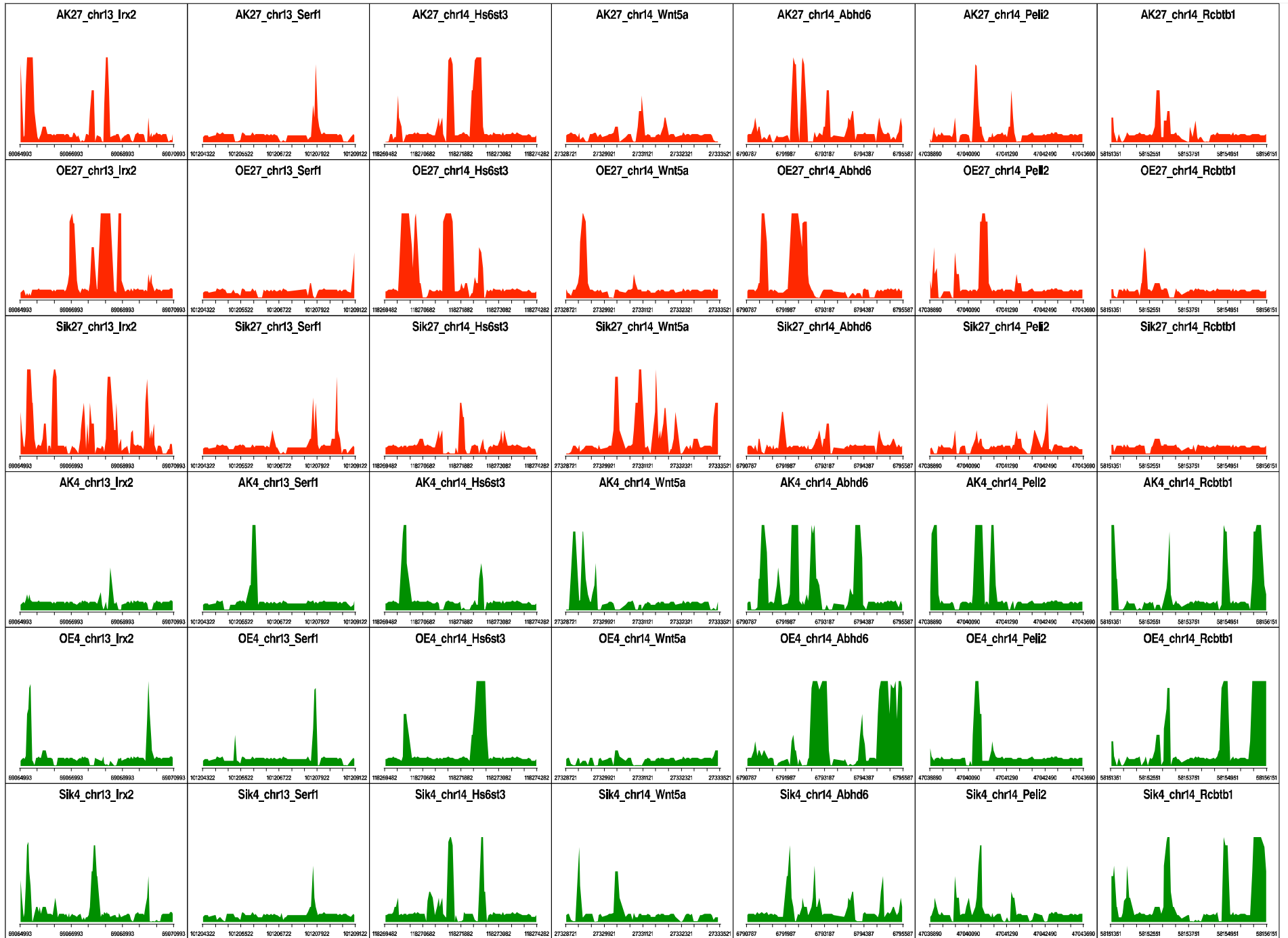

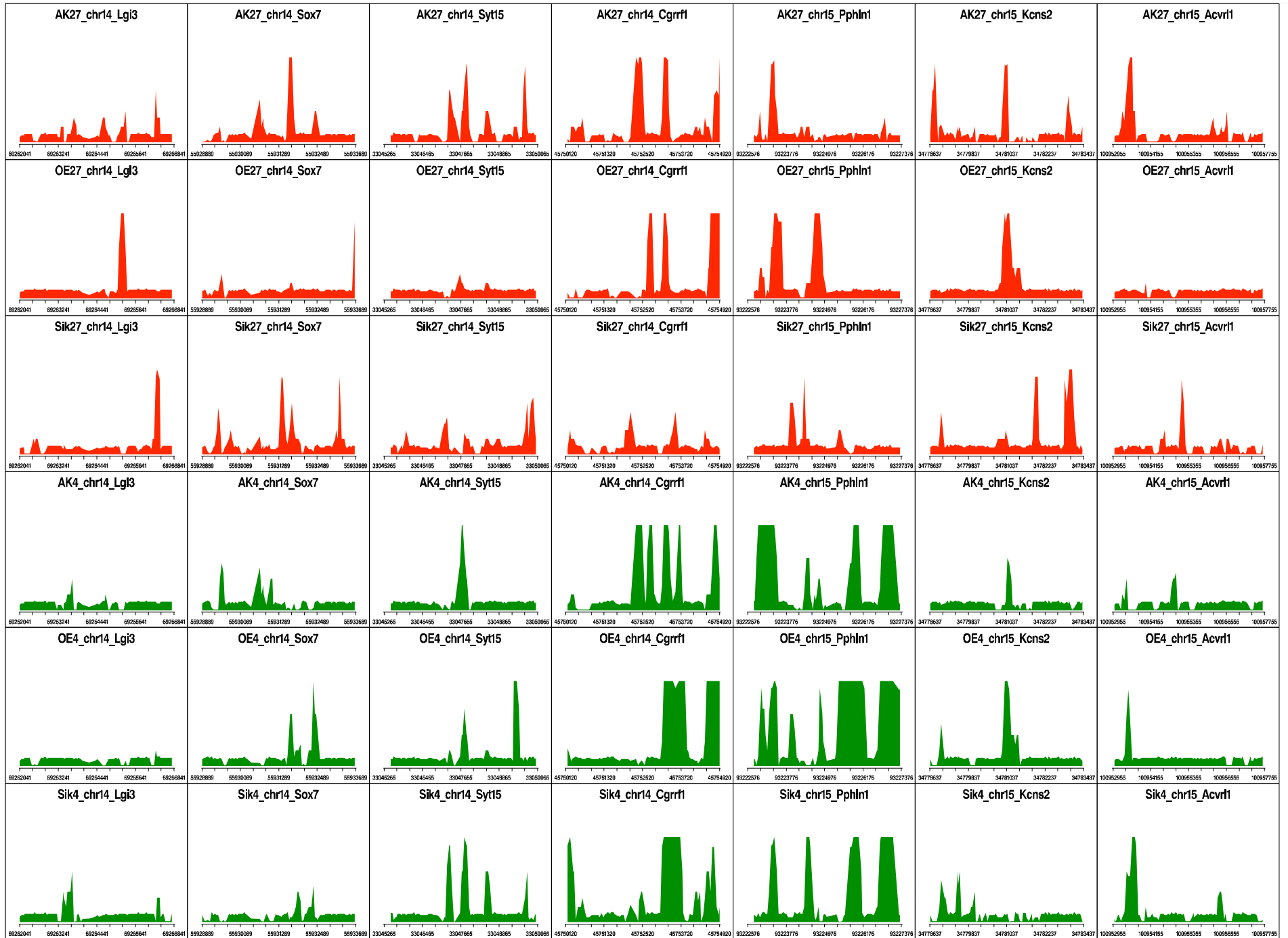

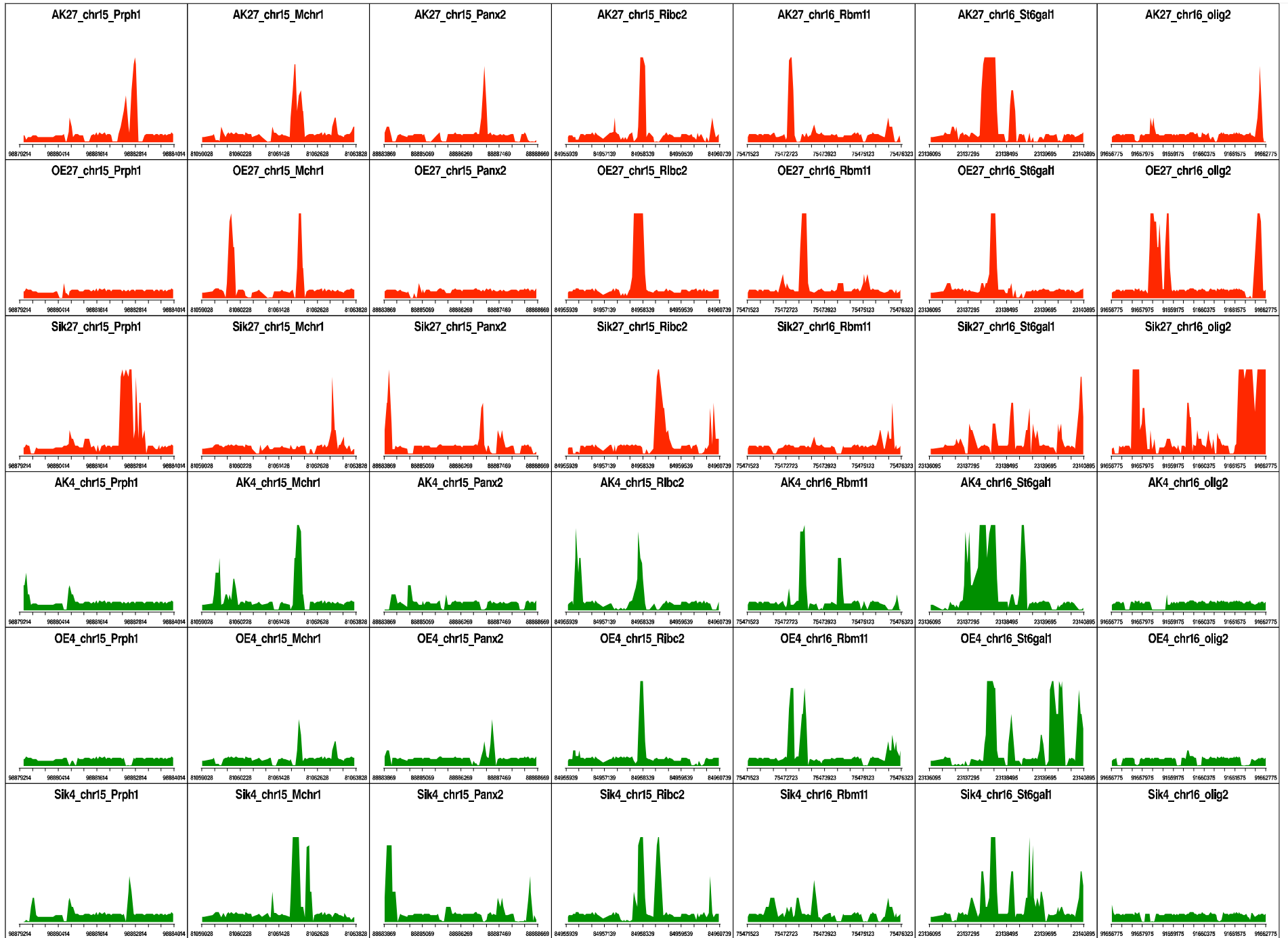

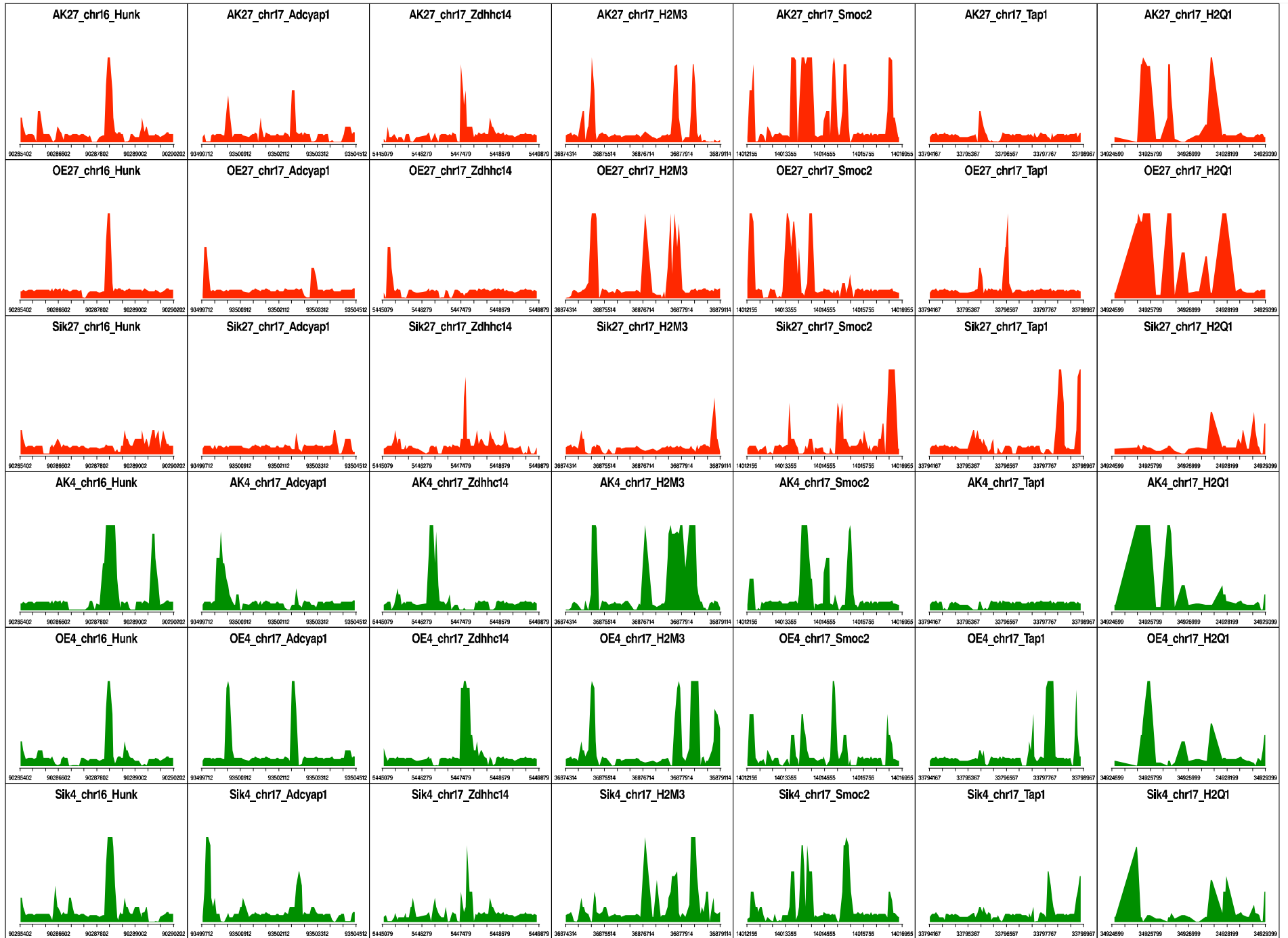

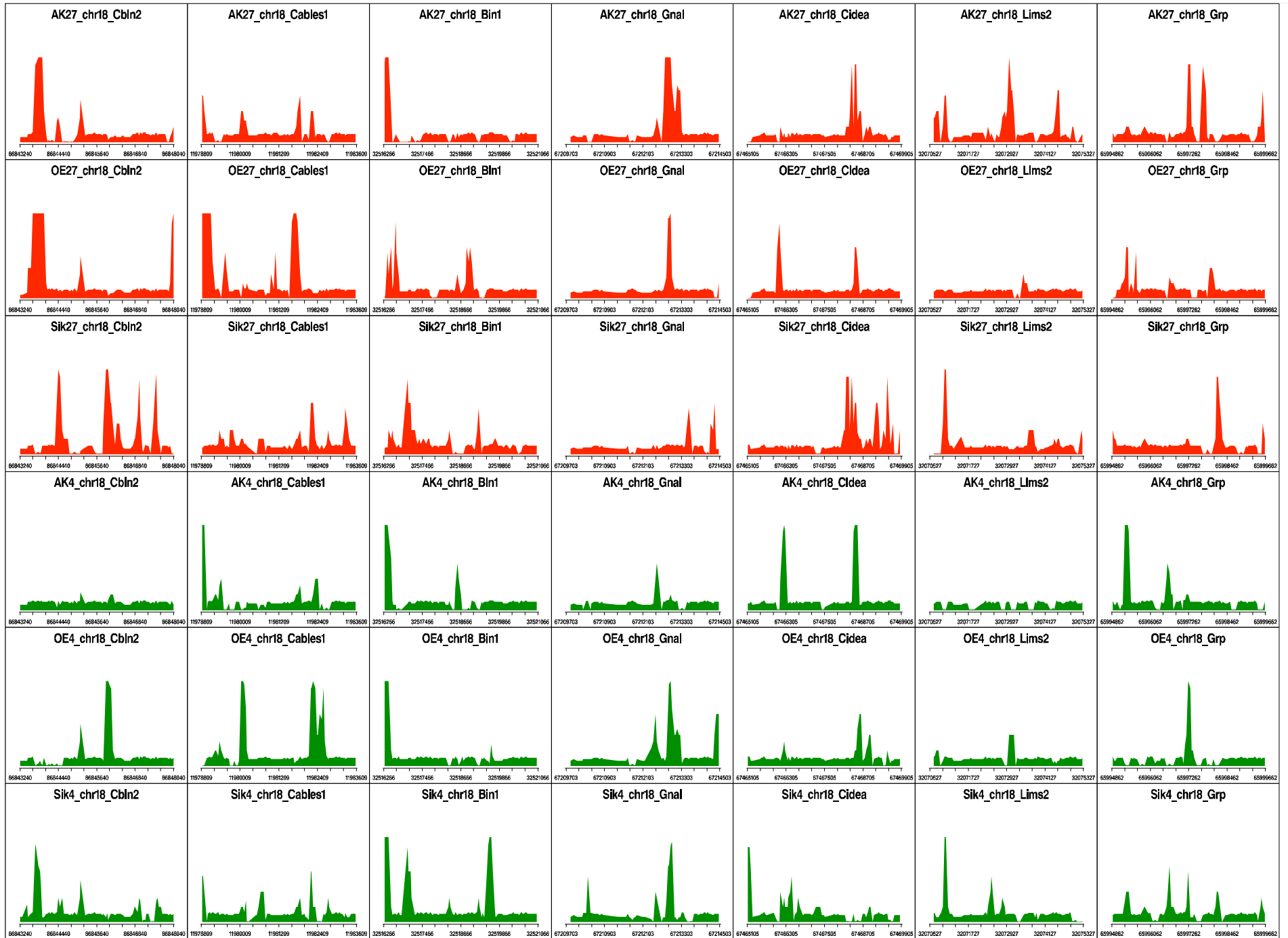

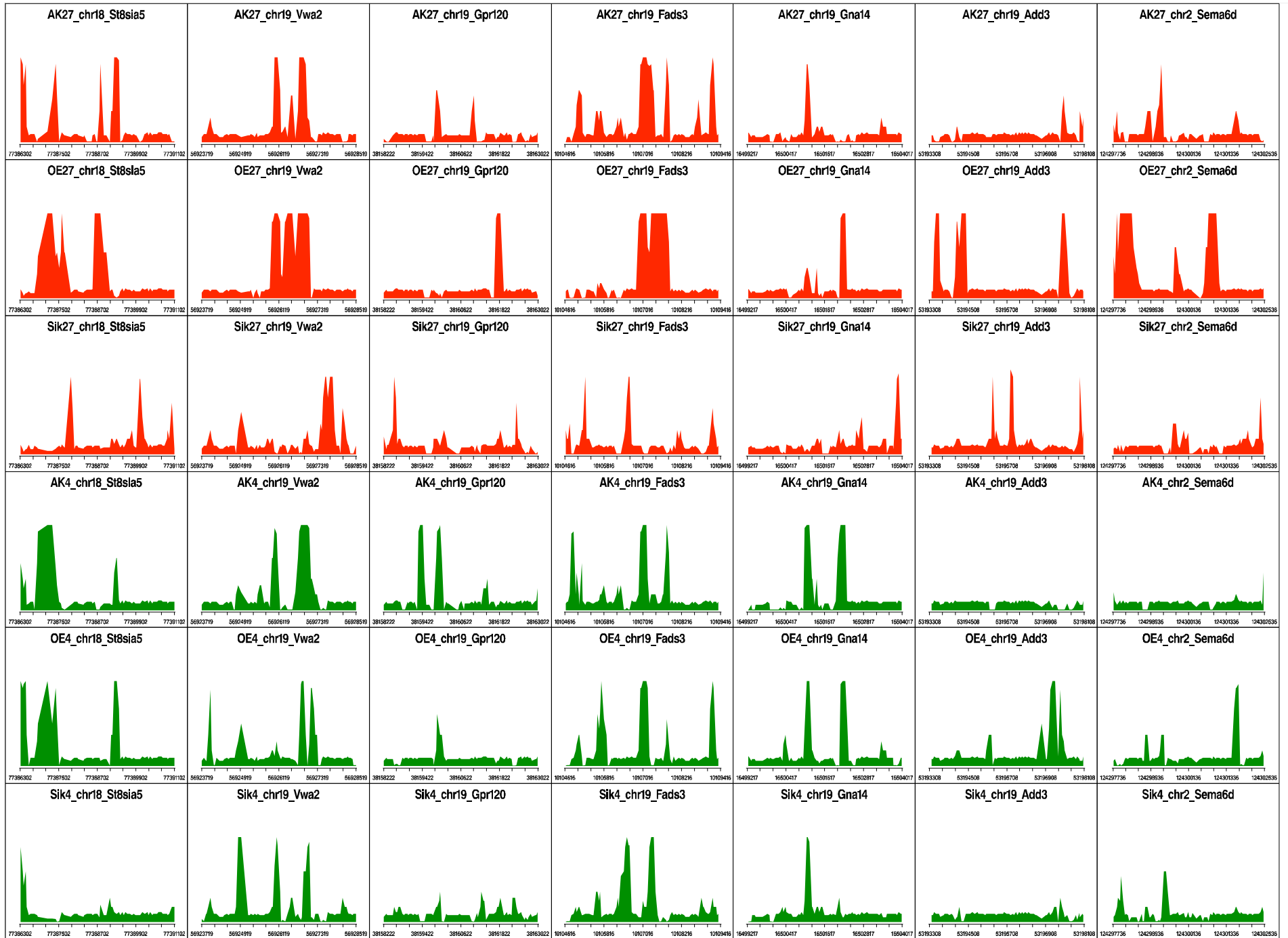

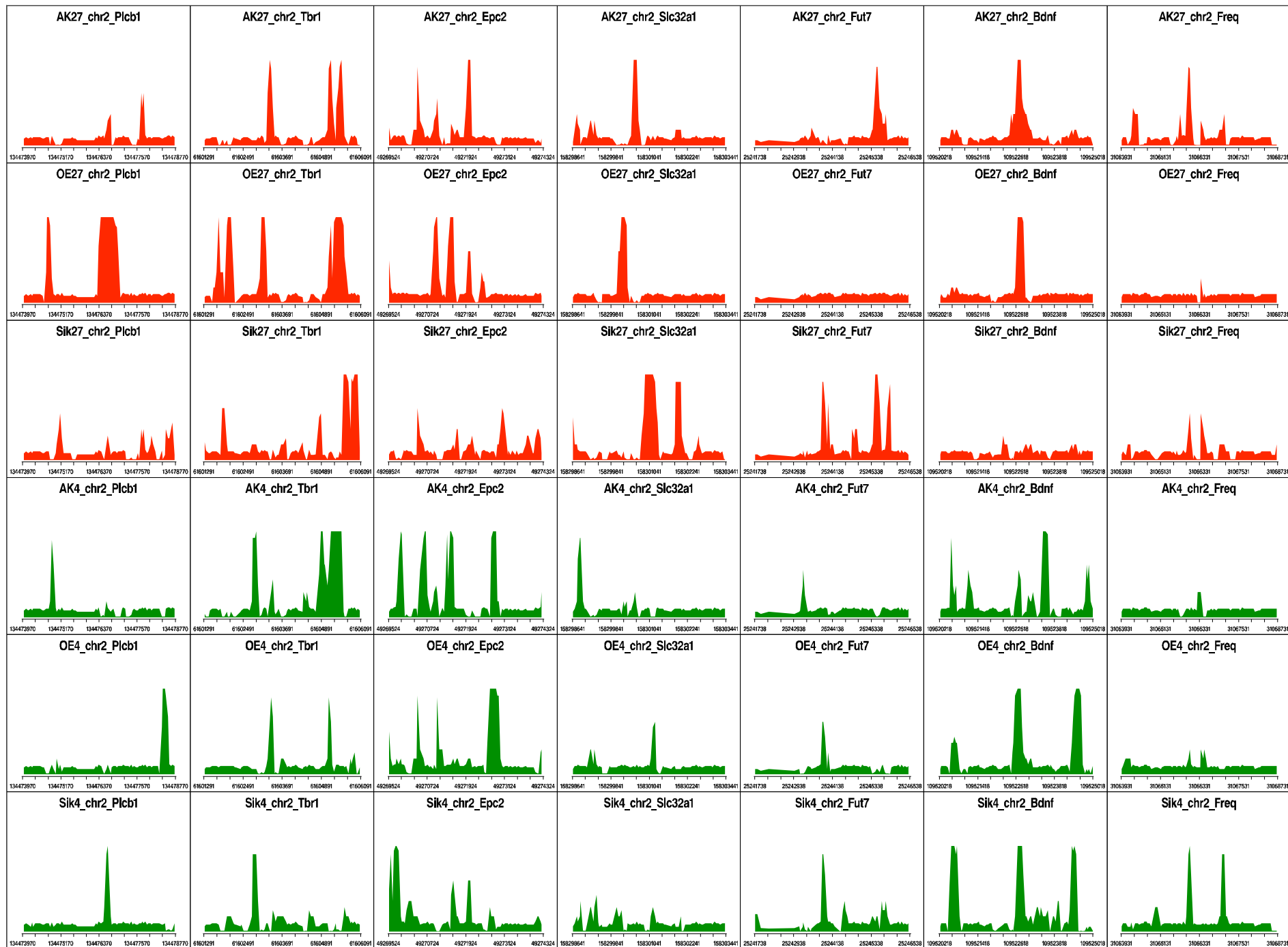

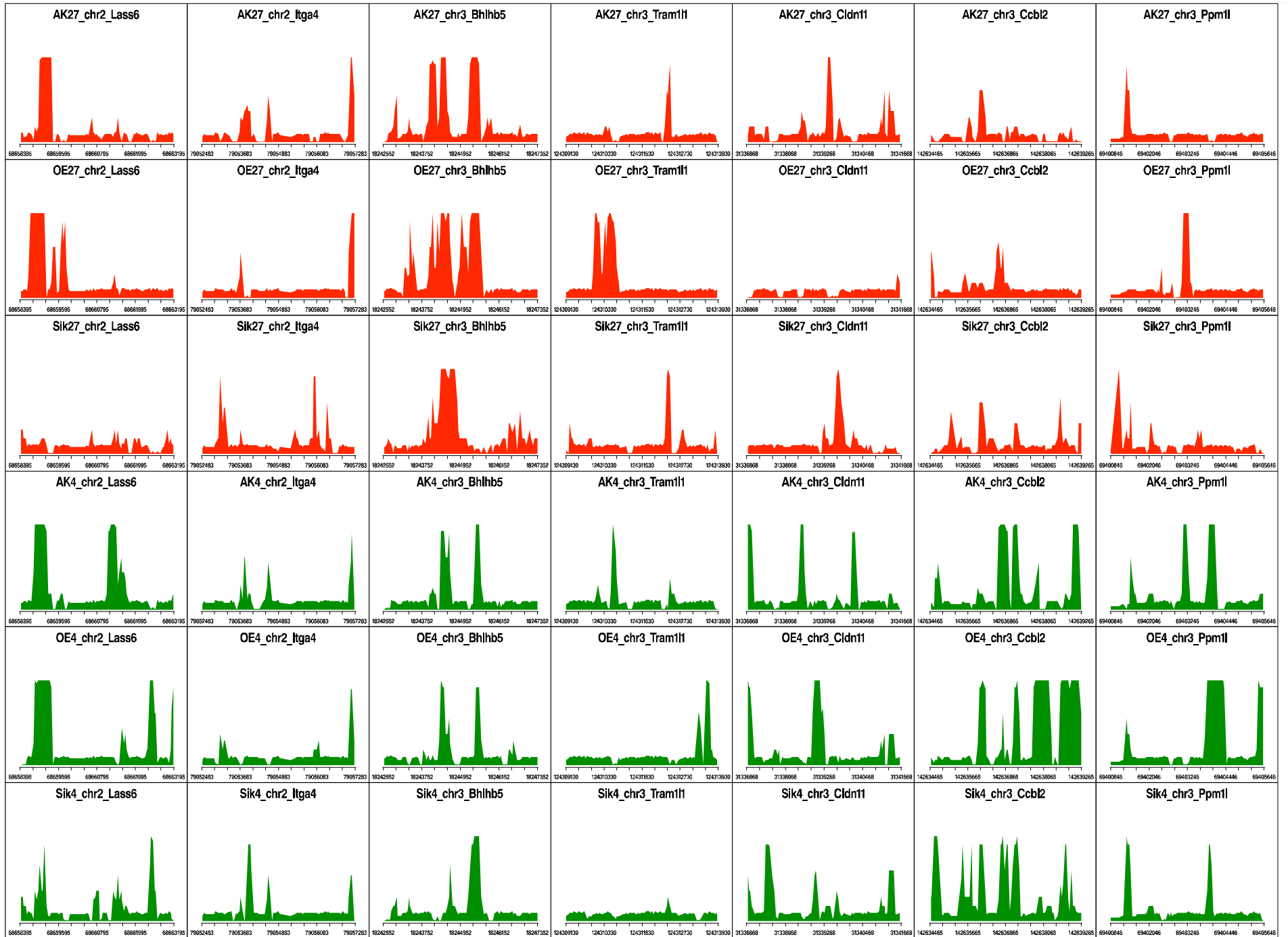

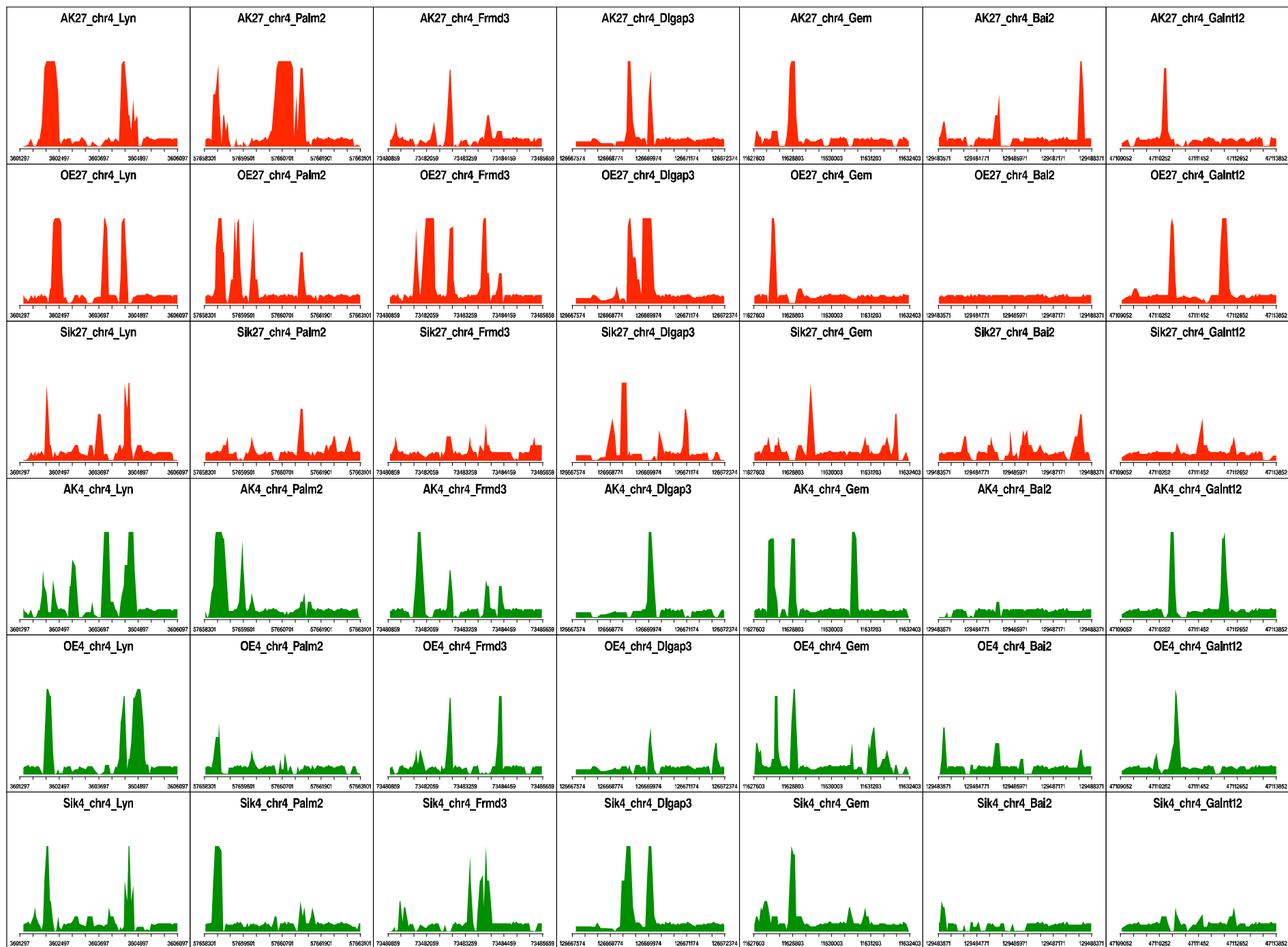

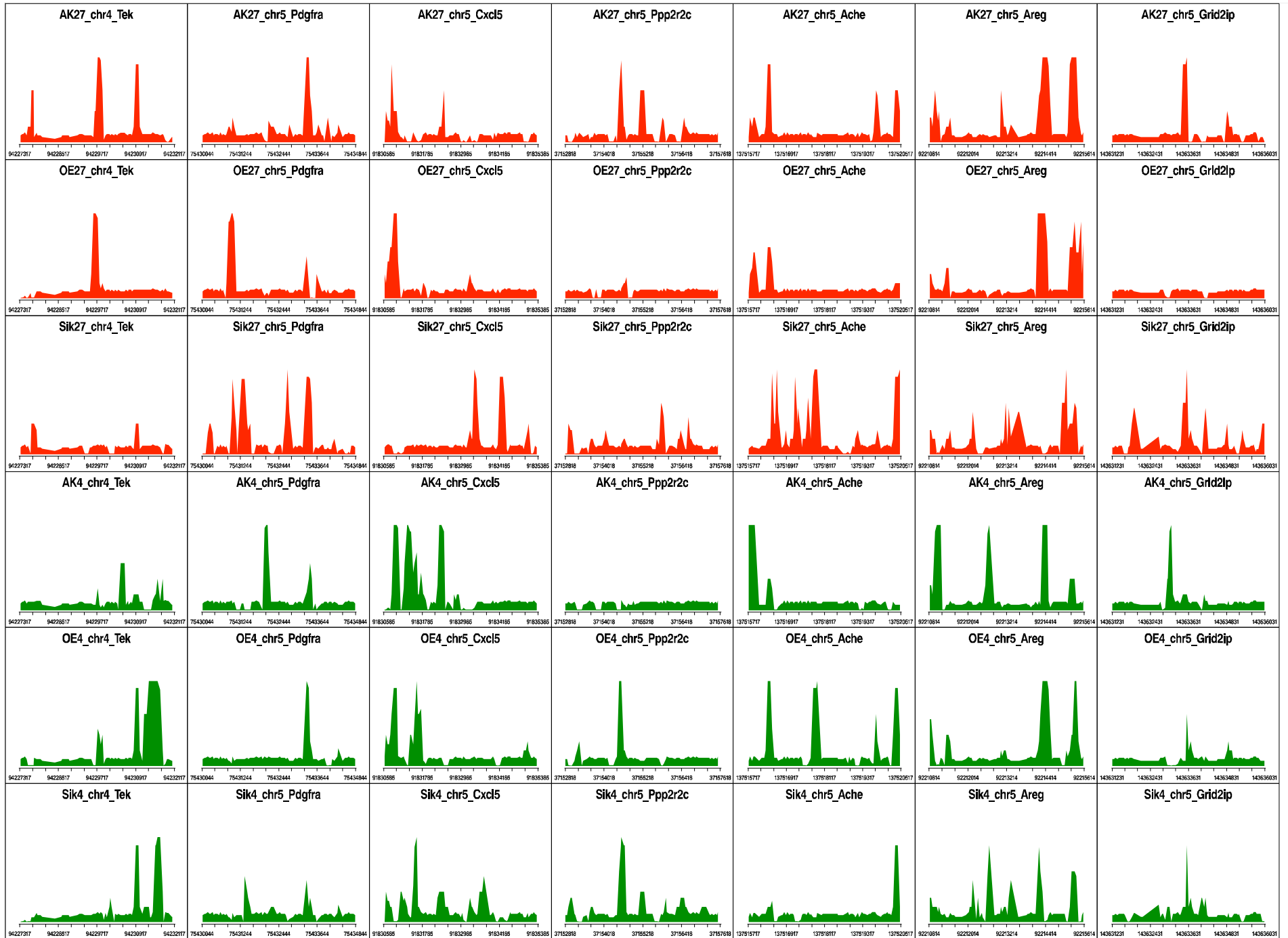

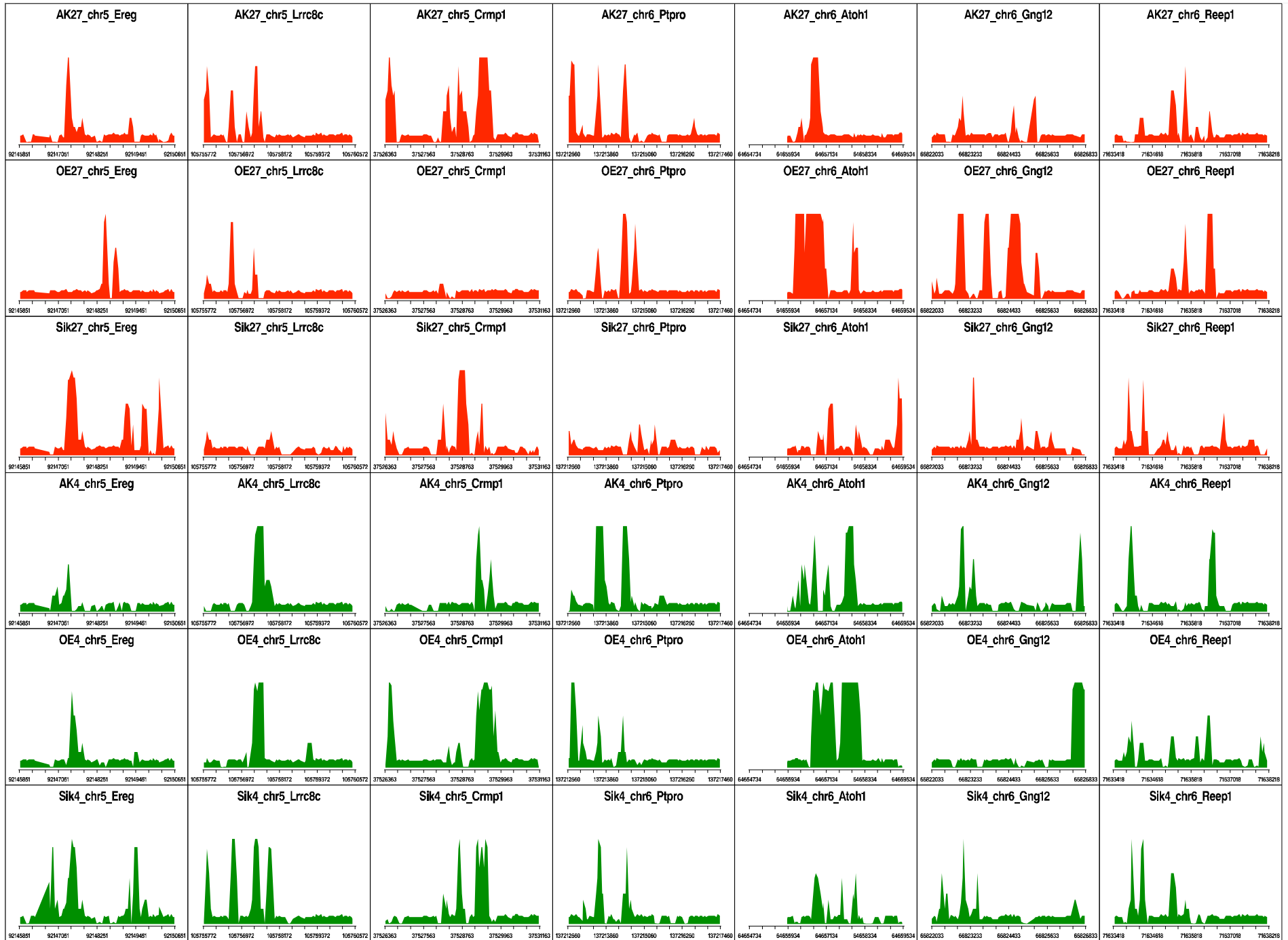

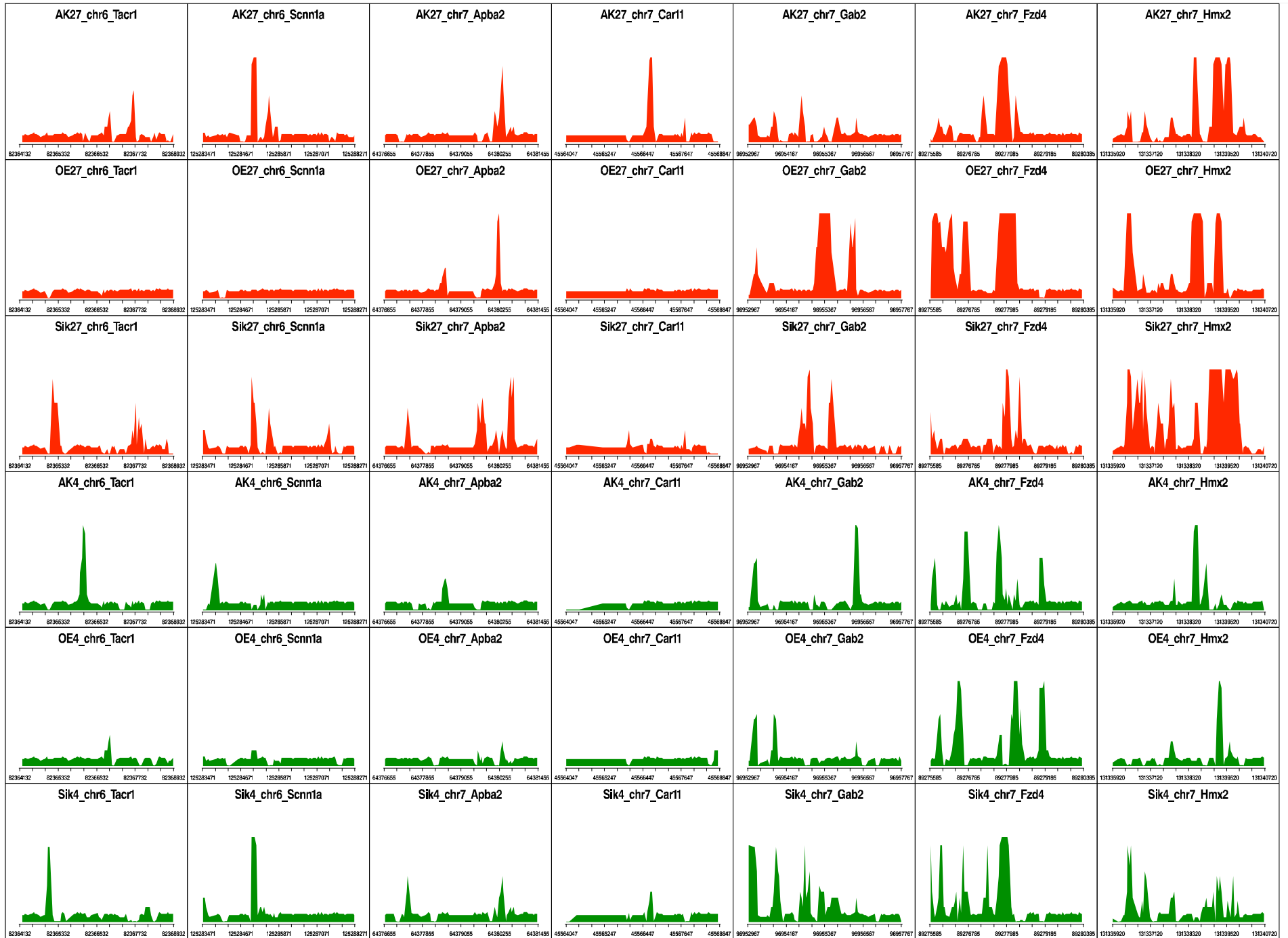

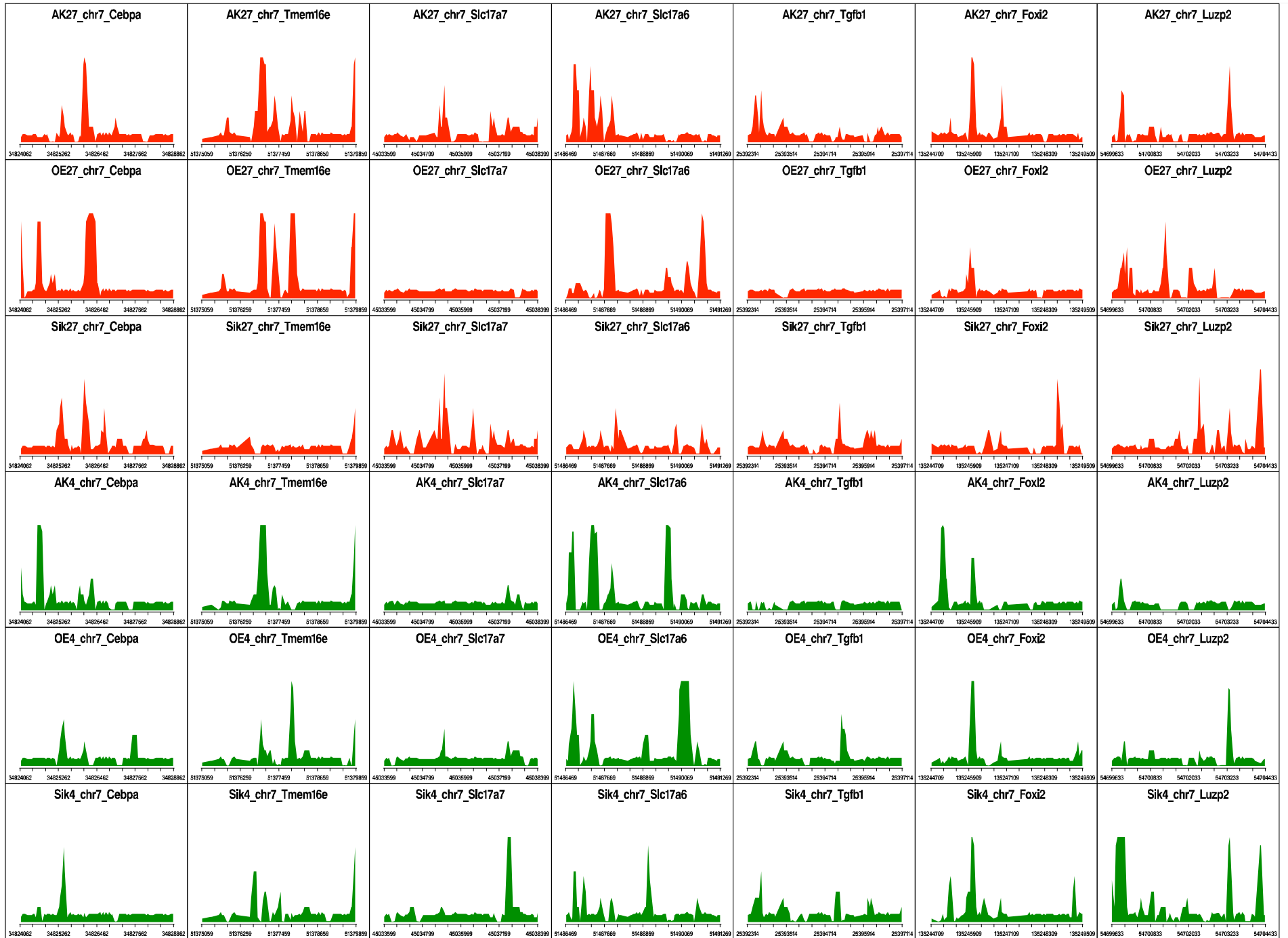

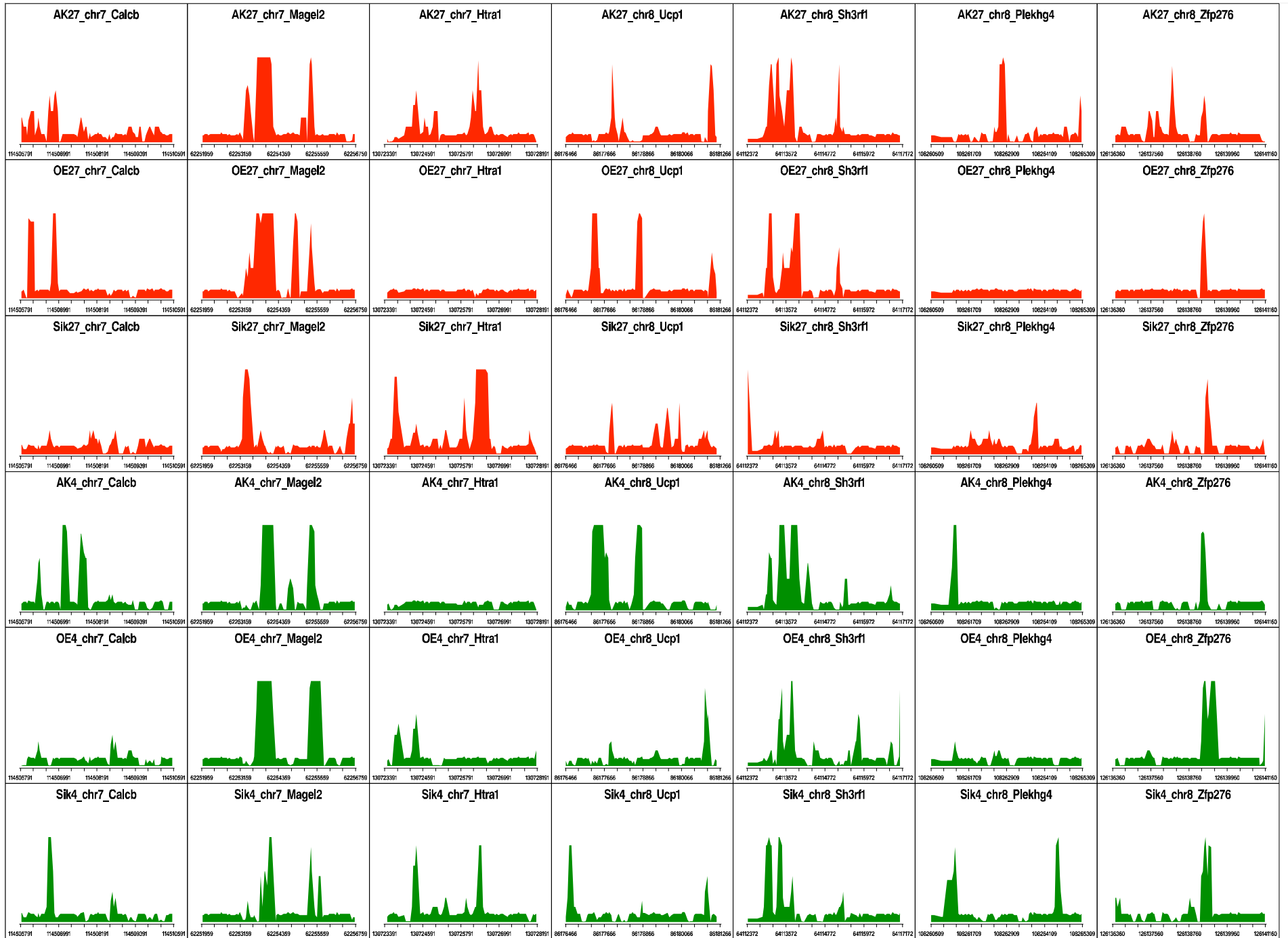

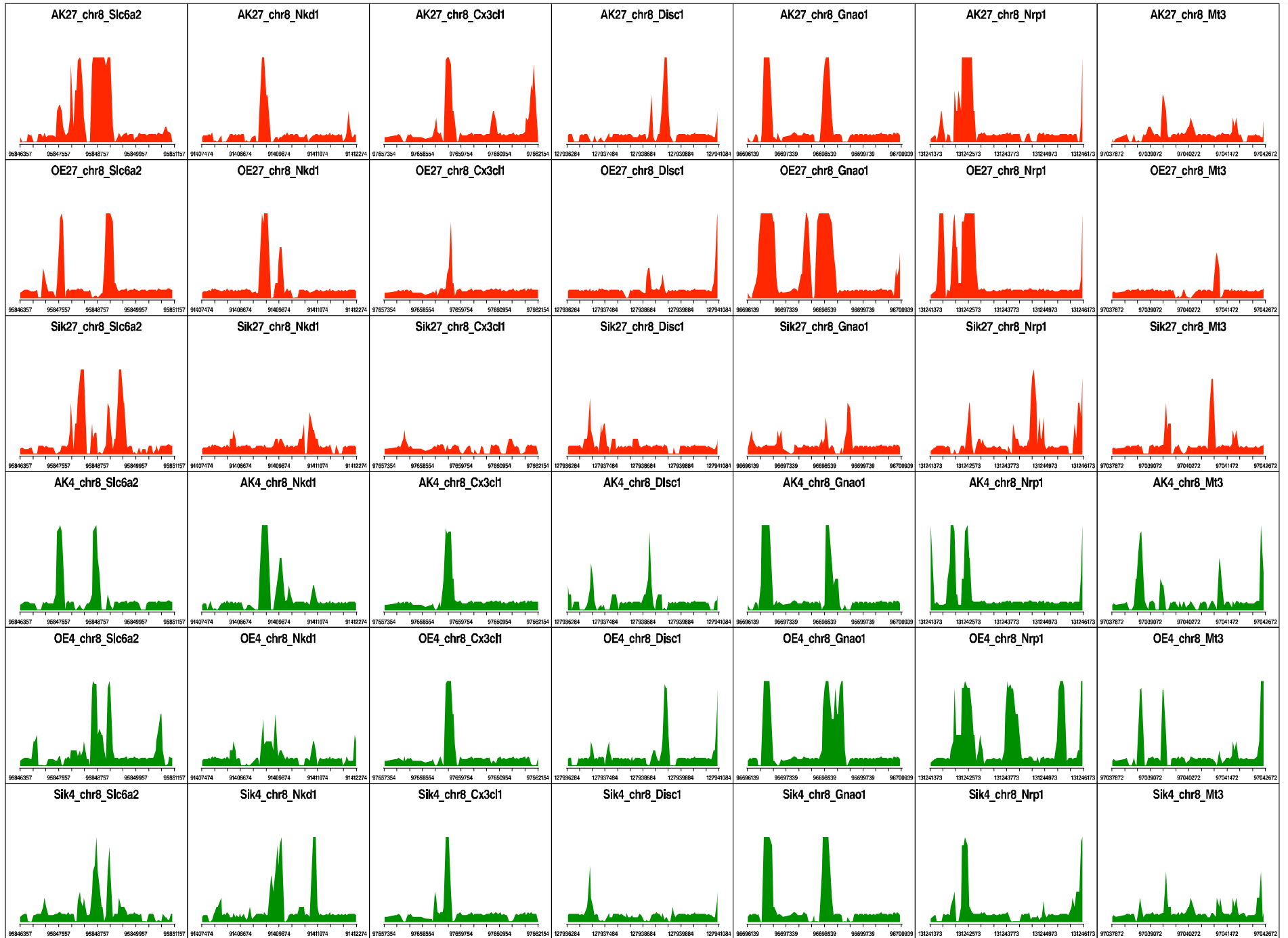

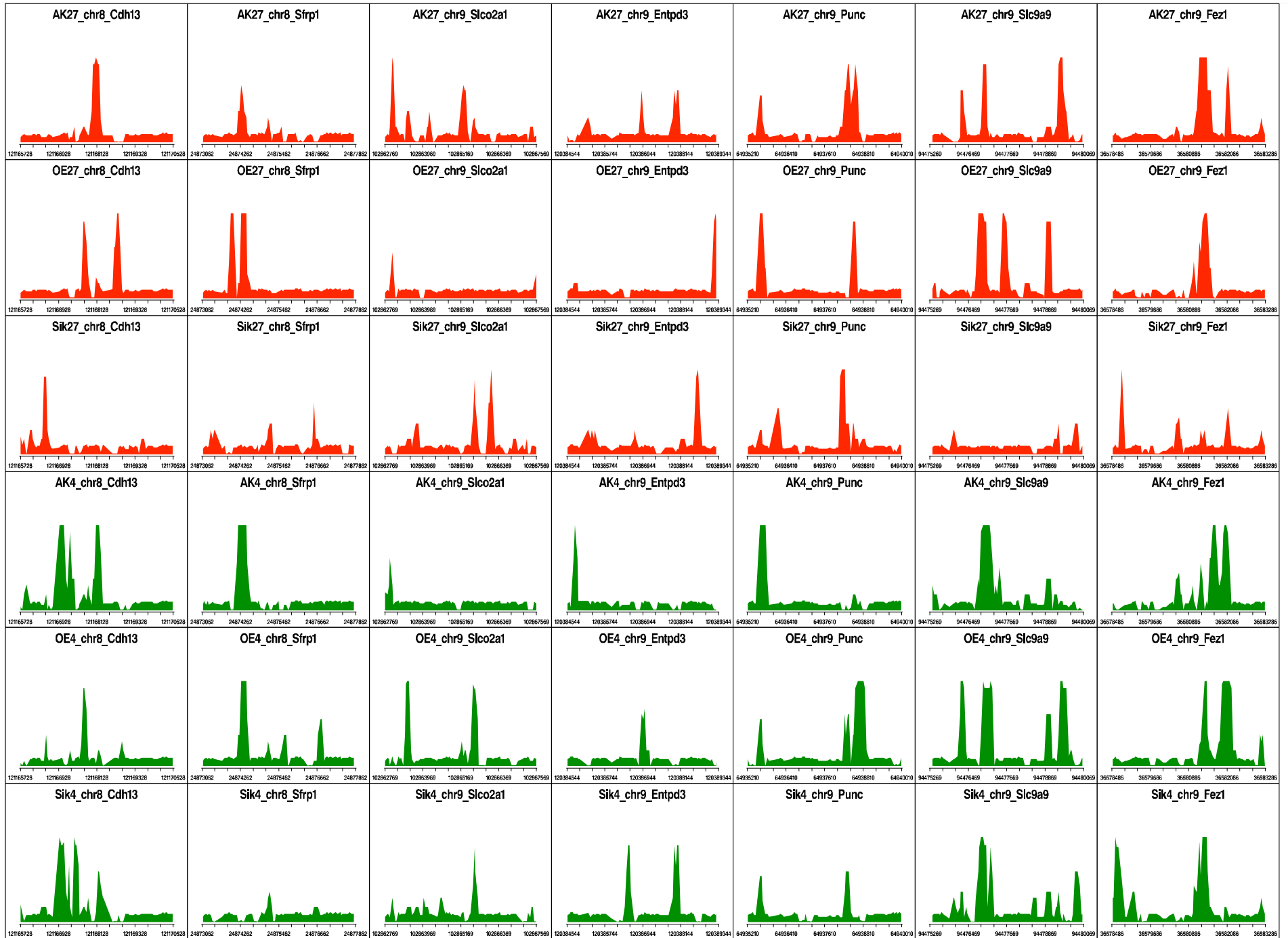

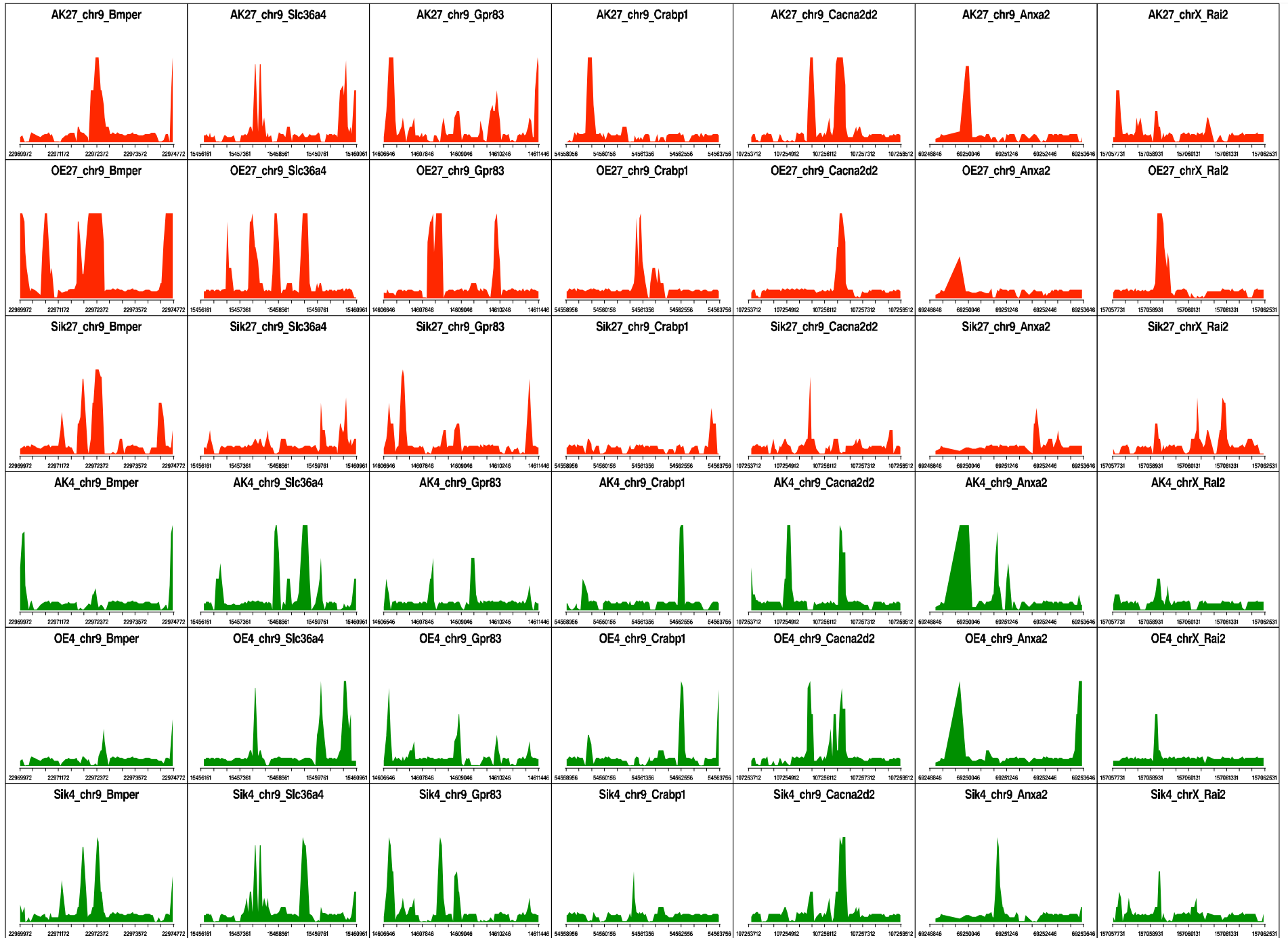

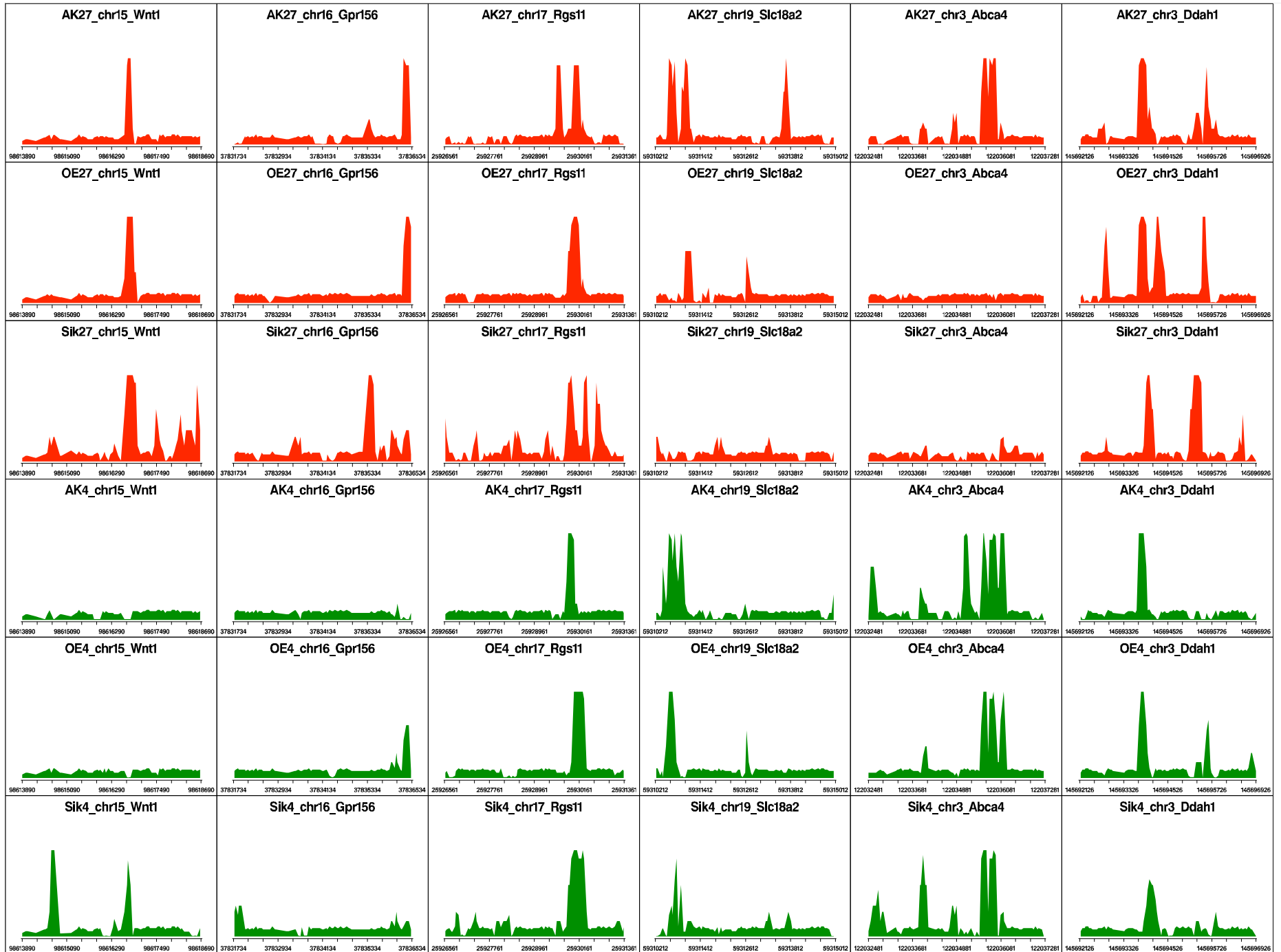

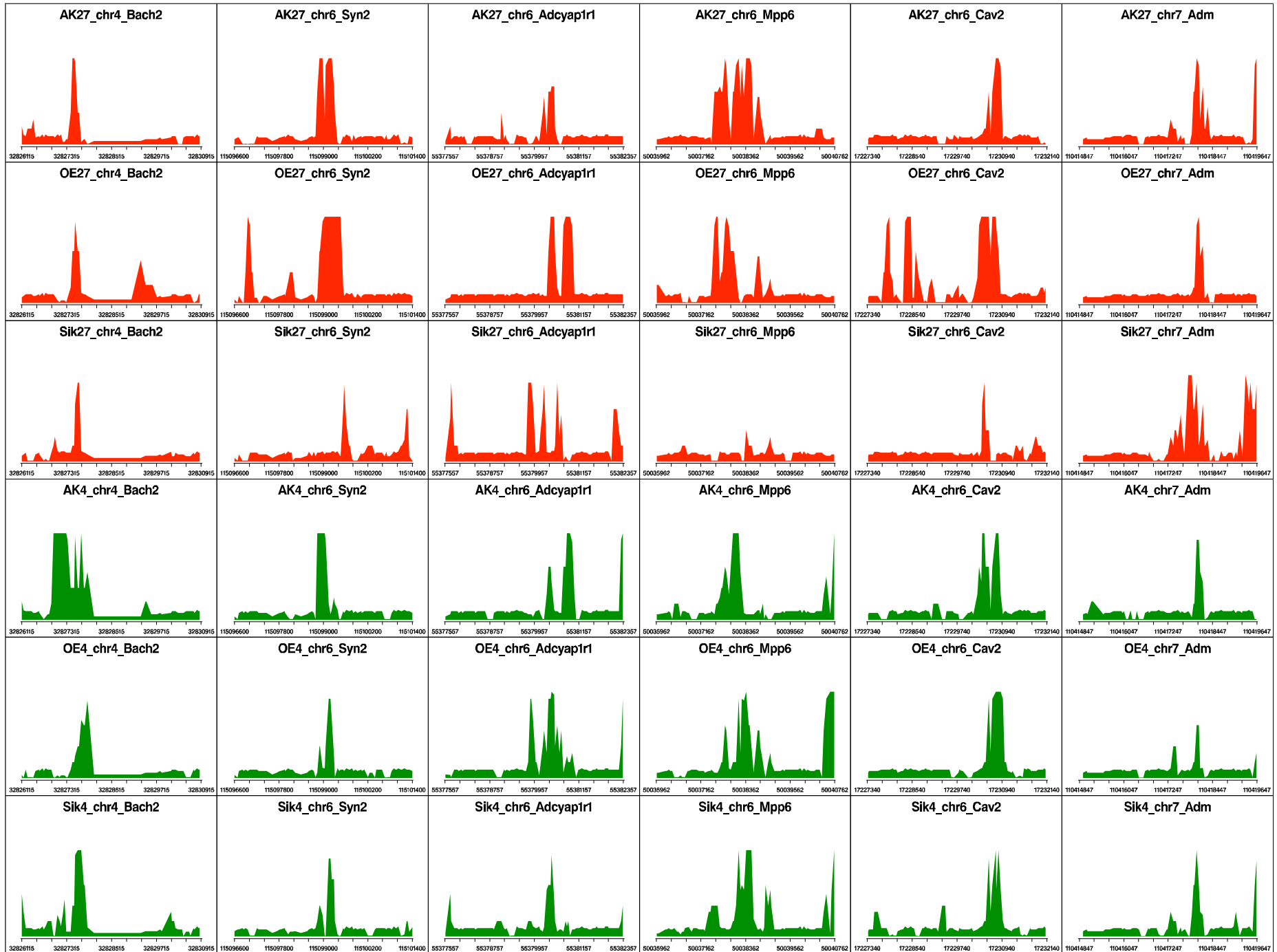

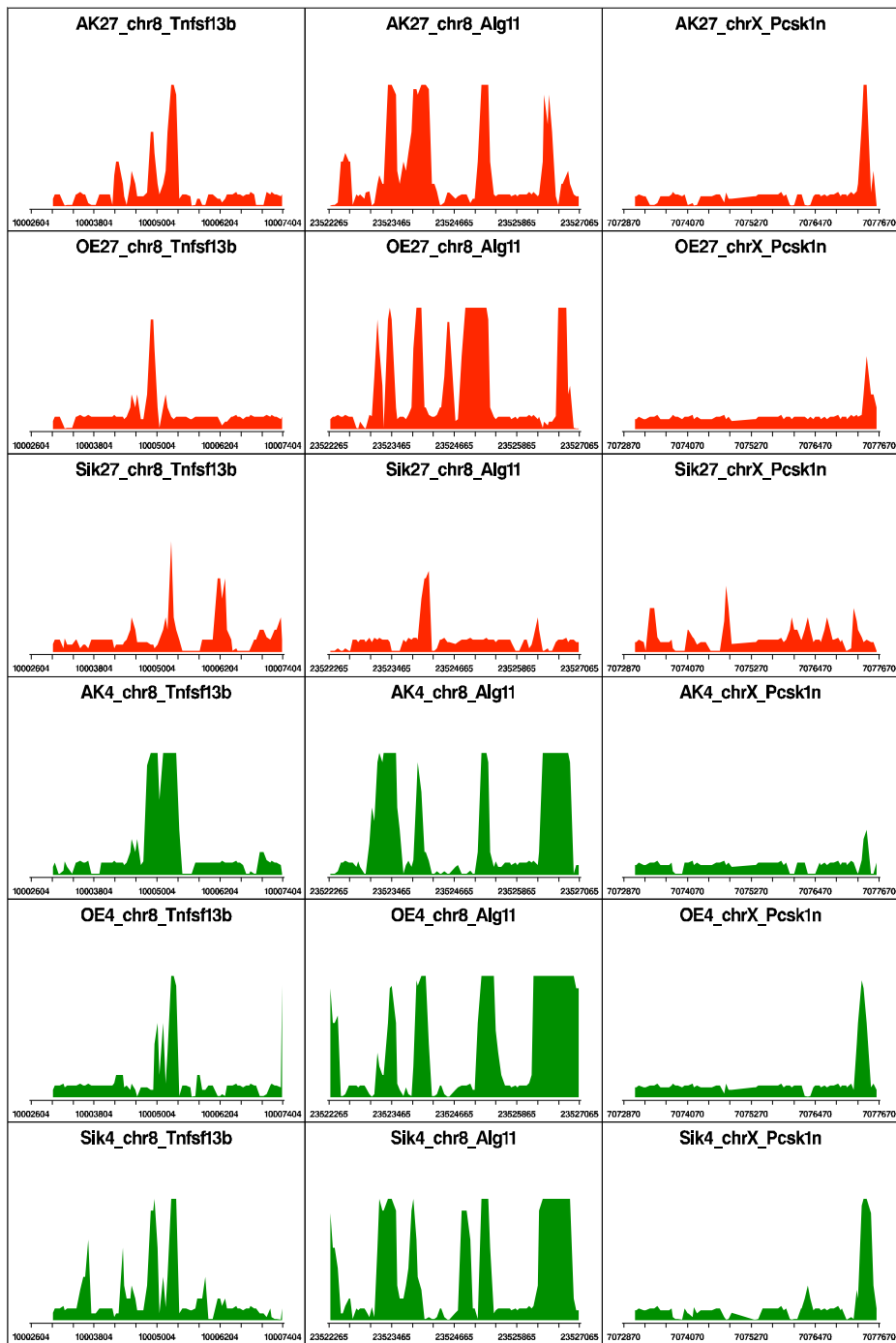

Supplement: Figure S9 — H3-K27me3 (red) and H3-K4me3 (green) patterns at promoters showing only small or no changes in H3-K27me3 and H3-K4me 3 pattern. DNA immunprecipitated with anti-H3-K4me3 or anti-H3-K27me3 was applied to the custom-designed array (see text). Enrichment ratios (log2 scale) for ChIP-enriched versus total input genomic DNA for 427 genes were processed by ACME and assigned p-values (−log10; y axis) identifying significant sites were plotted (see Methods). Red peaks present H3-K27me3 and green peaks present H3-K4me3. (5.05 MB PDF) [file pone.0007839.s009.pdf]

Jak2

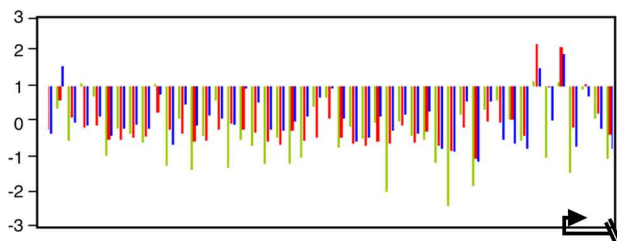

Onecut1

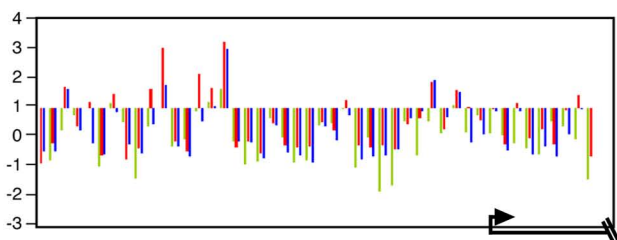

Hes1

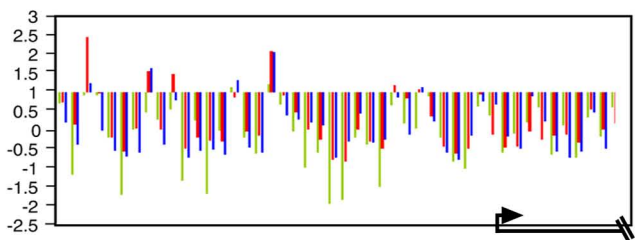

Gata6

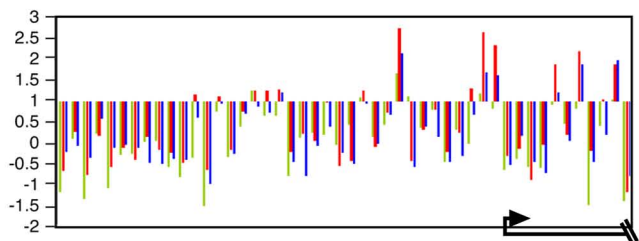

Supplement: Figure S10 — c-Myc binding sites on a subset of gene promoters in mES cells. c-Myc levels were manipulated by Lentiviral-delivered overexpression or knock-down of c-Myc in mES cells followed by anti-Myc ChIP-chip analysis on custom- designed arrays (see text). Unprocessed enrichment ratios (log2 scale) present ChIP-enriched versus total input genomic DNA (crosslinked, sonicated and processed identically to the ChIP sample) (y axis) for all probes within a genomic region 2kb upstream and 1kb downstream of the TSS. The TSS and direction of transcription are denoted by arrows at the bottom of the figure. Blue bars: endogenous c-Myc binding in WT mES cells, Red bars: Myc overexpressing mES cells, Green bars: Myc knock-down mES cells. Negative values occur when there is no enrichment in ChIP DNA relative to input. (0.16 MB PDF) [file pone.0007839.s010.pdf]
